# A food system transformation pathway reconciles 1.5 °C global warming with improved health, environment and social inclusion

**DOI:** 10.1038/s43016-025-01268-y

**Published:** 2025-12-19

**Authors:** Benjamin Leon Bodirsky, Felicitas Beier, Florian Humpenöder, Debbora Leip, Michael S. Crawford, David Meng-Chuen Chen, Patrick von Jeetze, Marco Springmann, Bjoern Soergel, Zebedee Nicholls, Jessica Strefler, Jared Lewis, Jens Heinke, Christoph Müller, Kristine Karstens, Isabelle Weindl, Miodrag Stevanović, Patrick Rein, Pascal Sauer, Abhijeet Mishra, Edna Johanna Molina Bacca, Alexandre C. Köberle, Xiaoxi Wang, Vartika Singh, Claudia Hunecke, Quitterie Collignon, Pepijn Schreinemachers, Simon Dietz, Ravi Kanbur, Jan Philipp Dietrich, Hermann Lotze-Campen, Alexander Popp

**Affiliations:** 1 Potsdam Institute for Climate Impact Research, Leibniz Association, Potsdam, Germany; 2 World Vegetable Center, Bangkok, Thailand; 3 Department of Agricultural Economics, Humboldt-Universität zu Berlin, Berlin, Germany; 4 Integrative Research Institute for Transformations of Human-Environment Systems (IRI THESys), Humboldt-Universität zu Berlin, Berlin, Germany; 5 London School of Hygiene and Tropical Medicine, London, UK; 6 University of Oxford, Oxford, UK; 7 International Institute for Applied System Analysis, IIASA, Laxenburg, Austria; 8 Melbourne Climate Futures, University of Melbourne, Melbourne, Victoria Australia; 9 School of Geography, Earth and Atmospheric Sciences, University of Melbourne, Melbourne, Victoria Australia; 10 Climate Resource, Melbourne, Victoria Australia; 11 International Food Policy Research Institute, Washington, DC USA; 12 Grantham Institute for Climate Change and Environment, Imperial College London, London, UK; 13 Centre for Climate Finance and Investment, Imperial College Business School, London, UK; 14 Instituto Dom Luiz (IDL), Faculdade de Ciências, Universidade de Lisboa, Lisbon, Portugal; 15 China Academy for Rural Development, School of Public Affairs, Zhejiang University, Hangzhou, China; 16 International Food Policy Research Institute, New Delhi, India; 17 London School of Economics, London, UK; 18 Cornell University, New York, NY USA; 19 Faculty of Organic Agricultural Sciences, University of Kassel, Witzenhausen, Germany

**Keywords:** Socioeconomic scenarios, Climate-change mitigation, Environmental economics, Sustainability

## Abstract

The improvement of the global food system requires a thorough understanding of how specific measures may contribute to the system’s transformation. Here we apply a global food and land system modelling framework to quantify the impact of 23 food system measures on 15 outcome indicators related to public health, the environment, social inclusion and the economy, up to 2050. While all individual measures come with trade-offs, their combination can reduce trade-offs and enhance co-benefits. We estimate that combining all food system measures may reduce yearly mortality by 182 million life years and almost halves nitrogen surplus while offsetting negative effects of environmental protection measures on absolute poverty. Through joint efforts, including measures outside the food system, the 1.5 °C climate target can be achieved.

## Abstract (web summary)

This study applies a global food and land system modelling framework to quantify the impact of 23 food system measures on 15 outcome indicators related to public health, the environment, social inclusion and the economy—from today until 2050.

## Main

The global food system falls short of long-term targets for global health, environmental conditions and social inclusion^1,2^. Malnutrition is the leading global health risk, causing 11 million deaths and the loss of 255 million disability-adjusted life years per year^3,4^. The decline in undernutrition is stagnating, and overnutrition-related health risks are rapidly increasing^2^. The food system is the main driver of biodiversity loss, nitrogen pollution and water withdrawals, and contributes about one-third of global greenhouse gas (GHG) emissions^5,6^. Baseline scenarios produced by global food system models project a continuing decline in environmental conditions^7,8^. Approximately 1.3 billion people receive jobs and income from the food system, mostly in agriculture, yet often under precarious conditions^9,10^. At the same time, income inequality in combination with the regressive effects of food as a necessity good makes food expenditures an important determinant of poverty. Food expenditures, in turn, are highly sensitive to shocks such as income losses during the COVID-19 pandemic or food price spikes after Russia’s invasion of Ukraine^10–12^.

Concrete and plausible pathways towards a desirable future can guide transformative change by fostering a debate about a shared vision^13–15^, whereby we adopt the Intergovernmental Science-Policy Platform on Biodiversity and Ecosystem Services (IPBES) definition of transformative change as a “fundamental, system-wide reorganization across technological, economic and social factors, including paradigms, goals and values”^16^ and the Intergovernmental Panel on Climate Change (IPCC) definition of pathways as goal-oriented scenarios^17^. The 2022 UN Food System Summit highlighted the need for sustainable transformation pathways that cover food systems comprehensively^1,18^. Such pathways can serve as benchmarks for measuring progress, facilitate coordination and allow for debates on the effectiveness of measures and potential trade-offs with other desirable outcomes^13–15,19^. Comprehensive pathways are scarce in the literature^16^, yet food system models that use integrated assessment methods can derive such pathways: they can simulate long-term and large-scale transformations, ensure plausibility and internal consistency, and integrate the effects of measures across multiple parts of the food system^7,20,21^. As such, they can inform ongoing policy processes such as the development of national food system pathways^22^.

Here we provide a food system transformation (FST) pathway, which highlights the possibility of an alternative global food system that can achieve substantive simultaneous enhancement of global health, environmental conditions and social inclusion in and through the food system. We propose FST as one possible normative benchmark for a desirable future of the global food system. Our assessment starts with a reference baseline scenario (BASE_SSP2_) following the middle-of-the-road Shared Socioeconomic Pathway 2 (SSP2)^23^. We estimate 15 social-welfare-related outcome indicators (Table 1) that comprehensively span the four dimensions of health, environment, social inclusion and the economy^24^. As the contribution of these outcomes to overall social welfare is uncertain and involves making subjective value judgements, the indicators are presented side by side and not ranked or aggregated. We then analyse the impact of 23 food system measures (FSMs), both individually and in packages. They include measures such as higher consumption of pulses, protection of biodiversity hotspots from land-use change, increased manure recycling, minimum wages and other measures that have been suggested for transforming the global food system towards better health, environment and inclusion outcomes (Table 2). Combining all FSMs in the context of the reference scenario SSP2 leads to the FST_SSP2_ pathway. We also assess the cross-sector impacts of five sustainability transformations outside the food system (CrossSector), such as more equitable economic growth and human development, an energy transition towards renewables and increased timber use as a construction material. Combining the FST with the CrossSector impacts defines our FST in the context of a sustainable development pathway (FST_SDP_). All FSMs and CrossSector measures are described in detail in Table 2 and Supplementary Section 1.4.

**Table 1:** Food system outcome indicators

Outcome indicator	Unit	Definition	Level of aggregation, timely resolution
‘Underweight’	Million people	Number of adults with a BMI < 18.5 (for people older than 15 years) and children and adolescents with a BMI that is 2 s.d. below normal (0–14 years)	Country level, by age cohorts and sex, for a specific year
‘Obesity’	Million people	Number of adults with a BMI > 30 (for people older than 15 years) and children and adolescents with a BMI that is 2 s.d. above normal (0–14 years)	Country level, by age cohorts and sex, for a specific year
‘Premature mortality’	Million YLL	YLL is a measure of premature mortality that takes into account both the frequency of deaths and the age at which it occurs, using the ‘Global Burden of Disease standard abridged life table’ to represent the standard life expectancy. Definition: one YLL represents the loss of 1 year of life	Country level, by sex, for a specific year
‘Cropland landscapes’	BII	The BII accounts for net changes in the abundance of organisms based on the loss of forest and non-forest vegetation cover and age class of natural vegetation, which are expressed relative to a reference land-use class (forested or non-forested vegetation) and weighted by a spatially explicit range-rarity layer (unitless). The reference land use (BII = 1) is assumed to have no human land use. For the cropland landscape BII, only cells that contain at least 100 ha of cropland are considered.	0.5° × 0.5°, for a specific year
‘Hotspot landscapes’	BII	The BII accounts for net changes in the abundance of organisms based on the loss of forest and non-forest vegetation cover and age class of natural vegetation, which are expressed relative to a reference land-use class (forested or non-forested vegetation) and weighted by a spatially explicit range-rarity layer (unitless). The reference land use (BII = 1) is assumed to have no human land use. For the key conservation landscapes, we considered only cells in biodiversity hotspots and intact forest landscapes.	0.5° × 0.5°, for a specific year
‘Crop area diversity’	Shannon index	The Shannon index is a common metric of crop diversity that takes into account the richness and abundance of different crop groups. The Shannon crop diversity index *H*_crop_ is calculated using the following equation: *H*_crop_ = −∑_*k*_(*p*_*k*_ × log(*p*_*k*_)), where *p* is the share of crop *k* in the total cropland area in each spatial cluster. On the basis of the MAgPIE crop groups, our ‘crop area diversity’ index is based on 13 single crops, 6 crop groups and 1 fallow land group. Within the groups, we assume constant heterogeneity. The crop area diversity index is higher if more crop-area classes exist within a spatial cluster, and if they have more evenly distributed proportions (Supplementary Fig. 20).	0.5° × 0.5°, for a specific year
‘Nitrogen surplus’	Mt N yr^−1^	Nitrogen surplus in croplands, pastures, natural vegetation and animal waste management in Tg N. Nitrogen surpluses in croplands and pastures are the difference between organic and inorganic nutrient inputs and withdrawals by harvests and grazing. In natural vegetations, we assume a stable state in which fixation equals surpluses. In animal waste management, surpluses are total excretion minus recycled manure.	0.5° × 0.5°, for a specific year
‘Environmental water flow violations’	km^3^ yr^−1^	Water withdrawals exceeding the volume that could be withdrawn when taking minimum environmental flow requirements of aquatic and riverine ecosystems into account, in km³.	0.5° × 0.5°, for a specific year
‘GHG emissions’	GtCO_2_e yr^−1^ in AR6 GWP100	GHG emissions from land use and land-use change in GtCO_2e_ using a GWP100 of 273 for N_2_O and 27 for CH_4_ based on AR6	World region level, for a specific year
‘Global surface warming’	°C warming relative to 1951–1980	°C warming of global mean surface air temperature (relative to 1951–1980)	Global, for a specific year
‘Expenditure on agricultural products’	US$ per person per year	Expenditures in USD_05MER_ per capita per year for agricultural commodities dedicated for food use, excluding the value added in the supply chain; estimated as the country-level per-capita food use multiplied by the shadow price (Lagrange multiplier) of providing one additional unit of food in a world region	Country level, for a specific year
‘Poverty’	Million people living below Int$_2011 PPP_ d^−1^	Number of people in millions with a per capita daily income below Int$_2011 PPP_ in each country, based on poverty lines estimated by the World Bank	Country level, for a specific year
‘Agricultural labour demand’	Million people	People working in agriculture (crop and livestock production), in million people	World region level, for a specific year
‘Agricultural wages’	Index relative to 2020	Index describing the development of wages as ratio relative to 2020. Wage is defined as the mean nominal hourly labour cost per employee, including, for example, remuneration for work performed and payment in kind, social security expenditures and welfare services.	Country level, for a specific year Aggregation to global level using constant 2010 country-level population data
‘Bioeconomy supply’	Billion USD_05_ yr^−1^	Value stream from food and land system to other economic sectors, including the value of bioenergy, bioplastics, timber and material use of products at fixed prices of 2010. Food demand is considered internal to the food system.	World region level, for a specific year
‘Production-factor use’	Billion USD_05 _yr^−1^	Use of labour, capital and intermediates in the land system at fixed prices per productive factor unit. Includes their use for agricultural production, primary processing, transport, research and development and food system measures. Excludes land and water use, factor use in secondary and tertiary processing, retail, gastronomy and households, costs in other sectors caused by CrossSector measures, as well as transaction costs for implementing FSMs, for example enforcement and controlling.	World region level, for a specific year

BII, biodiversity intactness index; BMI, body mass index; YLL, years of life lost.

**Table 2:** Description of all food system measures (FSMs) assessed in this study

FSM name	Description
LowProcessed	The intake of sugars is capped at the recommended intake by the planetary health diet^6,8^, while the intake of plant-based oils and fats is converged towards the planetary health diet. Alcohol consumption was limited to a maximum of 1.4% of calorie intake^135^. In the health model, we assume that grains are consumed as whole grains. The consumption of staple foods (cereals, roots, tubers) is adjusted to keep total food calorie intake constant.
HighLegumes	The intake of legumes is increased to the recommendation by the planetary health diet^6,8^ in countries where these values are not already fulfilled. The consumption of staple foods (cereals, roots, tubers) is reduced to keep total food calorie intake constant.
LowMonogastrics	The intake of pig meat, poultry meat and eggs is capped at the recommended intake of the planetary health diet^6,8^. The consumption of staple foods (cereals, roots, tubers) is adjusted to keep total food calorie intake constant.
LowRuminants	The intake of ruminant meat and milk products is capped at the recommended intake of the planetary health diet^6,8^. The consumption of staple foods (cereals, roots, tubers) is adjusted to keep total food calorie intake constant.
HighVegFruitsNuts	The intake of vegetables, fruits, nuts and seeds is increased to levels recommended by the planetary health diet^6,8^. The consumption of staple foods (cereals, roots, tubers) is adjusted to keep total food calorie intake constant.
HalfOverweight	Calorie intake is reduced to achieve a reduction of overweight and obesity by 50% relative to the BASE scenario. Calorie reduction is BMI class, country, age group and sex specific. The intake of half of the people overweight or obese (BMI > 25 for adults, BMI ± 1 s.d. for children) is reduced to the intake recommended for a healthy BMI (20–25, BMI < +1 s.d.). Relative dietary composition is not affected. The intake of people in other BMI classes is not affected.
NoUnderweight	Calorie intake is increased in line with a complete eradication of underweight until 2050 for all age cohorts and sex classes in all countries. Calorie increase is BMI class, country, age group and sex specific. The caloric intake of adults with BMI < 20 and children with BMI < −1 s.d. is increased to the intake recommended for a healthy BMI (20–25, BMI < +1 s.d.). Relative dietary composition is not affected. The intake of people in other BMI classes is not affected.
LowFoodWaste	Food waste in households and retail (difference between calorie intake and FAO food availability) is reduced to a maximum of 20% of intake.
LibTrade	Trade is less oriented along historical trade patterns and more along relative competitiveness. MAgPIE uses two trade pools^136^: The ‘historic trade pool’ is based on historical trade patterns, with importing countries importing a constant share of their domestic demand and exporting countries providing a constant share of global trade. This reflects historical trade distortions and dependencies. The ‘liberal trade pool’ is based on relative cost-competitiveness, in terms of production and trade margins and tariffs. In the LibTrade scenario, the share of the liberal trade pool is increased from 20% to 30% for crops and from 10% to 20% for livestock and secondary products.
MinWage	A global minimum wage increases wages in the lower-income countries. The minimum wage scenario increases wages to at least USD_05MER_3 h^−1^ by 2050. In the model, it raises production costs, causes a labour substitution by capital and increases nominal incomes.
CapitalSubst	In countries with high capital intensity, capital is substituted by labour. We set a global target for the labour:capital share of 80:20. If countries exceed the capital share, we reduce the difference to this target by 50% until 2050. Substituting capital by labour increases ‘agricultural labour demand’ but comes at additional production costs.
REDD+	Deforestation is disincentivized, and regeneration of original vegetation is incentivized through a price on changes in carbon in aboveground vegetation in non-agricultural land. Thereby, it provides incentives for reducing deforestation and for the regeneration of original vegetation^137^. Regeneration such as reforestation uses growth curves and carbon stocks of natural vegetation based on LPJmL. The growth curves are parameterized based on ref. ^138^.
LandConservation	Global land area under protection is doubled from currently ~15% to ~30% by 2030. We assume that the enlargement of protected areas includes both a reactive and proactive component^46,139^. The reactive component focuses on biodiversity hotspots, and the proactive component considers large areas (>500 km^2^) of unprotected intact forest landscapes, mainly in the Amazon and Congo basins and in the boreal zone.
PeatlandRewetting	Drainage of intact peatlands is penalized, and rewetting of drained peatlands is incentivized through the AFOLU GHG price. GHG emissions from drained and rewetted peatlands are estimated based on IPCC wetland GHG emission factors^140^. Drainage of peatlands is linked to the expansion of managed lands (cropland, pasture, forestry). Likewise, rewetting of peatlands is linked to the reduction of managed lands.
WaterConservation	Minimum environmental water flow requirements (following the method of ref. ^141^) have to be maintained and cannot be withdrawn (for irrigation or non-agricultural usage).
BiodivOffset	The BII in each biome of each world region cannot decrease after 2020. BII reduction at one place can be compensated by increasing BII values in other places under the condition that they belong to the same biome in the same world region.
NitrogenEff	Nitrogen uptake efficiency is increased through technical measures such as improved land manure application, spreader maintenance, improved agronomic practices, sub-optimal fertilizer applications, nitrification inhibitors and fertilizer-free zones. We use maximum mitigation rates and the associated costs from ref. ^99^, increasing labour and capital demand based on general regional cost shares in agricultural production. Mitigation rates are translated to changes in soil nitrogen uptake efficiency to improve the consistency with our nitrogen budgets.
CropRotations	Crop rotations are incentivized with payments. Exceeding typical rotation lengths is priced to account for the external costs of less diverse agriculture. For the tax rate of rotation length exceedance, see Supplementary Data 1.
LandscapeHabitats	Permanent habitats are established within agricultural landscapes. Cropland expansion per cluster is constrained to 80% of the available potential cropland. The area of available potential cropland at grid cell level is derived from ref. ^142^. This aims at conserving at least 20% permanent semi-natural habitats at the landscape level (for example, for pollination, pest control, soil protection). Semi-natural habitats include forest, non-forest and grassland habitats that can maintain and restore native species diversity.
RiceMitigation	Technical measures such as direct seeding, improved residue management, alternated flooding and drainage, and changed fertilization; we use the marginal mitigation cost curve by ref. ^99^ to reduce baseline emissions.
LivestockManagement	Livestock systems are intensified in particular in ruminant systems in low-income countries, resulting in a more efficient conversion of feed into products and associated shifts in feed baskets from roughage to concentrate feed^95,96^. In addition, emissions from enteric fermentation are mitigated via the set of technical measures of ref. ^99^ and associated costs.
ManureManagement	Improved animal waste management reduces losses and emissions during collection and storage of manure using a set of measures at additional costs. Around 50% of manure excreted in confinements is managed in anaerobic digesters, while the remainder is still managed according to the current mix. Anaerobic digesters are assumed to have a 90% recycling rate of manure, accounting for some remaining losses in stables and waste collection^57^.
SoilCarbon	Soil carbon degradation is disincentivized, and soil carbon sequestration is incentivized through a carbon price on C in soil carbon (including the litter layer). Disincentivized measures include transition of natural land or pasture to cropland; incentivized measures include irrigation or perennial crops.
Population	Population growth is reduced, in particular in low-income countries, switching population projections^127^ and built-up area projections^143^ from SSP2 to SSP1.
HumanDevelop	Human development is fairer, with higher social justice and better institutions and education; switch from SSP2 to SSP1 projections (1) for per-capita gross domestic product and the Gini coefficient, which implies faster economic growth in particular in low-income regions, and a more equal income distribution between countries and within countries; (2) for non-food-related health risks; (3) for the parametrization of the diet model, choosing a different functional form for the regressions that leads, for example, to a slight decline of animal product demand when income levels become very high (similar to ref. ^144^); (4) for risk premiums on interest rates for long-term investments (for example, irrigation, yield-increasing technological change) due to poor institutions^115^ in low-income regions by 4 percentage points and to a lesser extent in middle-income regions; (5) for the technological progress of the base soil nitrogen uptake efficiency, increasing it by 5 percentage points in all countries with an upper limit of 75% per country; and (6) for physical activity levels, shifting from sedentary to moderate activity, which implies higher food requirements^2^.
EnergyTrans	Sustainable development in energy, industry and transport. Non-AFOLU emissions and bioenergy demand are in line with an energy transformation that stays within a carbon budget of 900 GtCO_2_ (ref. ^27^), non-ag water demand changes from SSP2 to SSP1 (ref. ^145^).
Bioplastics	Of the projected total plastic demand (675 Mt by 2050)^146^, 30% is replaced by bioplastics. Bioplastics require biomaterials as substrates.
TimberCities	Wood is used as construction material in cities. We assume that 50% of new urban dwellers (after 2020) are housed in buildings made of engineered wood^147^ to replace carbon-intensive steel and concrete housing construction. This increases future timber demand by 2212 million m^3^ (compared with 2020) and thereby increases the need for increased harvesting from forests.

Further details can be found in Supplementary Section 1.4.

Our study expands on previous quantitative assessments^8,25–32^ by extending the set of analysed FSMs and outcome indicators, in particular on social inclusion. Our study innovates by conducting a multi-measure, multi-criteria assessment, covering 23 FSMs and 5 CrossSector transformations and evaluating their impact on 15 indicators within a single, consistent, quantitative framework. Simulating the effects of FSMs individually and in packages highlights specific synergies and trade-offs.

**Fig. 1:**
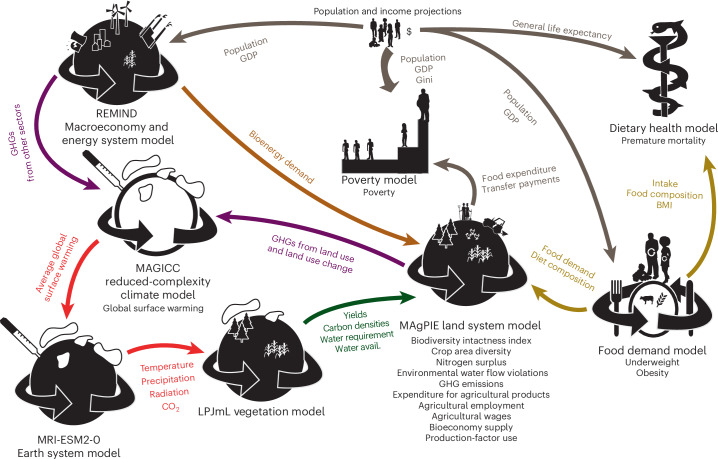
Illustration of the Potsdam Integrated Assessment Modelling (PIAM) framework and further models integrated for this study. The arrows indicate the parameters exchanged between models; black font indicates model names as well as the core outcome indicators each model estimates. Note that information between soft-linked PIAM models is not necessarily exchanged during run time but is taken from consistent offline simulations. Figure adapted from ref. ^148^ under a Creative Commons license CC BY 3.0.

This integrated assessment is carried out using the open-source land and food system model, the Model of Agricultural Production and its Impact on the Environment (MAgPIE)^20^, linked with a food demand model^2^; vegetation, crop and the hydrology model Lund–Potsdam–Jena model with managed land (LPJmL)^33,34^; the reduced-complexity climate model MAGICC (Model for the Assessment of Greenhouse Gas Induced Climate Change)^35^; a dietary health model^8,36^; and an income distribution and poverty model^37^, as well as with results from the macro-economy and energy system model REMIND (Regional Model of Investment and Development^)38^ and the Earth System Model MRI-ESM2 (Meteorological Research Institute Earth System Model version 2)^39^ (Fig. 1). We simulate the impact of each individual measure, as well as their interaction, in packages focusing on five policy fields: (1) ‘Diets’, (2) ‘Livelihoods’, (3) ‘Biosphere’, (4) ‘Agriculture’ and (5) ‘CrossSector’ impacts from transformations outside the food system. Our approach focuses on the primary production of crop and livestock commodities as well as final food consumption, but does not cover measures targeting food environments or supply chains, such as food processing, marketing or disposal^40^. Furthermore, the policy instruments and the political economy necessary to implement the measures^24^ are not within the scope of this analysis.

## Results

Our assessment up to 2050 shows that each individual FSM creates trade-offs, but the packaging of FSMs can enhance co-benefits and reduce trade-offs. Combining all measures within the food system achieves a comprehensive improvement of most outcomes relative to the reference scenario, but it also requires cross-sector transformations to create a sustainable food system that aligns with the 1.5 °C climate target. The quantitative and consistent integration achieved by our comprehensive multi-criteria analysis fills a gap in the literature, which usually looks at a much more limited set of measures and outcomes using non-harmonized approaches^41^, and will provide a key input for upcoming regional and global assessments such as the IPCC and IPBES.

### Food security improves, but dietary health deteriorates in the reference scenario

Over the period 2020–2050, our BASE_SSP2_ reference scenario (Supplementary Section 1.3) projects population growth from 7.8 to 9.4 billion people, reduced food insecurity but declining dietary health, general deterioration of the environment, declining absolute poverty and falling ‘agricultural labour demand’.

**Fig. 2:**
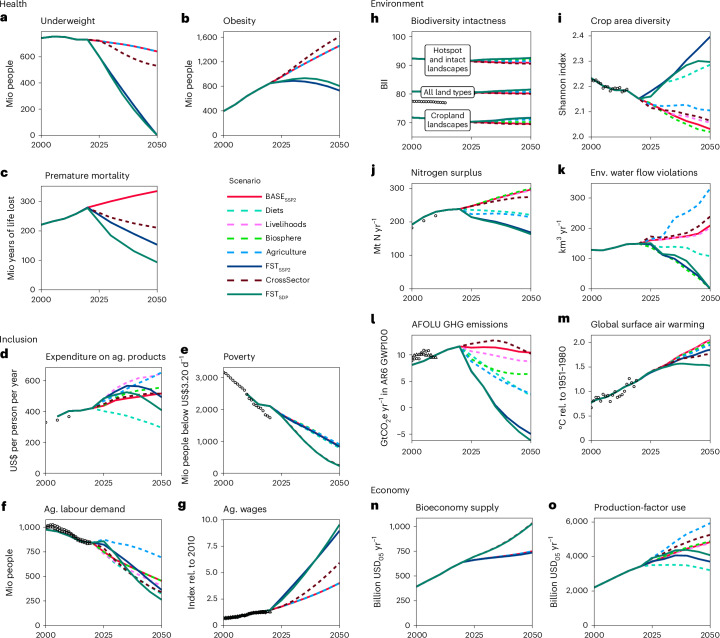
Scenarios for 15 food system outcome indicators. **a**–**o**, Scenarios for the 15 food system outcome indicators: underweight (**a**), obesity (**b**), premature mortality (**c**), expenditure on agricultural products (**d**), poverty (**e**), agricultural labor demand (**f**), agricultural wages (**g**); biodiversity intactness of all land types, of cropland landscapes and of hotspot landscapes (**h**); croparea diversity (**i**), nitrogen surplus (**j**), environmental water flow violations (**k**); greenhouse gases from agriculture, forestry and other land use (**l**); global surface air warming (**m**), bioeconomy supply (**n**) and production-factor use (**o**). BASE_SSP2_ (red line) describes a middle-of-the-road scenario. FST_SSP2_ (blue line) describes a food system transformation pathway that combines four packages of FSMs targeting healthy diets (Diets), livelihoods (Livelihoods), biosphere integrity (Biosphere) and agricultural management (Agriculture). If the cross-sectoral impacts of sustainable transformations in other parts of the economy (CrossSector) are added, we arrive at a food system transformation pathway in the context of a sustainable development pathway (FST_SDP_, green line). All outcome indicators are described in Table 1; historical data points (dots) are described in Supplementary Section 2.1. mio, million; ag., agricultural; rel., relative; env, environmental.

**Extended Data Fig. 1:**
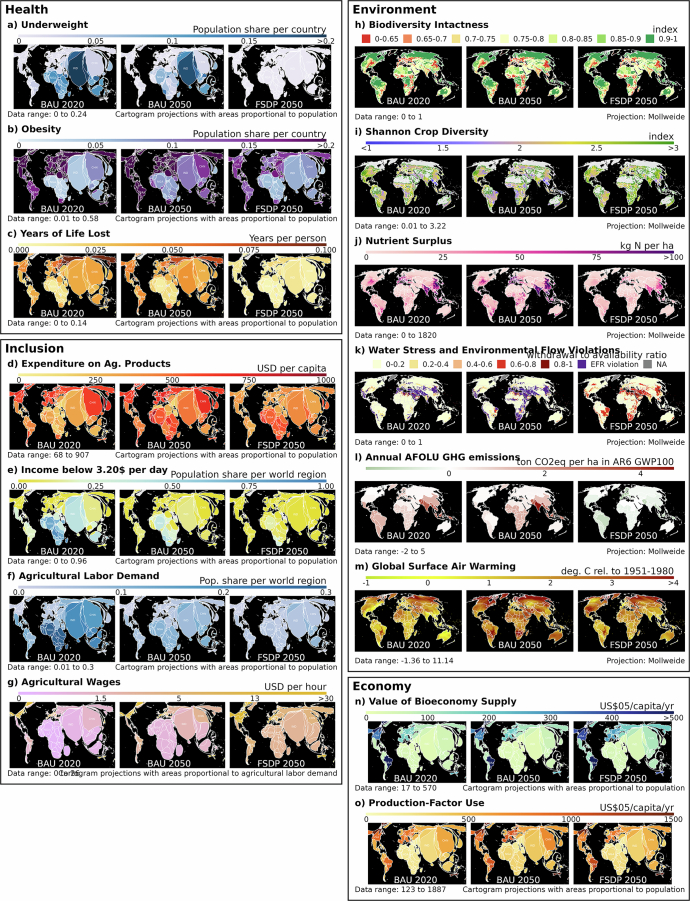
Spatial patterns of the 15 food system outcome indicators - grouped by health, inclusion, environment and economy - for the reference *BASE*_*SSP2*_ scenario in 2020 and 2050, and the food system transformation pathway FST_SDP_ in 2050. Spatial patterns of the 15 food system outcome indicators - grouped by health, inclusion, environment and economy - for the reference BASE_SSP2 scenario in 2020 and 2050, and the food system transformation pathway FST_SDP in 2050: (**a**) underweight, (**b**) obesity, (**c**) premature mortality, (**d**) expenditure on agricultural products, (**e**) poverty, (**f**) agricultural labor demand, (**g**) agricultural wages, (**h**) biodiversity intactness, (**i**) croparea diversity, (**j**) nitrogen surplus, (**k**) environmental water flow violations, (**l**) greenhouse gases from agriculture, forestry, and other land use, (**m**) global surface air warming, (**n**) bioeconomy supply, (**o**) production-factor use. Spatial resolution differs by indicator (Table 1), and we use a Mollweide projection (area-preserving projection) for environmental indicators, and a Cartogram projection (areas proportional to population or agricultural workers) for health, inclusion and economy indicators.

Diet-related health follows divergent trends (Fig. 2a–c). First, the number of people ‘underweight’ falls from 730 to 640 million, with the highest remaining prevalence in South Asia and falling prevalence in Africa (Extended Data Fig. 1a). These projections do not consider food security impacts from increases in violent conflicts, pandemics or natural disasters. Second, in line with the nutrition transition towards energy-dense and nutrient-poor diets^2^, the number of people affected by obesity increases from 848 to 1,461 million, a conservative estimate compared with that of ref. ^42^. In 2050, obesity is most prevalent in current high-income regions (HIRs), with high levels also found in emerging economies in Latin America, East Asia and West Africa (Extended Data Fig. 1b). Southern Africa and Southeast Asia suffer the double burden of malnutrition, with high levels of under- and overnutrition. We find an increase in ‘premature mortality’ due to dietary and metabolic health risks from 279 to 335 million life years lost per year. The highest rates of diet-related premature mortality are in the Global North, particularly in eastern Europe, and the lowest are in East, West and Central Africa (Extended Data Fig. 1c), a geographical pattern mirroring the international consumption patterns of healthy and unhealthy food items^43^.

### Most global environmental indicators deteriorate in the reference scenario

Environmental indicators generally deteriorate in the baseline scenario (Fig. 2h–m and Extended Data Fig. 1h–m).

Aggregating biodiversity change to the global scale remains a conceptual challenge owing to the diversity of associated values^44^. Here we use a set of different outcome indicators to capture the key drivers of biodiversity change related to the food system (Table 1). The impacts of land-use change on biodiversity are mapped spatially using the ‘biodiversity intactness’ index (BII)^45^. Global land-use dynamics cause a continued decline of BII values. Despite strong increases in crop yields (Supplementary Fig. 34), expansion of agriculture particularly affects the BII in biodiversity ‘hotspot and intact forest landscapes’, which are critical to global biodiversity conservation^46^. Loss of landscape heterogeneity also drives BII decline in ‘cropland landscapes’, implying a continued loss of biodiversity and critical ecosystem functions in cultured landscapes^47^. ‘Crop area diversity’ also declines, attributable to specializing in high-yielding crops with shorter rotations. The decline is most pronounced in Latin America, Europe and the USA. Further drivers of biodiversity change are captured by our indicators for nutrient pollution, climate change and unsustainable water use.

‘Nitrogen surplus’ is a key indicator of the impacts of nitrogen pollution on air, water, soils and the atmosphere^48^, causing harm to biodiversity, global health and the economy^49^. We here define ‘nitrogen surplus’ as the sum of nitrogen lost from croplands, pastures, animal waste management and natural vegetation. Our model estimates an increase from 239 Tg N yr^−1^ to 297 Tg N yr^−1^ over the period 2020–2050. Pollution hotspots are found in China, India, eastern Europe, the Corn Belt in North America and the Plata Basin in South America. Nitrogen pollution intensifies towards 2050, particularly in India. Moderate pollution levels are also reached in sub-Saharan Africa as its agricultural sector grows and intensifies.

‘GHG emissions’ from agriculture, forestry and other land use (AFOLU) fall slightly from 11.6 Gt CO_2_ equivalents (GtCO_2_e; using AR6 100-year global warming potential (GWP100)) in 2020 to 10.4 GtCO_2_e in 2050 (see Supplementary Fig. 15 for individual N_2_O, CH_4_ and CO_2_ emissions). Emissions are highest in densely populated areas, particularly in Asia, while reforestation and afforestation compensate for many of the agricultural emissions in the Global North (Supplementary Fig. 26). Combined with emissions from outside the land-use sector, these emissions from AFOLU induce a ‘global surface warming’ of 2.05 °C in 2050 compared with the reference period of 1850–1900, most closely aligned with the Representative Concentration Pathway 6.0 (RCP 6.0; Supplementary Fig. 25). Thus, despite integrating a list of national policies implemented and nationally determined contributions (Supplementary Section 1.3), our reference scenario probably violates the temperature target of the Paris Accord even before 2050. The global climate model MRI-ESM2 used for this study shows the highest temperature increases in North America, North and East Asia, and Australia.

The globally aggregated volume of ‘Environmental Water Flow Violations’^50^ due to excessive water withdrawals by agriculture and other industries increases in the reference scenario. In 2020, environmental flow violations are estimated to occur in the Middle East, Mediterranean and South and East Asia as well as the West Coast of the USA. By 2050, southern and eastern Africa as well as eastern South America also show environmental flow violations (Extended Data Fig. 1k).

### Poverty is reduced, agricultural labour demand strongly declines and the share of agriculture in the global economy falls in the reference scenario

On social inclusion (Fig. 2d–g and Extended Data Fig. 1d–g), the reference scenario projects a reduction of the global ‘poverty’ headcount of people living on less than Int$_2011PPP_3.20 per day (international dollars of the benchmark year 2011 converted on purchasing power parity) from 2,104 to 852 million people in 2050, largely due to economic growth in low-income countries. ‘Expenditure on agricultural products’—estimated as the annual value of agricultural commodities that are used for food, but excluding the substantial value added in processing and marketing^40,51^—increases from USD_05MER_421–517 per capita (US$ of the benchmark year 2005 converted using international market exchange rates) owing to more diverse and resource-intensive diets, with the strongest increases in sub-Saharan Africa and South Asia. Our model projects improvements in agricultural labour productivity and ‘wages’ (in the model represented by the mean nominal hourly labour cost per worker), with a concurrent decline in agricultural labour demand from 843 to 454 million people by 2050, most strikingly in sub-Saharan Africa and Asia. The desirability of this outcome is unclear. Higher labour productivity resulting in lower ‘agricultural labour demand’ and higher ‘agricultural wages’ can be seen as a coherent and necessary outcome of economic development^52^. On the other hand, the speed of structural change may exceed the adaptive capacity of individuals as well as social and political institutions^53,54^ through processes such as unemployment or migration that we do not reflect in our modelling framework yet. The indicator ‘agricultural labour demand’ therefore highlights not only the challenge but also the opportunity to integrate agricultural labour into the service and industry sectors.

The food system is embedded in the wider economy (Fig. 1n,o), delivering non-food materials as ‘bioeconomy supply’ and requiring the use of labour, capital and intermediates, which we report as ‘production-factor use’, aggregated using constant prices per productive factor unit. Higher bioeconomy supply and lower ‘production-factor use’ point at a higher production potential of the other economic sectors. The ‘bioeconomy supply’ for the purpose of non-food materials and energetic use increases slightly, and is located mostly in the Global North and Brazil. ‘Production-factor use’ grows from 3.4 trillion to 4.8 trillion. Still, it declines as a share of total gross world product (Supplementary Fig. 29) as other sectors grow faster than agriculture in line with structural change in the past.

### Other baseline scenarios are not in line with sustainable development either

**Fig. 3:**
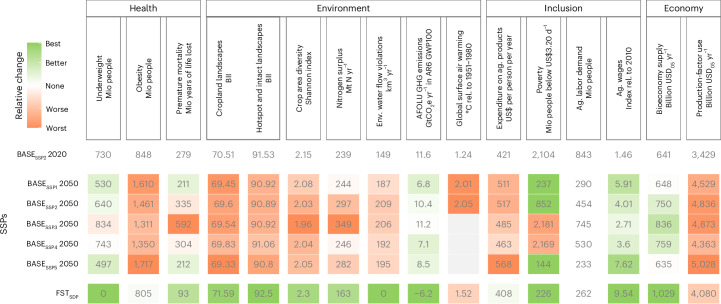
Projections for the five baseline scenarios of the shared socio-economic pathways (BASE_SSPs_) and for the food-system sustainable-development pathway (FST_SDP_) in 2050 relative to the state in the year 2020. The colours indicate desirable developments since 2020 in green and undesirable developments in red. ‘Agricultural labour demand’ received no colour coding, as the changes in ‘agricultural wages’ are the more meaningful welfare indicator in BASE scenarios.

Besides the BASE_SSP2_ reference scenario, we also simulate the other four SSP baseline scenarios (Fig. 3), which diverge with respect to the central socio-economic assumptions (Supplementary Section 1.3). Despite modest improvements with respect to individual outcomes, our study provides supporting evidence that baseline trends will not improve food security sufficiently^31^, nor obesity^42^, health^36^, biodiversity^29^, nitrogen pollution^55^, water^8^, GHG emissions^7^ or ‘agricultural labour demand’^52^. Only with respect to poverty and agricultural wages do two of the five baseline scenarios (BASE_SSP1_ and BASE_SSP5_) show substantial improvements as a consequence of income growth and reduced inequalities^37^. Even though none of our scenarios includes explicit crisis events such as pandemics or wars, the outcomes of all baseline scenarios are largely undesirable and unsustainable. While economic growth is one of the central drivers of the different trajectories, major reductions in economic growth do not substantially reduce environmental impacts^21^, and slows down progress towards a reduction of hunger and poverty^2,37^. A more fundamental and effective transformation of the food system is necessary.

### Each of the FSMs generates co-benefits and trade-offs

**Fig. 4:**
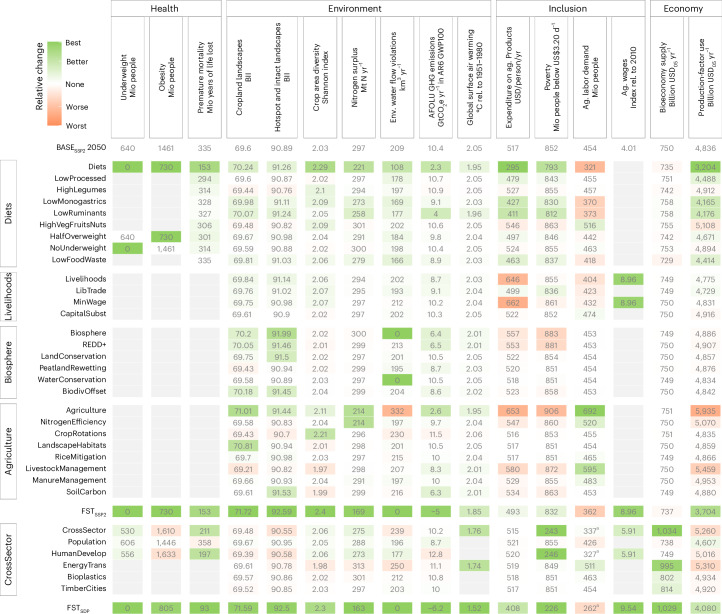
Impact of food system measures (FSMs) on key outcome indicators. The food system transformation (FST) scenarios combine the ‘Diets’, ‘Livelihoods’, ‘Biosphere’ and ‘Agriculture’ packages, once in the context of the SSP2 scenario (FST_SSP2_) and once in the context of a sustainable development pathway (FST_SDP_), which includes CrossSector impacts from measures outside the food system. Green fields indicate an improvement compared with the reference BASE_SSP2_ in 2050; red colours indicate a deterioration compared with the reference. Grey fields have not been quantified. All indicators refer to the state in 2050. A description of the outcome indicators and FSMs can be found in Tables 1 and 2. ^a^The HumanDevelop scenario shows an increase of ‘agricultural wages’ in line with the improved economic development. The decline in ‘agricultural labour demand’ can therefore be evaluated as neutral (white colour), as the ‘agricultural wages’ are the more welfare-relevant indicator and capture this desirable development. Agricultural labour demand in the FST_SDP_ pathway and the CrossSector scenario include the HumanDevelop measure and are therefore evaluated relative to HumanDevelop.

**Extended Data Fig. 2:**
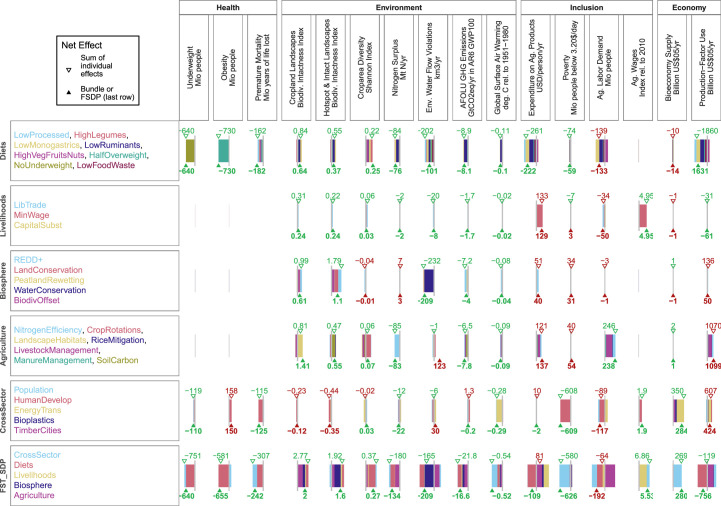
Interaction effect of combining measures in packages. Bars show the individual outcomes of the measures. The upper number with the empty triangle shows the sum of the individual effects of measures. The lower number with the solid triangle shows the combined effect of a package. Green numbers are co-benefits, red numbers are trade-offs. Synergies occur when the combined effect is positive and larger than the sum of the individual effects, dampening when it is smaller than the sum.

To improve health, social inclusion, environment, and economic outcomes, we investigate the impact of 23 discrete FSMs, described in Table 2. We find that all individual FSMs come with co-benefits and trade-offs across outcome indicators (Fig. 4). When the FSMs are combined as packages along the major policy fields, the outcome profiles of the individual measures overlap, often enhancing co-benefits and reducing trade-offs. Packaging FSMs together can develop interaction effects that further reinforce or dampen the combined impact relative to the sum of individual impacts (Extended Data Fig. 2).

The ‘Diets’ package develops generally positive synergies across 12 of 15 indicators. The strongest co-benefits stem from FSMs that reduce the resource intensity of diets by reducing waste (LowFoodWaste) or replacing animal products by plant-based staple crops, which strongly attenuates resource requirements from feed production (LowMonogastrics, LowRuminants, HalfOverweight FSMs). They do, however, reduce ‘agricultural labour demand’, which is particularly high in livestock and feed production. By contrast, dietary shifts that scale up consumption of healthy food items (HighLegumes, HighVegFruitsNuts, NoUnderweight FSMs) increase ‘agricultural labour demand’ but show modest trade-offs with some environmental indicators as well as expenditure on agricultural products. Overall, the package creates co-benefits with respect to health, environment and poverty, with a trade-off in the case of reduced labour demand in agriculture.

The ‘Livelihoods’ package improves eight indicators, with major trade-offs for expenditure on ‘agricultural products’ and ‘agricultural labour demand’. The LibTrade FSM, shifting from historical trade patterns to more open trade, allows more efficient allocation of water, land and fertilizer. It thereby leads to modest environmental improvements. Minimum wages (MinWage FSM) improve livelihoods, but also drive up expenditure on agricultural products, and lead in our model to a decrease in agricultural labour demand owing to intra- and inter-regional capital–labour substitution (without accounting for demand-increasing income redistribution effects, Supplementary Section 1.4.2). These job losses are reinforced when trade is opened at the same time owing to production displacement to more capital-intensive world regions (Extended Data Fig. 2).

The ‘Biosphere’ package generates more heterogeneous outcomes. Strong benefits occur for BII, ‘environmental water flow violations’ and ‘GHG emissions’, as water and land resources are protected by these FSMs. The need to intensify production on remaining agricultural land in the absence of demand-side reductions leads, however, to modest trade-offs in the form of shorter crop rotations with ‘lower crop area diversity’, and also higher ‘nitrogen surplus’. Moreover, ‘expenditure on agricultural products’ rises, as do poverty rates, because of increasing costs of agricultural production and in turn of food products.

In the ‘Agriculture’ package, the individual FSMs often have complementary environmental benefits and trade-offs. The FSM LivestockManagement substantially mitigates ‘GHG emissions’, but also results in a shift from roughage to concentrate feed. As a consequence, croplands become more agglomerated and have shorter crop rotations. These effects are offset by the Landscape Habitats and CropRotations FSMs, respectively, which in turn add further pressure for cropland expansion. By contrast, the SoilCarbon FSM provides large incentives to stop converting new land, as this would deplete soils. If the ‘Agriculture’ FSMs are packaged, the FSMs reduce each others’ trade-offs, but expanding irrigation remains as a viable option, which is why ‘environmental water flow violations’ of the ‘Agriculture’ package is considerably higher than the sum of the additional violations of individual FSMs (Extended Data Fig. 2). Generally, trade-offs are seen with respect to ‘production-factor use’, ‘expenditure on agricultural products’ and ‘poverty’. Instead, ‘agricultural labour demand’ increases strongly, mainly owing to the shift from roughage to cropland production, but also for implementing the mitigation measures in the NitrogenEff and ManureManagement FSMs.

Trade-offs can be reduced if measures are packaged together with all 23 FSMs; the FST_SSP2_ pathway improves outcomes for 13 of 15 indicators compared with BASE_SSP2_ (Figs. 2 and 3). The integration across and within policy fields reveals clear complementarities (Extended Data Fig. 2): while the ‘Diet’ and ‘Livelihoods’ packages reduce ‘agricultural labour demand’, mitigation activities within the ‘Agriculture’ package increase it. Production costs are increased by the ‘Agriculture’ package, but decreased by the ‘Diet’ package. Water stress that is increased by the ‘Agriculture’ package is mitigated as environmental water flows are protected by the ‘Biosphere’ package. Finally, the lower ‘crop area diversity’ and the intensified ‘nitrogen surpluses’ induced by the ‘Biosphere’ package are counterbalanced by resource-efficient management and longer crop rotations in the ‘Agriculture’ package, as well as reduced demand pressure in the ‘Diet’ package.

**Extended Data Fig. 3:**
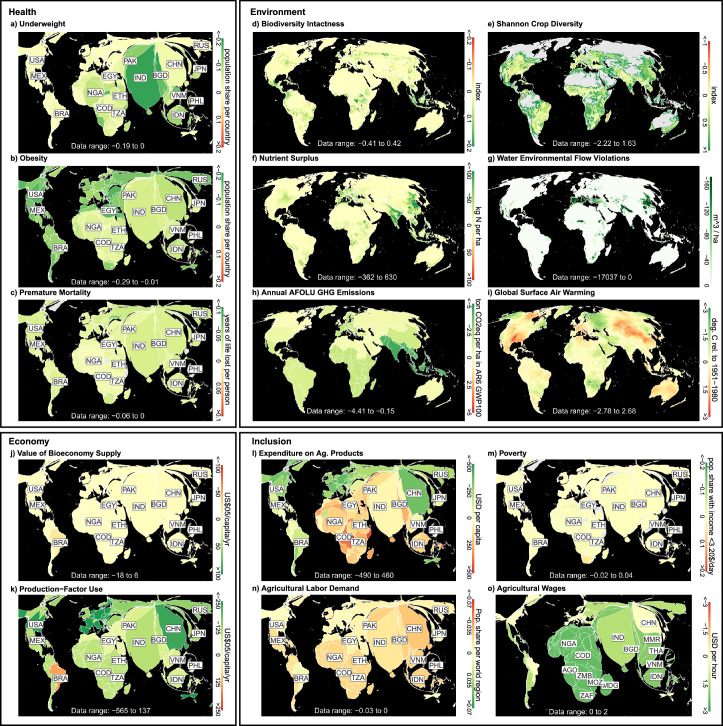
The combined effect of 23 food system measures on 15 outcome indicators. The combined effect of 23 food system measures on 15 outcome indicators: (**a**) underweight, (**b**) obesity, (**c**) premature mortality, (**d**) biodiversity intactness, (**e**) croparea diversity, (**f**) nitrogen surplus, (**g**) environmental water flow violations, (**h**) greenhouse gases from agriculture, forestry, and other land use, (**i**) global surface air warming, (**j**) bioeconomy supply, (**k**) production-factor use, (**l**) expenditure on agricultural products, (**m**) poverty, (**n**) agricultural labor demand, (**o**) agricultural wages. Results are shown as the difference between *FST*_*SSP2*_ and *BASE*_*SSP2*_ scenarios, not including the *CrossSector* transformation impacts. The food system transformation has co-benefits (green) and trade-offs (red). Spatial resolution differs by indicator (Table 1), and we use a Mollweide projection (area-preserving projection, per-ha values are per total cell area) for environmental indicators, and a Cartogram projection (areas proportional to population or agricultural workers) for health, inclusion and economy indicators.

Health and environmental indicators, both globally and regionally (Extended Data Fig. 3), mostly improve compared with BASE_SSP2_. When comparing the FST_SSP2_ pathway to the world’s current state in 2020 (BASE_SSP2_ 2020 in Fig. 3), only global warming worsens. Accounting for the uncertainties in the climate system’s response to present and future emissions^35^, the FST_SSP2_ pathway will be 1.85 °C (median estimate) warmer in 2050 than the 1850–1900 reference period, with a 96% probability that the 1.5 °C target will be exceeded by 2100 and a 60% probability that the 2 °C target is transgressed, in contrast to a 95% probability in BASE_SSP2_ (Supplementary Fig. 27). This shows the large contribution FST can make to climate mitigation, even in the absence of an energy transition; in the FST_SSP2_, AFOLU ‘GHG emissions’ turn net-negative by 2035 as forests sequester carbon.

Despite the high costs of mitigation and increased consumption in previously food-insecure countries, the FST_SSP2_ pathway does not increase absolute global poverty and even leads to a modest reduction compared with BASE_SSP2_ in 2050. Reduced food consumption in richer countries together with trade liberalization decreases food prices and dampens the increase in ‘expenditure on agricultural products’. Simultaneously, higher wages coupled with the recycling of revenues from CO_2_ taxation increase real incomes and reduce poverty in many low-income countries. Yet, poverty remains nearly as widespread a phenomenon in the FST_SSP2_ as in the reference scenario. Although new employment opportunities arise from mitigation activities in agriculture, it is not enough to compensate for the reduced labour demand attributable to less resource-intensive diets. Thus, ‘agricultural labour demand’ is 92 million people lower in FST_SSP2_ than in BASE_SSP2 in 2050_.

### The transformation of the food system must be embedded in a broader sustainability transformation

Our investigation of the cross-sector impact of five sustainability transformations outside the food system (CrossSector package, Fig. 4) indicates that the FST must be embedded within an economy-wide sustainable development pathway^27^ to halt global warming, reduce absolute poverty, ease structural change and achieve further sustainable development goals. Slower population growth stemming from improved socio-economic development (Population), with only 8.9 billion people by 2050, reduces pressure in agricultural markets and environmental degradation. More sustainable human development, including faster and more equitable economic growth in low-income countries (HumanDevelop), reduces undernutrition and poverty, but increases ‘obesity’ and expenditure on ‘agricultural products’. Due to increasing labour productivity and higher deployment of capital, ‘agricultural labour demand’ in HumanDevelop is even lower than in BASE_SSP2_. The additional labour demand for the provision of agricultural materials for the bioeconomy (EnergyTrans and Bioplastics scenarios) does not create sufficient alternative employment within agriculture to compensate for this. However, as the global wage index is 47% higher in the CrossSector package than in the reference scenario BASE_SSP2_, the remaining jobs provide better livelihoods. The sustainable transformation of the energy system (EnergyTrans) reduces global warming to 1.74 °C by 2050, with only a 28% chance of exceeding 2 °C in 2100, while the demand for second-generation bioenergy remains low before the second half of the century and therefore has small impacts on the food system up to 2050 in our model assessment.

### A sustainable food system requires transformation at massive scale and speed

The FST in the context of an economy-wide sustainable development pathway (FST_SDP_ = FST_SSP2_ ∪ CrossSector) illustrates the possibility and quantitative consistency of a global food system that nourishes a healthy population, provides affordable food with a low environmental footprint and improves livelihoods in agriculture. The FST_SDP_ simultaneously improves 14 out of 15 key outcome indicators. It aligns with SDG2 in ending hunger and the World Health Organization target to halt the rise of obesity^4^. Mortality is reduced by 242 million life years per year by 2050, the degradation of the biosphere is halted and the pressure on biodiversity is reduced compared with today. In line with target 2 of the Kunming–Montreal Global Biodiversity Framework^26^, almost 30% of the global land area is under conservation by 2030. According to the quantified climate model uncertainty, the emission trajectory of FST_SDP_ keeps global warming below 1.5 °C with 38% probability and below 2 °C with 91% probability by 2050, with peak warming occurring before 2040 (Supplementary Fig. 27). Reductions in emissions of the short-term forcer methane by LowRuminants and LivestockManagement are particularly important in achieving the 1.5 °C target^56^.

In line with previous studies^8,27,28,57^, we find that it is very challenging to meet the planetary boundary for nitrogen pollution. Nitrogen surpluses from agricultural soils (here excluding surplus from manure management and natural soils for comparison purposes with ref. ^48^) are reduced drastically from 190 Mt N in the reference to 64 Mt N in the FST_SDP_ pathway. But these still exceed the planetary threshold of 57 Mt N (ref. ^48^) as well as critical regional thresholds, in particular in hotspots such as China and India. Also, by 2030, the anthropogenic ‘nitrogen surplus’ is only reduced by 22% (Supplementary Table 1) and not halved as agreed by the Kunming-Montreal Global Biodiversity Framework 2030 target 7 (ref. ^26^).

The agricultural labour force is much better paid, and more equitable human development outside the food system ensures that the absolute poverty headcount falls by 626 million to 226 million. Despite considerable mitigation costs in agricultural production, plant-based consumption patterns coupled with less food waste reduce ‘production-factor use’ globally in FST_SSP2_ and FST_SDP_.

Comparing FST_SDP_ and FST_SSP2_ shows that a sustainable food system requires transformations in the rest of the economy, including most importantly reducing GHG emissions in the energy sector, reducing poverty to make healthy food affordable for all, increasing demand for bio-based materials and fuels, and absorbing the excess agricultural labour force caused by structural changes in the food system.

The FST_SDP_ pathway further highlights that these changes must occur rapidly to achieve a sustainable food system by 2050 (Fig. 2). This quantitative pathway helps to identify the necessary changes and break them down into concrete measures and intermediate milestones, which can then serve to benchmark progress in different parts of the food system (Supplementary Table 1). Milestones for 2030 include a decrease in animal-based product intake of 31% in current HIRs and 10% in middle-income regions (MIRs). The production of fruits, vegetables and nuts in contrast has to be scaled up by 29% globally. Cereal yields need to increase in low-income regions (LIR) by 35%, in MIRs by 15% and in HIRs by 4% by 2030. Net-zero land use change emissions should be reached before 2030, while total AFOLU emissions should be net-zero before 2040. By 2040, 31 Mha of drained peatlands should be rewetted. Forest plantation areas should increase from 149 Mha to 222 Mha by 2040. Global soil nitrogen uptake efficiency should rise from 58% in 2020 to 70% by 2040. To accommodate structural change, the social systems and job markets in industry and services need to absorb 396 million people by 2040 that formerly worked in agriculture, predominantly in LIRs and MIRs.

## Discussion

This study presents a comprehensive assessment of an FST pathway, distinguished by its use of a multi-measure, multi-indicator matrix. Nevertheless, limitations remain regarding the breadth of the outcome indicators, the measures included and the modelled processes that connect them.

For instance, a number of welfare-relevant outcomes go beyond the scope of our study, such as the indirect health impacts of agricultural air pollution, the environmental impacts of phosphorus and pesticides, emissions and factor use in the food supply chain from agriculture to households, transaction costs of enforcement measures, the costs of cross-sectoral measures outside the food system and consumer welfare losses. Consumer welfare losses from dietary change would be very high if preferences and food environments remain unchanged^58^, while they could be minimal or conceptually difficult to estimate if they come from intrinsic preference change^59^.

While we explicitly consider a broad range of FSMs—across the domains of dietary change, livelihoods, biosphere conservation and agricultural management, as well as cross-sectoral measures—the real option space remains much larger. For example, we do not examine the disruptive potential of emerging technologies such as novel foods, digital agriculture, agroecology and robotics^60^. If scaled, these could accelerate transformation at potentially lower costs but may also introduce new adverse side effects^61^.

The option space includes not only further measures but also varying strength (for example, different degrees of trade liberalization), design (for example, different conservation priority areas) and combinations of existing measures. The subjective choices made by the authors do not aspire to fulfil criteria of welfare optimality, which would in any case depend on an inherently subjective welfare function. Yet, by presenting both benefits and trade-offs of each measure and their combinations, we enable readers to assess outcomes based on their own priorities. We acknowledge that, for many scenario settings, there is a continuum of possibilities with continuous implications. Usually, stronger assumptions amplify both co-benefits and trade-offs while reducing political feasibility, but no clear focal point for setting the scenario parameter exists. A more exhaustive exploration of the option space could be achieved by using meta-models such as those of ref. ^62^, building upon the results of studies such as ours.

Supplementary Section 1.4 and Supplementary Data 1 document the major processes within our model framework that connect the 23 FSMs and the 5 CrossSector transformations with the 15 outcome indicators and discuss further important processes that exist in reality but are not captured by our modelling. For instance, we do not cover potential yield improvements from higher soil carbon sequestration in our SoilCarbon FSM. Climate change could reduce labour productivity and labour supply per worker in agriculture, which is an outdoor activity with high physical activity particularly prone to hot and humid climates^63,64^. Mitigation could preserve labour productivity and result in lower employment and higher farm incomes. Limitations also include the fact that our food demand system is price inelastic and therefore neglects upstream effects of supply-side measures.

A further limitation is that MAgPIE is a sectoral model that assumes that the supply of inputs such as labour, capital or inorganic fertilizer is perfectly price elastic. This assumption can be a strong limitation in regions where agriculture is still a major sector of the economy, for example, in sub-Saharan Africa, and in scenarios in which the magnitude of the change in agricultural labour demand is large and happens over a short time span, like in our dietary change scenarios. Taking the example of the labour market in our ‘Diet’ package, the sudden and strong drop of labour demand should depress wages, at least temporarily. This would also lower food prices and agricultural expenditures.

Moreover, all interventions result in distributional effects that vary across population groups depending on their ownership of land, labour and capital, which is not reflected within our poverty model. While trade liberalization often has net poverty-reducing effects like in our study, the distributional impacts of income shifts across population groups and sectors are often more dominant than the distributional impacts via consumption prices that we simulate^65^. Similarly, the FSM MinWage, which introduces a global absolute minimum wage rate for agricultural labour, raises expenditures for agricultural products, while the additional income from the minimum wage is recycled distribution-neutral to the population. In reality, such an intervention would probably benefit the rural poor while the urban poor could be worse off as their wage levels are not affected, while their food expenditures rise.

Our quantitative results generally corroborate the evidence found in the qualitative multi-criteria synthesis of the IPCC^66,67^ (AR6WG3 Fig. 17.1., SR15 section 5.4.1.3), in particular with regard to the broad co-benefits of dietary change. However, we newly quantify a potential trade-off with ‘agricultural labour demand’. While the IPCC^66^ finds strong environmental co-benefits of methane (CH_4_) and nitrous oxide (N_2_O) mitigation as well as carbon sequestration, we find more heterogeneous outcomes of agriculture FSMs with both environmental trade-offs and co-benefits. Furthermore, we quantify major novel ‘agricultural labour demand’ opportunities in mitigation activities in agriculture, and quantify their undesirable trade-offs with poverty. The IPCC^66^ finds clear benefits of biosphere protection and restoration for life on land and water. While our ‘Biosphere’ package also finds an improvement in ‘biodiversity intactness’ and safeguards environmental water flows, we also observe negative environmental outcomes for ‘crop area diversity’ and ‘nitrogen surpluses’ owing to the intensification of the remaining agricultural land.

The susceptibility of the global food system to crises became strikingly visible through recent disruptions: the COVID-19 pandemic; violent conflicts in Ukraine, Gaza, Syria, Yemen, Sudan and Myanmar; natural disasters including the Horn of Africa drought and Pakistan’s 2022 floods; and escalating trade tensions from steep US tariff increases. These crises exacerbate food insecurity and poverty via asset loss, inflation, economic downturns, reduced coping strategies and increased comorbidity^68,69^, contributing to the unexpected rise of food insecurity in the period 2017–2022^70^. They also create a bias towards short-term crisis response at the expense of long-term sustainability investments that could prevent future crises^71^. While our scenarios do not include such crisis events in future projections, our FST pathway has clear implications for future food system risks, which can be structured into changes in hazards, vulnerabilities and adaptive capacities^72^.

Natural hazards are largely reduced in the FST_SDP_ through effective climate change mitigation, preventing floods and droughts^73^, and by reducing livestock numbers and avoiding disturbance to semi-natural ecosystems, which lowers the hazard of food-borne epidemics^74^. Vulnerabilities are reduced by addressing issues such as undernutrition, obesity and chronic diseases^6^, and by reducing inequality and poverty^75^. Also the projected diversification of production and diets can reduce vulnerabilities^76^. Adaptive capacities are strengthened by safeguarding natural resources and conserving their ecosystem services. Adaptive capacities are particularly dependent on sustainable development beyond the food system, including, for instance, improvements in the rule of law and reductions in conflict^27^.

If FSMs are implemented in isolation, economic hazards such as food price inflation^77^ and vulnerabilities such as dependencies on international transfers and trade^37,76^ can be amplified. FSMs require other FSMs to mitigate adverse side effects. For instance, high biofuel demand without comprehensive land-use protection can produce negative environmental outcomes^78^. Measures with few trade-offs when implemented in isolation, such as our diet measures, bear a lower risk in case of incomplete regulation.

As our analysis shows, packaging measures help to reduce trade-offs substantially. However, some undesirable outcomes remain. The largest remaining potential trade-off is the reduction in agricultural labour demand, mainly due to dietary change. Such a reduction would probably strongly depress agricultural wages and increase labour migration and unemployment—a dynamic that our model cannot yet show. Labour demand in the FST_SDP_ falls by an additional 192 million people on top of the reduction of 389 million in the period 2020–2050 in the BASE_SSP2_ reference scenario. Our study shows that new employment opportunities in agro-environmental practices and the bioeconomy can only slow down structural change, but that the largest part of the surplus workforce needs to be absorbed outside the agricultural sector. This can be facilitated, for example, by providing retraining or mobility schemes, cash transfers for older workers who may find no alternative livelihood, or promoting hybrid business models such as direct marketing, on-farm processing or agri-tourism^54,79–81^. If handled well, the challenge of structural change could be turned into an opportunity, using production factors more efficiently for slim and green growth^81^.

When considering the outcome trajectories over time (Fig. 2), we also observe that food expenditures first increase in the FST_SDP_ relative to BASE_SSP2_. This can be attributed to an earlier decrease of food insecurity in the FST_SDP_ and correspondingly an earlier increase in demand, as well as the increased scarcity in agricultural markets due to environmental protection and higher wages. Only towards 2050, when population growth declines and the reduction in overconsumption prevails over the reduction in food insecurity, do food expenditures in the FST_SDP_ fall below the BASE_SSP2_.

**Fig. 5:**
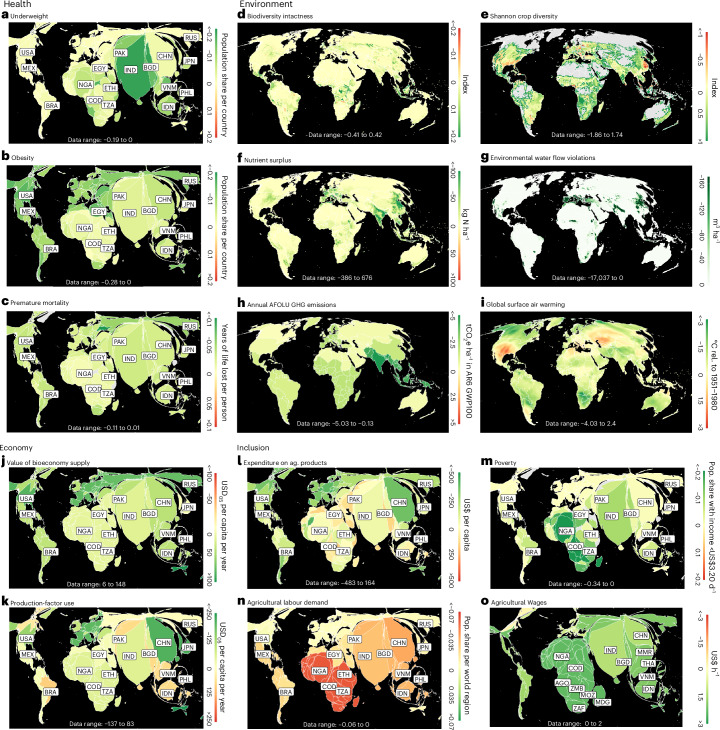
Difference in the 15 food system outcome indicators between the FST_SDP_ pathway and the reference scenario BASE_SSP2_. **a**–**o**, underweight (**a**), obesity (**b**), premature mortality (**c**), biodiversity intactness (**d**), croparea diversity (**e**), nitrogen surplus (**f**), environmental water flow violations (**g**); greenhouse gases from agriculture, forestry and other land use (**h**), global surface air warming (**i**), bioeconomy supply (**j**), production-factor use (**k**), expenditure on agricultural products (**l**), poverty (**m**), agricultural labor demand (**n**) and agricultural wages (**o**). A sustainable development pathway with food system transformation could achieve large food system co-benefits (green) for health, environment, inclusion and the economy by the year 2050, yet geographical patterns differ, and some trade-offs (red) occur, most importantly with respect to ‘agricultural labour demand’ and ‘production-factor use’. Spatial resolution differs by indicator (Table 1), and we use a Mollweide projection (area-preserving projection; per-ha values are per total cell area) for environmental indicators, and a cartogram projection (areas proportional to population or agricultural workers) for health, inclusion and economy indicators. A description of the outcome indicators can be found in Table 1.

Moreover, regional trade-offs or displacement effects exist. For example, while ‘biodiversity intactness’ generally improves, there are some regions where it deteriorates (Fig. 5). Similarly, while the minimum environmental water flows are protected in the FST_SDP_, displacement effects from water protection create additional moderate water stress in several regions that were previously unaffected (Extended Data Fig. 1k).

Our assessment shows that a comprehensive FST can achieve win–win outcomes for most people. Yet the achievement of the transformation requires that many robust empirical relationships of the past, for example, the nutrition transition aligned with economic growth^2^, or the growth of pollution with the growth of production, are discontinued. As such, it really requires “a fundamental, system-wide reorganization across technological, economic and social factors, including paradigms, goals and values”^16^ and depends, for example, on regulating market failures such as externalities in the case of environmental pollution, changing individual preferences and cognitive biases in the case of dietary change, or achieving international cooperation in the case of reducing trade barriers. As our results reveal, each FSM is connected to trade-offs, such that a stepwise introduction will usually face opposition from specific societal groups. Our results thus support the finding that measures need to be bundled to make an FST feasible^82^. A positive example of an inclusive process that overcame stakeholder opposition is the consensus between lobby groups achieved by the Zukunftskommission Landwirtschaft through bundling policies for a comprehensive agricultural transformation in Germany^80^.

While a positive vision and its detailed elaboration are crucial for guiding transformative social change^13–15,80^, the development of a vision should not only be inclusive in its outcome, but also participatory in problem framing, solution design and prioritization^83^. Our scenarios are a contribution to this evolving societal debate about a sustainable and socially desirable global food system, supporting the building of a shared vision among the wider public^13,14^. The precise configuration of FST, including the combination of measures, their ambition level, and their optimal timing, should remain open to further research and to normative debate. National assessments of FST pathways, as done for China^84^, Brazil^85^ or India^86^, can contextualize the measures needed. Our study design—allowing for multi-measure and multi-criteria comparisons—facilitates such an open and flexible process.

While our study investigates concrete measures for the FST, we do not assess the wide range of possible policies that could be used to implement them^24^. Available policy instruments include market-based instruments such as water permit trade, nitrogen surplus taxes, or consumer taxes on sugar-sweetened beverages; abolishing subsidies with misplaced incentives such as fertilizer subsidies; regulations such as minimum standards for public catering or impeding advertisements for unhealthy food items; regional, city and landscape planning, for example, for rewetting wetlands, establishing wildlife habitats or inclusive food environments; financial transfers such as income transfers or free nutritious school meals for vulnerable groups; and an extension of social infrastructure such as public canteens, farmers markets and school gardens. Moreover, governance can also originate from the cultural sphere of values, narratives and rituals that direct many of our daily actions. The choice of policy instruments for the implementation of measures is context specific and may be restricted by the absence of necessary institutions and governance capacity, and impose different transaction costs of policy-making, monitoring and enforcement, cause misallocation and leakage effects by imperfect regulation and have different distributional outcomes. Research on such policy instruments is essential, and we hope that our quantitative scenario assessment can provide benchmarks for the necessary ambition of policy packages^24^. By applying a holistic perspective across health, environment and inclusion, our study facilitates a comprehensive public dialogue on sustainable FST.

## Methods

### Modelling framework and study design

This assessment was carried out by the Potsdam Integrated Assessment Modelling (PIAM) framework, which is a cluster of models that exchange information not during run time but from consistent stand-alone simulations (soft link). For this study, the open-source land and food system modelling framework MAgPIE^20,87^ is the central model. It is linked with an open-source food demand model^2^; the open-source vegetation, crop and hydrology model LPJmL^33,34^; the reduced-complexity climate model MAGICC^35,88,89^; and a poverty distribution model^37^ as well as the open-source macro-economy and energy model REMIND^38^. The food demand model is further linked with a dietary health model^90,91^. Figure 1 lists the linkages between the individual models, which parameters are exchanged and which outcome indicators are estimated by which model. The modelling framework was run for a total of 40 scenarios, including the reference scenario SSP2, the 4 other baseline SSPs, a run for each of the 23 FSMs and 5 CrossSector measures in isolation, 5 packages of measures, the FST_SSP2_ and the FST_SDP_. The definition of the outcome indicators and the implementation of the FSMs are described in Tables 1 and 2 well as in Supplementary Data 1. Supplementary Section 1.4 provides a detailed description of the model implementation of each FSM, including the major dynamics it causes in the modelling framework and the modelling limitations with respect to non-represented dynamics.

### Land system modelling framework: MAgPIE

The central component of this modelling framework is the land and food system model MAgPIE^20,87^, which is in itself a modelling framework with multiple hard-coupled modules. The open-source model code and documentation for version 4.7.3 used in this study are available online^92^. The model simulates agricultural markets for 19 different crop groups, 8 processed plant-based product groups (sugar, oil, alcohol, oilcakes, molasses, ethanol, brans, brewers’ and distillers’ grains), 5 livestock food groups (ruminant meat, milk, pig meat, poultry meat, eggs), 3 types of crop residues (cereal straw, fibrous and non-fibrous residues), grass and 2 forestry products (timber, fuelwood). Final demands include food demand (‘Food Demand Model’), material demand and bioenergy demand^93,94^ (Supplementary Section 1.1.2). Livestock products require feed^95,96^ (Supplementary Section 1.1.3), processed products require primary products (Supplementary Section 1.1.4) and crop production requires seeds. Global production needs to meet global demand, with trade between world regions balancing regional production and demand (Supplementary Section 1.1.5). Crop, grass and forestry production require land for cultivation. Land can be intensified through yield-increasing technological change^97^ or irrigation expansion^50^ or by relocating crops to the highest-yielding areas. Land allocation is driven by the cost-effectiveness of different land uses (cropland, pasture, built-up land, forestry, forest, other land) across space, as well as land conversion costs^98^ (Supplementary Section 1.1.8). Land use change causes CO_2_ emissions from the clearing of vegetation (Supplementary Section 1.1.11) and changes the BII value of the land (Supplementary Section 1.1.9). Soil carbon levels are affected by land use change, but also depend on agricultural management (Supplementary Section 1.1.10). Irrigated production requires water and irrigation infrastructure, which can also be expanded into new areas^50^ (Supplementary Section 1.1.12). Crop and grass production requires nitrogen, which needs to be provided through the recycling of organic materials, biological fixation, inorganic fertilizers or soil depletion^57^ (Supplementary Section 1.1.7). Agricultural production causes non-CO_2_ GHG emissions (Supplementary Section 1.1.11) that include CH_4_ from enteric fermentation, water management of rice fields and manure management. N_2_O emissions derive from fertilization of crop and pasture soils as well as animal waste management and residue burning^57^. Emissions can be mitigated using technical mitigation measures^99^ (Supplementary Section 1.1.11). Technological progress is simulated via endogenous yield-increasing technological change (Supplementary Section 1.1.8) and via exogenous trajectories for livestock-productivity and feed-basket compositions (Supplementary Section 1.1.3), labour productivity and capital–labour ratios (Supplementary Section 1.1.6) and nitrogen use efficiencies (Supplementary Section 1.1.7), as well as mitigation measures (Supplementary Section 1.1.11). To find a plausible scenario for the future, the model minimizes total costs while being subject to a number of biophysical, technological and socio-economic constraints. Total costs include factor costs for labour and capital for agricultural production (Supplementary Section 1.1.6), investment costs into yield-improving technologies and management practices^97^, land expansion costs and fertilizer costs, as well as, in some scenarios, taxes for environmental pollution. Another set of costs is internal to the model as their markets are represented explicitly in the model. This includes, for example, the costs for feed and seed; land rents, which derive from the scarcity of land and land expansion costs; and the costs for nutrients from crop residues and manure. ‘Agricultural labour demand’ depends on the factor requirements for agriculture, the labour-capital share, labour productivity and weekly working hours (Supplementary Section 1.1.6). Agricultural prices, which are required for estimating ‘expenditure on agricultural products’*,* can be derived as the Lagrange multiplier of the food demand equation, providing the marginal costs of supplying the agricultural products for one additional unit of food in a given world region.

### Food demand model

The food demand model^2^ estimates a consistent set of scenarios for food intake, food waste, dietary composition, the distribution of body weight along five body mass index (BMI) classes and body height on a country level. Shifts in dietary composition over time are projected for four main food groups, that is, animal-source foods; empty calories from oils, sugar and alcoholic beverages; and staple foods, as well as calories from fruits, vegetables and nuts. A further split to the 25 food items in MAgPIE is implemented according to observed relative shares on the country level. Anthropometric and intake estimates differentiate between males and females, as well as between different age groups. Drivers of the model are the demographic composition of a population by age and sex, physical activity levels, the starting distribution of body height and the per-capita income as a proxy for the socio-economic development state of the food system.

Historical food waste (defined as household-level food waste and food losses in gastronomy and retail) is derived as the difference between Food and Agriculture Organization (FAO) food calorie supply^100^ and the food calorie intake estimated based on observed body weight, physical activity levels, age and sex. For baseline scenarios, the food waste ratio (food calorie supply per food intake) is projected using a regression with per-capita income^2^. As we estimate food intake and food waste top-down only from the energy balance, the composition of food waste with respect to different products was inferred from food-group-specific waste estimates^101^.

Within the architecture of soft-linked models, the country-level results of the food demand model are passed on to MAgPIE, the health model and the poverty model. In this study, our food demand system is inelastic to price changes from MAgPIE.

### Crop, vegetation and hydrology model: LPJmL

The LPJmL is a global dynamic vegetation, hydrology and crop model, dynamically computing soil and vegetation dynamics in natural and managed (croplands, grasslands, biomass plantations) ecosystems, explicitly accounting for water, carbon and nitrogen fluxes within and between ecosystems^33,34^. For this analysis, LPJmL computes crop yields for 12 different annual field crops for purely rainfed and fully irrigated production systems as well as corresponding irrigation water requirements, carbon stocks of potential natural vegetation and river discharge as an indicator of freshwater availability. All scenarios include CO_2_ fertilization. CO_2_ fertilization is still uncertain in magnitude, but experimental evidence shows substantial yield-increasing and water-saving effects^102^. Nitrogen limitation of crop growth is ignored here because economic decision-making on production intensity and corresponding nitrogen input requirements is accounted for in the MAgPIE model. Crop yields and irrigation water requirements are computed with the nitrogen version of LPJmL^34,103,104^, while natural vegetation dynamics, including carbon stocks and freshwater availability, are computed with LPJmL version 4 (ref. ^33^).

As such, crop yields, water requirements, carbon stocks and water availability were computed ex ante for specific climate scenarios, which could then be selected according to the projected global mean temperature (Climate Models).

### Health model

We used a global risk-disease model with country-level detail to estimate the impacts that dietary changes related to the different food-system interventions could have on disease mortality^90^^,91,105^. The model uses a comparative risk assessment method that relates changes in risk factors, such as reductions in the consumption of fruits and vegetables, to changes in cause-specific mortality, such as cancer and coronary heart disease^106^. The same concept forms the basis of the Global Burden of Disease project that tracks the impacts of different risk factors on mortality and morbidity in different regions and globally^107^.

The comparative risk assessment model used here included eight diet and weight-related risk factors and five disease end-points. The risk factors were high consumption of red meat and low consumption of fruits, vegetables, nuts and legumes, as well as being underweight, overweight and obese, the latter of which are related to changes in energy intake. The disease end-points were coronary heart disease, stroke, type 2 diabetes mellitus, cancer (in aggregate and as colon and rectum cancers) and respiratory disease.

The model uses publicly available data sources to parameterize the comparative risk analysis. We adopted relative risk estimates that relate changes in risk factors to changes in disease mortality from a meta-analysis of prospective cohort studies^108–114^. Age-specific mortality and population data were adopted from the Global Burden of Disease project^115^, and baseline data on the weight distributions of countries were adopted from a pooled analysis of population-based measurements undertaken by the NCD Risk Factor Collaboration^116^. A detailed model description is provided in Supplementary Section 1.2.

### Climate models

Our modelling framework establishes consistency between global warming levels and biophysical climate impacts by first using a reduced-complexity climate model to estimate each scenarios’ global warming outcome. This informs the selection of a precalculated high-resolution daily weather projection under future climate change derived from an Earth System Model (ESM) for each scenario. Subsequently, these spatially explicit projections—not available from the reduced-complexity climate model—are used by the crop model, LPJmL, to simulate crop yields, carbon densities and water availability. These datasets then serve as input in a second, final set of MAgPIE simulations.

The first step in this process was to use the reduced-complexity climate model MAGICC^35,88,89^ v7.5.3 to generate a probability distribution of projected global warming (Supplementary Fig. 27) using GHG emissions from the land system (MAgPIE) and the rest of the economy (REMIND)^38^ for each of our scenarios. We ran MAGICC with a probabilistic setup following the latest WG1 report of the IPCC (see Cross-Chapter Box 7.1 in Chapter 7 of AR6 WG1 (ref. ^117^)). For emissions not included in REMIND–MAgPIE (for example, Montreal Protocol species), we followed methods from the latest WG3 report^118,119^. As input to MAGICC, we combined AFOLU emissions from MAgPIE (CO_2_, CH_4_, N_2_O) with non-AFOLU emissions (for example, energy, transport, industry, waste) from previous REMIND scenarios (‘Macro-Economy and Energy Model: REMIND’), while ensuring coherence between bioenergy demand and energy transformation ambition across the modelled scenarios. For scenarios without a matching REMIND scenario (specifically SSP3, SSP4 and SSP5 baselines), we do not report global surface temperatures.

In the second step, we harmonize the high-resolution weather projections that are required to run LPJmL with the estimated global warming from MAGICC. For the weather projections, we used a single ESM for reasons of consistency because we did not want the climate signal to be overlayed by differing ESM-specific biases. We selected MRI-ESM2 (ref. ^39^) because it provided a large set of simulations for different RCPs^120^ within the CMIP6 ScenarioMIP^121^. For each scenario, we selected the CMIP6 MRI-ESM2 simulation with the RCP in which the deviation in global surface temperature between the MAGICC and the MRI-ESM2 simulation for 2050 and 2100 was smallest. This process was robust to varying the RCP used in the initial run, as the second-order feedback of climate impacts on emissions is small.

This process resulted in our primary scenarios ranging from RCP 1.9 (FST_SDP_) to RCP 6.0 (BASE_SSP2_). For scenarios based on SSP 3, 4 and 5, complementary REMIND scenarios were unavailable, so we used the standard RCP 7.0, RCP 4.5 and RCP 8.5 climate impacts, respectively. These scenarios, however, mainly served the purpose of sensitivity analysis and are not prominently featured in our analysis.

On the basis of this mapping, LPJmL receives daily weather projections from the MRI-ESM2 (ref. ^39^) model’s contribution to the CMIP6 ScenarioMIP^121^, which were made available in bias-corrected form by the ISIMIP project phase 3 (refs. ^122,123^). Atmospheric CO_2_ trajectories are taken from the corresponding SSP–RCP combinations^121^. The maps in Fig. 5 and Extended Data Fig. 3 are also based on the corresponding MRI-ESM2 projection.

### Poverty model

A distributional model^37^ is used to create projections of income distribution and poverty rates. The model starts by constructing baseline lognormal income distributions from average incomes and scenario assumptions for the Gini coefficient^124^, a measure of income inequality. Any increased ‘expenditure on agricultural products’ stemming from implementing FSMs, if applicable, is translated into their impact on average real incomes and inequality levels based on an empirical estimation of food expenditure-income elasticities. To better represent the tails of distribution relevant to poverty, the new average incomes and Gini coefficients are then fed into a regression-based model fit to recent World Bank poverty and inequality data to derive scenario projections for future poverty headcounts, accounting for the effect of potentially increased food prices.

Using the partial-equilibrium model MAgPIE, we need to safeguard macroeconomic consistency when investigating poverty effects. Increased production costs for food items due to higher labour and capital requirements get reflected in higher ‘expenditure on agricultural products’ and lower real incomes of the model. In scenarios in which food expenditures rise owing to taxes (the CO_2_ tax in the FSMs REDD+, PeatlandRewetting, SoilCarbon and the penalty for violating rotational rules in the CropRotations scenario, as well as packages including them; see Supplementary Section 1.4), the generated tax revenues are redistributed to citizens. We assume a distributionally neutral redistribution of tax revenues (broadly similar to a reduction of the value-added tax) but do not include any specific pro-poor redistribution policies (see ref. ^37^ for a discussion of their effects). Similarly, we take into account that the wage increases from the MinWage scenario (Supplementary Section 1.4.2) do not only increase prices but also have an income effect. We assume again a neutral distribution to the entire population as our income data do not allow us to distinguish agricultural income from other sources of income. As such, our MinWage scenario mainly reflects the regressive effect of higher food prices on consumers, but not that mainly low-income households would benefit from a minimum wage in the agricultural sector.

### Macro-economy and energy model: REMIND

We use the global multiregional energy–economy–climate model REMIND version 2.1.3 for our analysis^27,38,125,126^. REMIND is open source and available via GitHub at https://github.com/remindmodel/remind. The technical documentation of the equation structure can be found at https://rse.pik-potsdam.de/doc/remind/2.1.3/. In REMIND, each single region is modelled as a hybrid energy–economy system and is able to interact with the other regions by means of trade. The economy sector is modelled by a Ramsey-type growth model, which maximizes utility, a function of consumption. Labour, capital and end-use energy generate the macroeconomic output, that is, gross domestic product (GDP). The produced GDP covers the costs of the energy system, the macroeconomic investments, the trade of a composite good and consumption. The energy sector is described with high technological detail. Population, labour productivity growth and educational attainment are exogenous assumptions taken from the SSPs^127,128^.

REMIND calculates CO_2_ emissions from fuel combustion and industrial processes, CH_4_ emissions from fossil fuel extraction and residential energy use, and N_2_O emissions from energy supply by source using region- and fuel-specific emission factors. REMIND estimates CO_2_ emissions from cement production econometrically as a function of capital investments, and CH_4_ and N_2_O emissions from waste handling as a function of population and GDP. N_2_O emissions from transport and industry are exogenous baselines. CH_4_ and N_2_O from open burning are exogenous and assumed constant at the 2005 levels. Emissions of other GHGs (that is F-gases, Montreal gases) are exogenous and mapped to the corresponding SSP–RCP scenario of the IMAGE model. SO_2_, black carbon, organic carbon, NO_*x*_, CO, volatile organic compounds and NH_3_ emissions from fossil fuels depend on the endogenous combustion estimates and have emission factors that decline over time to represent improved air pollution policies based on refs. ^129,130^. We use exogenous trajectories for the emissions from international shipping and aviation and waste of all species^131^.

REMIND provides the bioenergy demand for MAgPIE and the anthropogenic emissions for all sectors except for AFOLU for the MAGICC climate model. For the SSP baseline scenarios and all transformations targeting land use in isolation, we assume that the energy transformation meets current national policies implemented and determined contributions, but no other progress is made in limiting emissions. For computational reasons, we did not couple the REMIND model and the MAgPIE model directly within this multi-scenario assessment but relied on existing runs of this well-established model ensemble^27^. These runs included a baseline scenario for SSP2 (which we used for BASE_SSP2_, all food-system FSMs and FST_SSP2_), a baseline run for SSP1 (used for BASE_SSP1_), a mitigation scenario for SSP2 (used for EnergyTrans) and a mitigation scenario for a Sustainable Development Pathway including the adoption of a planetary health diet (used for CrossSector and FST_SDP_). These coupled runs consider also production costs from MAgPIE in the macroeconomic budget equation of REMIND, determining saving and growth rates.

### Reporting summary

Further information on research design is available in the Nature Portfolio Reporting Summary linked to this article.

## Supplementary information

Supplementary Information Supplementary Figs. 1–34, Tables 1–6, Methods and Results.

Reporting Summary

Supplementary Data 1 Spreadsheet with a measure-indicator table and crop rotation specifications.

## Data Availability

A data package is available under the Creative Commons Attribution license CC BY 4.0 and archived at 10.5281/zenodo.17233328 (ref. ^132^). This package includes all input data for the MAgPIE model and the food demand model including model input from the LPJmL and REMIND models, the raw results of MAgPIE model and food demand model runs, raw results of the MAGICC model, raw results of the health impacts model, derived intermediate analysis data and the final figures, as well as instructions to reproduce any of these results.

## Code availability

The MAgPIE code, including the food demand model, is available under the GNU Affero General Public License, version 3 (AGPLv3) via GitHub (https://github.com/magpiemodel/magpie/tree/v4.7.3-FSEC, last access: 30 October 2025). The technical model documentation is available under https://rse.pik-potsdam.de/doc/magpie/4.7.3/ (last access: 30 October 2025).

The LPJmL code is available under the GNU Affero General Public License, version 3 (AGPLv3), and the code used here to generate inputs for MAgPIE can be found via Zenodo at 10.5281/zenodo.7912370 (ref. ^133^).

The REMIND code is available under the GNU Affero General Public License, version 3 (AGPLv3) via GitHub at https://github.com/bs538/remind/tree/SDP_runs (last access: 30 October 2025).

The code used to run MAGICC is available via GitLab at https://gitlab.com/magicc/2022-fsec-integration (last access: 30 October 2025).

Model documentation of the health model is available in ref. ^90^, and the model documentation of the poverty model is available in ref. ^27^.

For output processing, the m4fsdp package has been used, which is via Zenodo at 10.5281/zenodo.7899913 (ref. ^134^).

For reproduction, a package containing a copy of the used versions of MAgPIE, the food demand model, MAGICC and the m4fsdp R package is available under the Creative Commons Attribution license CC BY 4.0 and archived at 10.5281/zenodo.17233328 (ref. ^132^).
