# Supplementary material for: A food system transformation pathway reconciles 1.5 °C global warming with improved health, environment and social inclusion
Source: Nat Food. 2025 Dec 19;6(12):1133–52. doi: 10.1038/s43016-025-01268-y (PMC12716996; doi:10.1038/s43016-025-01268-y)
Supplement: Supplementary file 1 — Supplementary Figs. 1–34, Tables 1–6, Methods and Results. [file 43016_2025_1268_MOESM1_ESM.pdf]

nature food

Supplementary information https://doi.org/10.1038/s43016-025-01268-y

A food system transformation pathway reconciles 1.5 °C global warming with improved health, environment and social inclusion

In the format provided by the authors and unedited

S1: Supplementary Methods

S1.1: Extended MAgPIE description

S1.1.1 Spatial Resolution

MAgPIE operates at different spatial resolutions. Food demand is estimated on the country level, while international trade and technological progress are estimated on the level of world regions, and land allocation, crop production and nitrogen budgets on the level of 59199 0.5°x0.5° grid cells. For computational reasons of the non-linear optimization algorithm, land allocation cannot be optimized on the level of 0.5°x0.5° grid cells. Instead, an aggregation algorithm[145] conjoins the grid cells to cluster with similar biophysical and socio-economic conditions. After optimization, these clusters are again disaggregated to 0.5° cells using high-resolution data such as historical land use or crop yield potentials. World regions and clusters are flexible. In the model-setup applied for this assessment we use 14 world regions (Supplementary Fig. 1) and simulate all scenarios for both 200 and 1000 cluster cells. For computational reasons, the 200 cluster cell run is used to derive international trade-patterns which are then fixed for the parallel optimization of the world regions in the 1000 cluster setup. All results are reported for the 1000 cluster setup.

For presenting the results, the 14 world regions are aggregated based on current per-capita income into Low-income regions (LIR), Middle-income regions (MIR), and High-income regions (HIR). This aggregation is static over time, so countries do not move from one region to another when their income changes.

S1.1.2 Material and bioenergy demand: 1st generation bioenergy, 2nd generation bioenergy, forestry products, bioplastics, other materials.

Additional to the food demand, there is also a historically considerably smaller demand for other usage of agricultural products. The FAOSTAT Commodity Balance Sheet[146] estimate of this "other usage" was split into 1st generation bioenergy usage (ethanol, biodiesel) and other material demand using the World Energy Balances[147]. The other material demand was further split into a fraction used for bioplastics and a fraction used for other material purposes (e.g. industrial starch, cosmetics) using estimates by the Institute for Bioplastics and Biocomposites[148]. 1st generation bioenergy usage was projected into the future using country-specific bioenergy targets[92]. Additional to first generation bioenergy demand, there is demand for 2nd generation bioenergy made from cellulosic fast-growing grasses like miscanthus and trees like poplars which are cultivated on croplands. While these 2nd generation bioenergy crops have no substantial market share today, they are projected to have large relevance for the future. Scenarios for 2nd generation bioenergy crops were derived from a previous model exercise[26], where the MAgPIE model was iterated with the REMIND energy and macroeconomy model (see Methods section Macro-Economy and Energy Model (REMIND)).
Future baseline demand for the forestry products "industrial roundwood" and "woodfuel" is estimated by GDP and population changes as described in Lauri et al.[149] and Mishra et al.[97].

The production of bioplastics requires biomass in the form of oils, sugar, starch, glycerol, or cellulose. We assume that for future projections, the composition between different sources for bioplastic production stays constant based on current shares[150]. Glycerol is assumed to be sufficiently available as a byproduct from other production (e.g. biodiesel). Oils and sugar are included in MAgPIE as processed goods, while starch and cellulose demand was translated to primary agricultural products using conversion factors from literature[148,151–153]. For all biomass sources we assume the same conversion factor to bioplastic, which we keep constant over time[150]. The demand for bioplastics depends on scenario assumptions (see section S1.4).

S1.1.3 Livestock management and feed demand

Livestock production requires feed, which is estimated as the product of regional livestock production and regional feed baskets that vary by modeled livestock commodity[94,95]. The feed baskets comprise food and forage crops cultivated on cropland, by-products from food processing such as oilcakes, crop residues as well as grazed biomass. They were derived from feed energy balances for different animal food systems (beef cattle, dairy cattle, pigs, broilers and laying hens), using feed energy requirements per output for each animal food system, as estimated by *Wirsenius*[154]. Feed energy requirements cover growth, maintenance, lactation, reproduction, other basic biological functions of the animals as well as a general allowance for temperature effects and basic activity.

To project future developments of region- and product-specific feed baskets over time, we calculated feed conversion and feed composition for future time steps (e.g. the share of concentrates in the feed mix) based on exogenous scenario assumptions about future regional trajectories of livestock productivity (annual production per animal [ton/animal/year]), using non-linear regression models for feed conversion and feed composition with livestock productivity as predictor[94,95]. As livestock productivity increases, feed baskets shift towards more concentrate feeds, feed conversion improves, and Production-Factor Use for livestock management increases [94,95]. Region- and product-specific livestock productivity is derived from the FAOSTAT database[146] for historical time steps. For future time steps, the development of livestock productivity over time is aligned with the SSP narratives[155].

S1.1.4 Food processing

Our food processing implementation is aligned with the *FAOSTAT commodity balance sheets*[146]. As such, only the primary processing stage is explicitly captured by the model. This includes milling of cereals, pressing of oil crops into oils and oilcakes, refining of sugarcane, sugarbeet and maize into sugars and glucose syrup, as well as alcohol fermentation and distillation. Additionally, ethanol production for biofuels is estimated, resulting in the byproduct of distillers grains for feed. The couple products of processing often cause the relatively inelastic demand within MAgPIE to diverge between the two couple products. To avoid that couple products are wasted, we allow for substitution of oilcakes and brewer grains by unprocessed oilcrops in the case of excess oilcake demand, and for an elastic demand for ethanol and oil for materials at a low price in the case of excess oil or ethanol production. This can cause fluctuations of the bioeconomy supply even when the scenarios do not address bioeconomy directly.
Further processing in FAOSTAT is implicit to the conversion ratio of agricultural raw products

being used as food to final calories available for consumers. It represents all conversions and losses between agricultural markets and the retail level, while retail losses and household waste are still included in the caloric food supply estimates by FAOSTAT. Our current model does not allow for changes in secondary and tertiary food processing.

S1.1.5 Trade

Agricultural demand has to be met by agricultural production via trade. The model reflects two types of trade; intra-regional transport with the costs of transporting a product to the next population center with >40 000 inhabitants, and international trade between major world regions.

International trade is modelled following a cost-optimization approach constrained by historical trade patterns[132]. The optimization algorithm matches global imports and exports by minimizing costs. Costs include production costs, as well as export and import tariffs as well as trade margins (transport and administrative costs) from the GTAP 7 database[156]. Countries increase imports when they produce at higher marginal costs than the cheapest region that can export.

Additionally, trade self-sufficiencies (production / demand) of world-regions are constrained to only move within a corridor around product-specific benchmark levels. These benchmark self-sufficiencies, which form the centre of the corridor, continue historical trade patterns. For importing countries, benchmark self-sufficiencies stay constant at historical levels. Exporting countries have to satisfy a fixed share of global exports based on historical levels, and their benchmark self-sufficiencies are calculated accordingly. Keeping also self-sufficiencies of exporting countries constant would cause an imbalance between global imports and exports if the demand in different regions grows at different speeds.

The corridor's width is defined by a scenario parameter. The tunnel is constrained by a lower and an upper constraint, which divides or respectively multiplies the benchmark self-sufficiencies by this scenario parameter. The scenario parameter differs by storyline (see S.1.3) and is wider in the LibTrade scenario (S1.4.2). In $BASE_{SSP2}$, it is set at 1.2 for crops and 1.1 for livestock and primary-processed products.

S1.1.6 Agricultural production, labor and capital requirements

Agricultural production requires inputs of land, labor, capital, nitrogen, water, and sinks for environmental pollution.
Labor and capital requirements for crop and livestock production are calculated based on overall value of production and respective cost shares[157]. In a first step, regional factor requirements (i.e. the sum of labor and capital requirements) for crop production are estimated as given shares of the crop specific *FAOSTAT*[146] production value. The regional factor cost shares are based on a USDA dataset on total agricultural production costs by cost type[158,159]. For livestock production, factor requirements for livestock management are estimated based on a regression with livestock yields[94,95]. In a second step, factor requirements for crop and livestock production are disaggregated into labor and capital requirements based on the labor-to-capital ratio reported by the *USDA*[159]. In future projections, the labor-to-capital ratio of crop and livestock production is modeled as a function of per-capita income.

To meet total capital requirements in crop production, which are derived from production quantities and labor-to-capital ratio, the model requires a spatially-explicitly available capital

stock. If the capital stock is not sufficiently available in a simulation unit, investments are required to top up the capital stock. The capital stock depreciates at a yearly rate of 5%. The spatial explicit capital stocks lead to higher investment costs in a growing agricultural sector in a simulation unit, and no investment costs in an agricultural sector in a simulation unit shrinking faster than the depreciation rate. It thereby also reduces the adaptation speed to changing market conditions.

Agricultural Labor Demand from crop and livestock production is estimated based on total annual labor costs, agricultural wages (in the model represented by the mean nominal hourly labor cost per worker) and the average hours worked per person in a year:

$$employment = \frac{total\ annual\ labor\ costs}{hours\ per\ year \cdot hourly\ labor\ costs}$$

Total annual labor costs are derived from labor requirements and production quantities. The average hours worked in a year are based on country-specific average weekly working hours from *ILOSTAT*[160], and are kept constant for future years. Hourly labor costs[160] are considered to reflect labor productivity in an inverse relationship. They are projected using a regression with per-capita income and calibrated on country level to be consistent with the historic datasets on employment[160], hours worked[160] and labor cost (based on value of production data by *FAO*[146], and labor cost shares by *USDA*[159]) for the calibration year 2010. The model assumes perfect labor mobility between sectors over longer time-horizons, such that the agricultural workforce can be increased or decreased without changing sectoral wage levels. Climate impacts on labor productivity[62,161] are not considered in our model.

S1.1.7 Nitrogen Budgets

Nitrogen fertilization requirements are derived based on the soil nitrogen uptake (SNUp), divided by the soil nitrogen uptake efficiency (SNUpE) to account for the losses during fertilization[56]. The SNUp is equivalent to the nitrogen within the harvested biomass and the above- and below-ground crop residues, multiplied with 1 minus the percentage of plant N derived from N_2 fixation (%ndfa) and minus the nitrogen in seeds which are merged into the plant biomass. Nitrogen fertilization requirements can be settled using soil nitrogen inputs (SNI). They include N in manure and crop residues, atmospheric deposition, and N from free-living nitrogen-fixing bacteria, inorganic fertilizers, as well as reactive nitrogen (Nr) released from soil organic matter after land conversion[56]. SNUp, SNI and SNUpE are estimated separately for croplands and pastures. The SNUpE is estimated based on a historical nitrogen budget; for future scenarios, the SNUpE is the composite of a baseline trajectory component and a mitigation component (1-SNUpE) = (1-$SNUpE_{base}$)*(1-$SNUpE_{mit}$). $SNUpE_{base}$ includes modest improvements that depend on assumptions on technological progress on the basis of the Shared Socioeconomic Pathways (see section S1.3). $SNUpE_{mit}$ is 0 in baseline runs. Nitrogen surpluses from agricultural soils are estimated as the difference between nitrogen inputs in the form of organic and inorganic fertilizers, and the withdrawals in the form of harvested biomass. This budget approach, which provides the total quantity of Nr leached, volatilized or denitrified as the Nitrogen Surplus from Agricultural Soils, can be considered a robust estimate of the various forms of nitrogen pollution on an aggregated scale[47].

Nitrogen excretion by livestock is estimated as the N in feed minus the N in the slaughtered animal. Due to lack of data on the relative proportion of manure recycled on grasslands versus croplands, manure from grass is assumed to be excreted on grasslands or recycled back on grasslands, while manure from concentrate feed is assumed to be excreted in confinements. The manure in confinements is separated between nine different animal waste management systems based on regional shares, each with different recycling and emission fractions.

For (semi-) natural vegetation (i.e. forests and other land), we assume a nitrogen budget that is roughly in a steady-state, such that nitrogen surplus equals the biological nitrogen fixation and the atmospheric deposition. However, the totals are reduced if semi-natural lands are reduced because of the expansion of agricultural or urban land.

Global *Nitrogen Surplus* is estimated as the sum of the nitrogen surpluses in croplands, pastures, animal waste management and natural vegetation. This more comprehensive coverage of nitrogen surpluses also explains our higher estimates when compared to other estimates[47,162] (see Intermediate results Nitrogen).

S1.1.8 Land requirements

Land pools are distinguished into croplands, pastures, timber plantations, natural forests (primary and secondary forests), urban (built-up) land, and other land (residual land category comprising unmanaged non-forest natural land).
Cropland requirements depend on the production and on crop-specific yields. Yield levels on 0.5° grid scale are provided by the LPJmL crop model under various scenarios of climate change (see section "Crop, Vegetation and Hyndrology model (LPJmL)" in the methods), and are calibrated to meet FAO yields at world-region level in the historical period.
To increase agricultural production, the cost-optimization algorithm finds an equilibrium between four options to manage land scarcity when faced with increasing demand or declining yields: Yield-increasing technological change[96]; expansion of irrigated areas[49]; expansion of agricultural land, spatial reallocation of crops to higher-yielding locations.

Yield-increasing technological change increases crop yields via a regional multiplicative factor on LPJmL cropping patterns, and causes investment costs[96]. The expansion of agricultural land and of area equipped for irrigation also comes at additional investment costs[49]. Irrigation moreover requires water (see S1.1.12). Reallocating crop production from lower-yielding to higher-yielding area – either within or across world regions - can increase costs e.g. through higher transport and trade costs. Moreover, more monotonous cropping patterns or reductions in fallow periods come at the expense of ecosystem services, which have to be substituted by additional management like pest control[163,164]. This is modelled through additional production costs if cropping patterns exceed typical rotation lengths or forgo fallow periods[165–167] (see SI spreadsheet).

Investment costs are included in the *Production-Factor Use* indicator. As the investment decision also depends on returns in future timesteps, we include them in the optimization function in an annuitized way, simulating a myopic foresight that assumes a continuation of current scarcities. As such, the annuities also influence shadow prices (marginal costs, estimated as Lagrange multipliers of demand constraints) and *Expenditures for Agricultural Products*.

Future urban land development is exogenously prescribed and based on SSP projections[139]. At the cluster level, urban land may deviate from prescribed levels when there is very high competition from other uses, i.e. at very high cost. Regional sums of urban level do not deviate. Urban land reduces the amount of land available for other uses.

Timber plantation area requirements are simulated based on the forestry module[97]. For each timestep, the MAgPIE model assumes that a certain share of the future wood demand (see section S1.1.2) will be fulfilled from timber plantations and the rest from natural forests. Forest plantations are established under consideration of climate-specific rotation lengths and the expected share of current demand to be fulfilled by forest plantations. The yields of forest plantations and natural forests are estimated based on LPJmL carbon estimates and growth curves with growth curves parameterized based on *Braakhekke et al.*[134]. For the land allocation of new forestry plantation establishments, there is a dynamic interplay between agriculture and forestry as they compete for limited land resources on the cluster-scale of MAgPIE, minimizing the cost of production under consideration of spatial yield potentials, reflecting a complex trade-off in resource allocation. Once established, timber plantations cannot be harvested until they reach age-classes equivalent to rotation lengths. Age-class restriction for harvesting does not exist for harvesting natural forests in MAgPIE[97]. Increasing demand for wood exacerbates the pressure on the land system, causing a dynamic reaction of the model with respect to international trade, land allocation and cropland intensification. Intensification of plantations is not considered. MAgPIE incorporates the existing NPI and NDC policies to align with afforestation targets - which earmarks additional areas not accessible for agricultural use in the model.

S1.1.9 Biodiversity

Land-use impacts on terrestrial biodiversity are captured by the Biodiversity Intactness Index (BII)[44,168], which is based on a two-step approach. In a first step, net changes in abundance within species assemblages are computed as a function of recent human land use. In a second step, the compositional similarity between sites is compared to a reference land-use class, for which primary vegetation is used (space-for-time approach). As a result, land with low human interference, such as primary forest, typically has a BII of 1, whereas secondary forest and agricultural land exhibit lower BII values (e.g. 0.5-0.7 for cropland). The BII can therefore be used to track changes among different land-use classes at a given site in an aggregated way. It is worth noting, however, that various other important land-use related drivers of biodiversity change, such as interactions among land uses, habitat location, habitat connectivity, habitat quality and land management are not covered by the BII. Based on our spatially-explicit outputs, our outcome indicators highlight two focal areas of biodiversity change, namely biodiversity hotspots, due to their overwhelming global importance for biodiversity conservation, on the one hand, and farmed landscapes, on the other hand, where ongoing biodiversity change has undermined many critical ecosystem functions that sustain land productivity. Thus the *Hotspot Landscapes* BII reports area-weighted BII values in areas that are covered by the biodiversity hotspots definition[45]. The *Cropland Landscapes* BII aggregates spatially-explicit BII values in grid cells with more than 100 Mha of cropland, and is thereby sensitive to agglomeration of crop areas that can prevent landscape heterogeneity. During aggregation, the *Cropland Landscapes* BII is weighted based on the cropland area in each grid cell. Therefore, the *Cropland Landscapes BII* decreases with increasing cropland concentration (landscape simplification) and increases when land use in cropland grid-cells becomes more diverse.

S1.1.10 Land use change greenhouse gas emissions

CO_2 emissions from land-use change and CO_2 removals from land management are based on carbon stock changes between time steps. Carbon stocks for vegetation are obtained by multiplying land areas with corresponding carbon densities from the LPJmL model. In case of deforestation or conversion of other natural land or pastures, aboveground vegetation carbon is immediately released to the atmosphere. In case of re/afforestation or succession of natural vegetation, carbon is gradually sequestered based on an age-class growth model. Carbon emissions from the removal of aboveground vegetation are based on a carbon budget using carbon densities from the LPJmL model. Once forests or other land is deforested, the carbon is assumed to be instantly released into the air. MAgPIE also tracks the long-term carbon storage in harvested wood products (HWPs) based on the IPCC tier 1 guideline[169]. MAgPIE accounts for the slow release of CO2 from existing pools of carbon stored in harvested wood products into the atmosphere from decay and disposal. Nitrogen and methane emissions from forest and savanna fires[170] are not accounted for in MAgPIE.

Soil carbon emissions from converting land into croplands are estimated using the IPCC 2019 guidelines for national GHG inventories[169]. They derive long-term equilibrium values for different climates, land types and management regimes. The estimation of long-term soil equilibria requires a reference soil carbon stock under natural vegetation, which we derive using the LPJmL model. Next, we apply modifiers of carbon densities in the first 30cm of the soil profile[169] based on landuse type (perennials, annual crops, paddy rice and fallow), tillage, and the quantity of inputs which are all climate-dependent. Climate classes are derived using the Koeppen-Geiger zones. For tillage and inputs, we use the default values for each climate class due to lack of data. For the "landuse type", we take into account that perennial crops, irrigated land and rice paddies have a higher soil carbon. As the loss and build-up of soil organic matter requires time, we assume that every year 15% of the difference between current carbon stocks and the long-term equilibrium values are built up, which results in a 96% closure within the 20 year period suggested to use by *IPCC*[169]. Based on a fixed C:N ratio, the depletion of soil organic matter also releases Nr that fertilizes crops for the nitrogen budget, a dynamic that is used e.g. in shifting cultivation systems.

GHG emissions from drained peatlands are estimated based on IPCC wetland GHG emission factors and a map of drained peatlands[136]. CO_2 from degrading peat is by far the most important GHG from drained peatlands. Future drainage of intact peatland is linked to the expansion of managed land (cropland, pasture, forestry). Likewise, the rewetting of drained peatlands requires that managed land is retired. Rewetted peatlands have considerably lower CO_2 emissions than drained peatlands. While CH_4 emissions due to anaerobic conditions are higher in rewetted compared to drained peatlands, the net effect of peatland rewetting is a reduction of GHG emissions[171].

S1.1.11 Agricultural greenhouse gas emissions

Methane (CH_4) emissions from enteric fermentation are estimated based on the amount and composition of feed provided to ruminants, using the Tier II method of the 2006 IPCC national guidelines for emission inventories[172] and feed baskets by Weindl et al.[94,95].

CH_4 emissions from manure management are accounted for through emission factors per managed manure from FAOSTAT *Emissions From Livestock* [146]. CH_4 emissions from paddy rice cultivation are derived using emission factors per cultivated rice area from *FAOSTAT Emissions from Crops*[146]. Nitrous oxide (N_2O) emission from soils and manure management are estimated based on the nitrogen budgets (SI section S1.1.7) using emission factors of the *IPCC*[172]. The original IPCC emission factors, which are dependent on fertilization rather than surpluses, violate mass balances for high nitrogen uptake efficiencies (NUE), as they would assume emissions even for a hypothetical system with a NUE of 1 where inputs only replace harvested Nr. Emission factors were therefore rescaled to account for the large variations in regional soil nitrogen uptake efficiencies, assuming that N_2O emissions are proportional to total cropland nitrogen surplus. Emission factors therefore also decrease when NUE improves through improved management (see S1.1.7).
All agricultural greenhouse gas emissions can be reduced via technical measures at additional production costs using marginal abatement cost (MAC) curves for each emission source from *Harmsen et al.*[98]. These MAC curves account for technological progress by increasing the availability, reduction and implementation potential of technologies over time [98]. In baseline scenarios, these measures are not available, but they get activated by the FSMs NitrogenEfficiency, RiceMitigation, LivestockManagement and ManureManagement (see Section S1.4.4. for details).

S1.1.12 Irrigation

MAgPIE models irrigation and irrigation expansion endogenously. To which extent cropland is irrigated depends on the yield gain that can be achieved through irrigation in a specific location for the respective crop, unit costs for irrigation area expansion, as well as water demand for irrigation. Irrigation water demand, in turn, depends on local water availability in the growing period in the respective simulation cluster, crop irrigation water requirements, and regionally-specific irrigation efficiency[132]. Water availability for irrigation in the growing period as well as crop-specific irrigation water requirements per hectare of irrigated land are derived using the global vegetation, hydrology, and crop-growth model LPJmL[32,33].

To derive irrigation water availability at the aggregated resolution of 1000 spatial clusters, monthly basin runoff at 0.5° spatial resolution, as provided by LPJmL, is allocated to its respective river basin grid cells using monthly discharge as allocation weight and then aggregated to the spatial cluster unit used in MAgPIE[49]. Exogenous non-agricultural water demand is provided by the WaterGAP model[141] and prioritized due to a higher marginal value for industry, manufacturing, and households.

To assess the environmental impact of water withdrawals, we calculate Environmental Water Flow Violations as the volume of water withdrawn that exceeds a sustainable level in each simulation cluster. To derive the environmental flow requirement volume, we use monthly discharge at 0.5° spatial resolution provided by LPJmL using the Smakthin method[137] and aggregate the respective water volume reserved for the environment to the simulation cluster level[49]. Due to the temporal and spatial aggregation, variations may be averaged out and violations underestimated, such that our estimate of the global environmental flow violation (EFV) volume is conservative. The water stress index (used in Extended Data Fig. 1) is calculated as the ratio of water withdrawals to water availability.

S1.2 Health analysis of the burden of diet-related diseases

This section describes the global risk-disease model by Springmann et al. [4,89,90,173].

Comparative risk assessment

We estimated the mortality and disease burden attributable to dietary and weight-related risk factors by calculating population impact fractions (PIFs) which represent the proportions of disease cases that would be avoided when the risk exposure was changed from a baseline situation to a counterfactual situation. For calculating PIFs, we used the general formula[105,174,175]:

$$PIF = \frac{\int RR(x)P(x)dx - \int RR(x)\,P'(x)dx}{\int RR(x)P(x)dx}$$

where RR(x) is the relative risk of disease for risk factor level x, P(x) is the number of people in the population with risk factor level x in the baseline scenario, and P'(x) is the number of people in the population with risk factor level x in the counterfactual scenario. We assumed that changes in relative risks follow a dose-response relationship[174], and that PIFs combine multiplicatively, i.e. $PIF = 1 - \prod_i (1 - PIF_i)$ where the i's denote independent risk factors [174,176].

The number of avoided deaths due to the change in risk exposure of risk i, Δdeathsi, was calculated by multiplying the associated PIF by disease-specific death rates, DR, and by the number of people alive within a population, P:

$$\Delta deaths_i(r,s,a,d) = PIF_i(r,s,a,d) \cdot DR(r,s,a,d) \cdot P(r,s,a)$$

here PIFs are differentiated by region r, sex s, age group a, and disease/cause of death d; the death rates are differentiated by region, sex, age group, and disease; the population groups are differentiated by region, sex, and age group; and the change in the number of deaths is differentiated by region, sex, age group, and disease.

Data sources

We used publicly available data sources to parameterize the comparative risk analysis. Mortality and population data were adopted from the Global Burden of Disease project[114]. Baseline data on the weight distribution in each country were adopted from a pooled analysis of population-based measurements undertaken by the NCD Risk Factor Collaboration[177].

The relative risk estimates that relate the risk factors to the disease endpoints were adopted from meta-analyses of prospective cohort studies for dietary and weight-related risks[107–113]. In line with the meta-analyses, we included non-linear dose-response relationships for fruits, vegetables, and nuts and seeds, and assumed linear dose-response relationships for the remaining risk factors. As our analysis was primarily focused on mortality from chronic diseases, we focused on adults aged 20 year or older, and we adjusted the relative-risk estimates for attenuation with age based on a pooled analysis of cohort studies focussed on metabolic risk factors[178], in line with other assessments[175,179].

Supplementary Table 3 provides an overview of the relative-risk parameters used. For the counterfactual scenario, we defined minimal risk exposure levels (TMRELs) as follows: 300 g/d for fruits, 500 g/d for vegetables, 100 g/d for legumes, 20 g/d for nuts and seeds, 0 g/d for red meat, and no underweight, overweight, or obesity. The TMRELs are in line with those defined by the Nutrition and Chronic Diseases Expert Group (NutriCoDE)[179], with the exception that we used a higher value for vegetables, and we used zero as minimal risk exposure for red meat, in each case based on a more comprehensive meta-analysis[109,112].

Certainty of evidence

The selection of risk-disease associations used in the health analysis was supported by available criteria used to judge the certainty of evidence, such as the Bradford-Hill criteria used by the Nutrition and Chronic Diseases Expert Group (NutriCoDE)[179], the World-Cancer-Research-Fund criteria used by the Global Burden of Disease project[3], as well as NutriGrade[180] (Supplementary Table 4). The certainty of evidence supporting the associations of dietary risks and disease outcomes as used here were graded as moderate or high with NutriGrade[110–112], and/or assessed as probable or convincing by the Nutrition and Chronic Diseases Expert Group[179], and by the World Cancer Research[181]. The certainty of evidence grading in each case relates to the general relationship between a risk factor and a health outcome, and not to a specific relative-risk value.

We did not include all available risk-disease associations that were graded as having a moderate certainty of evidence and showed statistically significant results in the meta-analyses that included NutriGrade assessments[110–112]. That was because for some associations, such as for milk and fish, more detailed meta-analyses (with more sensitivity analyses) were available that indicated potential confounding with other major dietary risks or health status at baseline[182–184]. Such sensitivity analyses were not presented in the meta-analyses that included NutriGrade assessments, but they are important for health assessments that evaluate changes in multiple risk factors.

For the different diet scenarios, we calculated uncertainty intervals associated with changes in mortality based on standard methods of error propagation and the confidence intervals of the relative risk parameters. For the error propagation, we approximated the error distribution of the relative risks by a normal distribution and used that side of deviations from the mean which was largest. This method leads to conservative and potentially larger uncertainty intervals as probabilistic methods, such as Monte Carlo sampling, but it has significant computational advantages, and is justified for the magnitude of errors dealt with here (<50%) (see e.g. IPCC Uncertainty Guidelines).

Caveats

In the comparative risk assessment, we used relative risk factors that are subject to the caveats common in nutritional epidemiology, including small effect sizes and potential measurement error of dietary exposure, such as over and underreporting and infrequent assessment. For our calculations, we assumed that the risk-disease relationships describe causal associations, an assumption supported by the existence of statistically significant dose-response relationships in meta-analyses, the existence of plausible biological pathways, and supporting evidence from experiments, e.g. on intermediate risk factors[107–113,179,185,186]. However, residual confounding with unaccounted risk factors cannot be ruled out in epidemiological studies. Additional aspects rarely

considered in meta-analyses are the importance of substitution between food groups that are associated with risks, and the time lag between dietary exposure and disease.

To address potential confounding, we omitted risk-disease associations that became non-significant in fully adjusted models, in particular those related milk intake[182,183], and to fish intake [184,187–190]. The quality of evidence in meta-analyses that covered the same risk-disease associations as used here was graded with NutriGrade as moderate or high for all risk-disease pairs included in the analysis[110–112] (Supplementary Table 4). In addition, the Nutrition and Chronic Diseases Expert Group and the World Cancer Research Fund graded the evidence for a causal association of ten of the 12 risk-disease associations included in the analysis as probable or convincing[179,181], The relative health ranking of leading risk factors found in our analysis was similar to existing rankings that relied on different relative-risk parameters and exposure data [3,191].

As exposure data, we used a proxy of food consumption that was derived from estimates of food availability that were adjusted for the amount of food wasted at the point of consumption[100,192]. An alternative would have been to rely on a set of consumption estimates that has been based on a variety of data sources, including dietary surveys, household budget and expenditure surveys, and food availability data[193,194]. However, neither the exact combination of these data sources, nor the estimation model used to derive the data have been made publicly available. For some individual countries, using dietary surveys would also have been an alternative. However, underreporting is a persistent problem in dietary survey[195,196], and regional differences in survey methods would have meant that our results would not be comparable between countries. In contrast to dietary surveys, waste-adjusted food-availability estimates indicate levels of energy intake per region that reflect differences in the prevalence of overweight and obesity across regions[177].

S1.3: SSP scenarios

The Shared Socioeconomic Pathways (SSPs) provide a set of baseline scenarios describing plausible alternative future evolutions of society and natural systems over the 21st century and reflecting alternative reference assumptions about future socio-economic development[7,22,155]. The scenarios have been adopted widely in the climate change community, but also by other assessments of global change (https://ciesin.columbia.edu/data/ssp-literature-database-v2-2020-2021/).

SSP1 represents a green growth future in which lifestyles of people are less material-intensive, innovation towards more efficient technologies is fast, and human development is characterized by higher equity. SSP2 describes a middle-of-the-road scenario, in which human development, lifestyles, economic growth, and technological development remains very much within currently observed trends. SSP3 describes a scenario characterized by regional rivalry, impeding fast economic and technological growth. SSP4 is a highly unequal world, in which some world regions can achieve high economic progress, while others stay behind. Also inequality within countries is very high, and technological progress is very unequal. SSP5 depicts a fossil-fueled economic development scenario leading to high socioeconomic development but also high environmental degradation[155].
In addition to a narrative storyline, the SSPs provide quantified development indicators, which include drivers such as GDP or population growth rates[126,127]. GDP projections were updated to

include the macro-economic downturn due to the Corona pandemic[51]; they do not yet account for the effects of Russia's invasion of Ukraine[12]. The GDP projections also do not include impacts of climate change and ecosystem service loss on GDP growth. The storylines moreover provide qualitative statements on the development of demographics, human development, economic development, lifestyles, technology and natural resources, which were translated into modeling parameters by different IAM teams including REMIND-MAgPIE[7]. These scenario switches assume - in line with the respective storyline - different trends for intensified livestock management[94,95], account for more or less material-intensive consumption patterns in the projections of food demand, different degrees of trade liberalization[7], a different growth of built-up land[139], different risk premia for interest rates on investments based on the quality of governance[197], different non-agricultural water demand[141] and manure management[56]. To account for the new implementation of nitrogen budgets in this assessment, the baseline soil nitrogen uptake efficiency trajectory ($SNUPE_{base}$) - reflecting technological progress in absence of nitrogen mitigation policy - was chosen in line with the qualitative SSP storylines[155], assuming no improvement in SSP3, modest improvement of 5 percentage points between 2010 and 2050 in SSP2 and in the high-tech but material-intensive scenario SSP5, and 10 percentage points between 2010 and 2050 in the scenarios SSP1 and SSP4 that are supposed to have a high mitigation potential. Moreover, the maximum SNUPE was limited to 55% in SSP3, 65% in SSP2 and 75% in the storylines SSP1,4 and 5 that assume rapid progress in technology, in order to avoid unrealistically high SNUPEs in countries which already today have high efficiencies, and to account for technological catch-up between world regions.

Our scenarios do not include short-term events such as natural disasters, conflicts, trade wars or pandemics, which according to the SSP storylines should be most prevalent in SSP3 and the least prevalent in SSP1.

All scenarios include a list of land-based measures for climate change mitigation that have been implemented by the nation states or agreed upon in the climate change mitigation pledges (National Policies Implemented (NPI) and Nationally Determined Contributions(NDCs)). The targets have been parameterized based on a screening of the official country pledges from the UNFCCC website[198].

Three key targets related to land use NPI and NDC policies are modeled in MAgPIE:

1. Avoiding Deforestation (AD): This policy focuses on preventing the loss of forested areas. It is modeled based on forest land cover and forest loss rates from initialization land pools. Through spatial mapping individual cells are associated with specific states, enabling targeted policy implementation on a country level. Policies are then defined based on parameters such as base years, target years, and target values, indicating the desired percentage rate of protection against deforestation (in percentage unit, 0 - 1). Calculated changes in deforestation rates are projected over time until established targets defined by policies. In case a country also has an afforestation target, the assumption is that the deforestation is halted by the year when the afforestation policy starts. We included the pledges of on AD of 76 countries, including the promise to either completely stop deforestation or reduce deforestation.
2. Avoiding Other Land Conversion (AOLC): Similar to AD policies, this policy addresses protecting non-forest land from conversion for various purposes (cropland and pasture). We included the pledges on AOLC of 31 countries, including the promise to either completely stop or reduce land conversion.

3. Afforestation (AFF): These policies are designed to simulate fulfilled objectives of afforestation areas in the future. Similar to other modeled NPI/NDC policies, the afforestation targets are implemented on the country level, with target values representing the desired area to be afforested (measured in million hectares). The prescribed additions in forested areas are imposed to the model, making sure that the targets are achieved smoothly over the modeled time periods. We included the pledges on AFF for 45 countries, including the respective area targets. Supplementary Table 5 lists the afforestation pledges for the countries with major targets.

S1.4: Food system measure description

S1.4.1 Diet measures

The Diet FSMs LowProcessed, HighLegumes, LowMonogastrics, LowRuminants, HighVegFruitsNuts, HalfOverweight, NoUnderweight and LowFoodWaste are implemented in the Food Demand Model and the dietary health model.
LowProcessed, HighLegumes, LowMonogastrics, LowRuminants, HighVegFruitsNuts change the dietary composition of food intake according to the food-group specific intake recommendations by the Planetary Health Diet (PHD)[6] as implemented by Springmann et al.[8], taking into account country level energy densities of food products. Energy densities diverge on country level for instance due to different crop mixes within crop groups (e.g. vegetables), different varieties of crops, or also different food processing (e.g. different meat cuts or milk processing). In line with Springmann et al[8], we used the targets from the EAT-Lancet diet in grams, as this is the unit of the dietary risk factors in the literature, leading to variation in the resulting per-capita calorie intake (Supplementary Table 1). Differing calorie intake can also originate from the possibility that baseline intake in countries exceed the minimum targets prescribed by FSMs or stay below maximum targets.
To keep total calorie intake constant, the reduction or increase of the targeted food items are counterbalanced by an inverse increase or decrease of the intake of staple crops (cereals and roots) such that total intake stays constant. Bodyweight is therefore not affected by these FSMs. In LowProcessed, the intake of sugars is capped at 31 grams per capita per day as recommended in the PHD[6,8], while the intake of plant-based oils and fats are converged towards 46.8 grams per capita per day. For the health-risk estimates, we additionally assume that grains are consumed as wholegrains. In extension of the PHD recommendations, alcohol consumption was limited to a maximum of 1.4% of calorie intake[131]. In HighLegumes, the intake of legumes is increased to 100 grams per capita per day as recommended in the PHD[6,8] in countries where these values are not already fulfilled. In LowMonogastrics, the intake of poultry meat and eggs is capped at 29, and 13 grams per capita per day as recommended in the PHD[6,8]. Red meat is capped at 14 grams in the PHD[6,8], and we define the target for pig meat based on the historical share of pig meat within red meat. In LowRuminants, the intake of milk is capped at 250 grams per capita per day as recommended in the PHD[6,8]. Again, the target for ruminant meat depends on the historical contribution of ruminant meat in red meat consumption, with the maximum value for red meat being 14 grams. HighVegFruitsNuts, the intake of vegetables, fruits, nuts and seeds is increased to 525 grams per capita per day as recommended in the PHD[6,8]. The adoption of the PHD has

been previously simulated by MAgPIE[20] however, the effects of individual food group recommendations were not isolated in these simulations.

In HalfOverweight, calorie intake is reduced to achieve a reduction of overweight and obesity by 50% relative to the BASE scenario. Calorie reduction is BMI-class-, body height-, age-group- and sex-specific and also depends on physical activity levels in the country. The intake of half of the people overweight or obese (BMI>25 for adults, BMI +/-1STD for children) is reduced to the level of intake recommended for a healthy BMI (20-25, BMI <+1STD for children).

In NoUnderweight, calorie intake is increased in line with a complete eradication of underweight until 2050 for all age cohorts and sex classes in all countries. Calorie increase is BMI-class-, country-, age-group- and sex-specific. Caloric intake of adults with BMI<20 and children with BMII < -1STD is increased to the intake recommended for a healthy BMI (20-25, BMI <+1STD for children). Both measures do not change the relative dietary composition of the diet and the intake of people in other BMI classes. The calorie intake that is consistent with a given body weight is calculated based on the Schofield equations as described in Bodirsky et al[2].

LowFoodWaste reduces the food waste in households and retail (difference between calorie intake and FAO food availability) from the baseline food waste ratio - as estimated following the method of Bodirsky et al[2] - to a maximum of 20% of intake (equivalent to a waste share in food supply of 16.66%).

The resulting food demand of the combined Diet FSMs can be seen in Supplementary Fig. 16. For all Diet FSMs, we assume a gradual transition of the endogenously estimated baseline to the FSM-specific parametrization of food waste, food intake, and diet composition until 2050. Apart from the prescribed changes in the individual FSMs, dietary change follows the baseline pathway. In the FST_{SDP} pathway, which includes the more equitable economic development of HumanDevelop, the diets are still influenced in the first timesteps through the endogenous impacts of faster economic growth, yet the dietary patterns converge fully to the exogenously prescribed *Diets* package by 2050. For all FSMs, body weight, physical activity, and caloric intake remain consistent based on the energy-balance approach described in Bodirsky et al[2].

All FSMs in the Diet package influence food demand, which is a central driver in the modeling framework, affecting agricultural production and environmental impacts. A change in food demand also alters food prices, as natural resources such as land and water become more or less scarce. *Expenditures for Agricultural Products* change in turn as a consequence of changes in both quantity and price. Changing food expenditures finally influence poverty estimates. Moreover, food intake determines health risk factors in the dietary health model.

The main dynamics triggered in the MAgPIE model by the change in dietary composition are a reduction of livestock production and feed demand and an increase of plant-based food demand. Due to the higher trophic level of livestock products, this leads to an overall reduction in the agricultural biomass production. Cultivation patterns change due to the reduced feed demand and the increased diversity of plant-based foods, influencing crop area diversity as well as total land requirements. The nitrogen cycle is altered through lower feed production, a decrease in manure excretion, less nitrogen-fixing legumes for feed and more legumes for human consumption. A decrease in agricultural production results in lower costs and labor demand, declining nitrogen and GHG emissions from agricultural production, lessening the need for land expansion and associated biodiversity loss and land-use change emission. The reduced competition for land also entails lower food prices and reduces the need to intensify production via yield-increasing technological change or via shorter crop rotations.

We do not model possible interactions between dietary composition and BMI. In reality, the changed dietary composition with higher nutrient density and lower calorie density could facilitate a reduction in overweight and obesity. The estimate of premature mortality is modeled through a limited number of risk factors and disease endpoints. While these include the major risks and diseases, they are not exhaustive. In particular, undernutrition-related diseases are less represented than overnutrition-related ones. Price- or income-induced rebound effects on diets are not considered. In the FST_{SDP} scenario, the coupled REMIND-MAgPIE run accounts for the macroeconomic consequences of a reduced Production-Factor Use in agriculture, but the growth stimulus is not substantial given the low fraction of Production-Factor Use in total GDP (Supplementary Fig. 29). We do not simulate this interaction for individual FSMs where the effect would be even lower.
Our Diet FSMs directly prescribe the level of consumption and analyze the consequences of changed consumption on our outcome indicators. As scoped in the introduction and discussion, our study does not specify the circumstances that can cause such a change in consumption behavior, whether it includes intrinsic preference changes, altered food environments, food price changes or direct regulation. While many policy instruments are known that could change dietary behavior[199–201], there are no quantitative estimates yet that point out which package of instruments could achieve a disruptive dietary change as described here.

S1.4.2 Livelihoods measures

LibTrade

The LibTrade FSM is implemented in the MAgPIE model.

In the LibTrade scenario, MAgPIE trades more goods according to relative competitiveness (lowest production and trade costs) as opposed to historical trade patterns. Within the trade module[132] (see S1.1.5), this is simulated by increasing the size of the flexible-trade tunnel via setting the flextrade scenario parameter for crops from ×/÷1.2 of domestic demand in the baseline case to ×/÷1.3 in the LibTrade scenario, and from ×/÷1.1 to ×/÷1.2 for livestock and secondary products by 2050.
Through this scenario, production shifts to more efficient production areas, leading to a decrease in production costs and a potential decrease in total area. Depending on the redirection of trade, trade liberalization can cause an increase or decrease of deforestation, greenhouse gas emissions and water scarcity[132,202]. Overall commodity prices fall due to reduced scarcity, but single regions can also experience a rise of prices if they were favored by historical trade patterns.
Our trade implementation is subject to a number of limitations, including for example the assumption of homogenous goods within commodity groups and the absence of transaction costs, leading to an underestimation of trade, which is in reality often bidirectional. Our approach also does not account for agglomeration effects of industries, which play for example a major role for the livestock industry. Moreover, as a partial equilibrium model, our model also does not consider dynamics which are caused by changes in exchange rates of currencies.

MinWage

The MinWage FSM is implemented in the MAgPIE model.
In MAgPIE, we calibrate a Cobb-Douglas production function with a substitution elasticity of 0.3[203] to match the long-term labor-to-capital ratio and labor productivity in a baseline scenario

(see S1.1.6). In the MinWage FSM, we define a global minimum wage of 3 USD05MER/h to be reached by 2050. To meet this target, we accelerate the increase of agricultural wages in low-income countries between 2020 and 2050 compared to scenarios without minimum wage. The minimum wage leads to substitution of labor by capital in line with the Cobb-Douglas function, and to a deadweight loss that increases production costs. Moreover, as the absolute minimum wage mainly affects low-income countries, it leads to a shift of production from the LIRs to MIRs and HIRs. Given that remaining biodiversity hotspots are more concentrated in tropical regions (Supplementary Fig. 2), this reduces deforestation and biodiversity loss. The MinWage FSM interacts with the Cross-Sector measure HumanDevelop. The more equitable economic growth of HumanDevelop leads to faster increases of wage rates, such that the minimum wage applies to fewer people and leads to lower reallocation effects.
Our indicator *Production-Factor Use* is calculated using constant prices per productive factor unit. In the MinWage scenario, this indicator therefore does not include the changed prices of labor, but only reflects the changes in factor quantities used in the land system (e.g., due to a substitution of labor by capital). This is intended, as the higher wage rates in agriculture do not reduce labor productivity and production potential of the rest of the economy, but mainly have a redistributive effect. A loss to the rest of the economy only occurs due to less efficient allocation of production factors in the food system when wages exceed labor productivity (the deadweight loss), resulting in higher Production-Factor Use in our model and fewer available production factors for other sectors. Our results show the counter-intuitive results that Production-Factor Use in the year 2050 is lower in MinWage than in $BASE_{SSP2}$. This originates from the regional relocation of production to HIRs, which pulls investments into technological change forward in time; costs in all timesteps before 2050 are higher in MinWage than in $BASE_{SSP2}$.
In contrast, our indicator *Expenditure for Agricultural Products* includes not only the impacts of less efficient factor allocation, but also the impacts of higher wage rates on marginal costs and prices. Expenditures therefore rise with the introduction of the minimum wage. Our *Poverty* Indicator is driven by changing expenditures, reflecting the regressive effect of higher food prices on consumers. Additionally, we take into account in a simplified way that a higher wage rate also results in higher incomes, partially offsetting rising expenditures. We redistribute the wage rent *((minimum wage rate – baseline wage rate) * employment)* in an income-distribution-neutral way to the entire population, as our model cannot differentiate population groups with different factor endowments. In reality, the income effect would positively affect households with agricultural workers, while urban households would primarily face higher prices. Poverty in rural households would therefore decline while it would increase in urban households. The net effect depends inter alia on the share of households with farm income and the distribution of poverty between farm and non-farm households.
A limitation of our sector-model approach is that we assume that in the labor-capital substitution, further capital is available at the same interest rate. In reality, particularly small farmers with limited equity capital may have problems acquiring additional capital, resulting in an even stronger shift of production to HIRs and a higher deadweight loss. A further limitation is that the increase of prices does not lead to changes in our inelastic food demand.

CapitalSubst

The CapitalSubst FSM is implemented in the MAgPIE model.
In MAgPIE, we calibrate a Cobb-Douglas production function with a substitution elasticity of 0.3[203] to match the long-term labor-to-capital ratio and labor productivity in a baseline scenario (see S1.1.6). In the CapitSubst FSM, we assume that in regions with high capital intensities,

capital in crop production is partially replaced by labor to reduce the loss of employment. The global target for the capital share (i.e the capital requirement share out of labor and capital requirements) in crop production is set to 20% by 2050. To allow for regional differences, this share is not strictly enforced. Instead, if countries exceed this capital share, we reduce the gap between the baseline labor share and the target share by 50%.

Setting the resulting capital shares as a constraint directly leads to substitution of capital by labor; the use of the above Cobb-Douglas production function (see *MinWage* FSM) results in an increase in Agricultural Labor Demand, a decline in capital, and a net increase in the Production-Factor Use.

Indirect effects are lower competitiveness of regions with a formerly high capital share, leading to a relocation of production in regions which are not affected by this FSM, which also increases Agricultural Labor Demand. Moreover, marginal costs of production rise, leading to higher prices and as a consequence higher food expenditures and poverty.

The measure interacts with the *HumanDevelop* scenario. In the *HumanDevelop* scenario, the labor-to-capital ratio declines faster due to stronger economic development. In the FST_{SDP} pathway, which includes *HumanDevelop*, the *CapitalSubst* measure closes 50% of the gap relative to the *HumanDevelop* scenario, which means that the labor-to-capital ratio is lower than in the *CapitalSubst*-only scenario. This reflects that the challenge of keeping employment up is higher if economic development allows for mechanization.

Limitations include that this measure is only implemented for crop production, not for livestock production where capital substitution would likely also require a shift in feed mixes. The model is a sectoral model and assumes perfect labor mobility between sectors over longer time-horizons, such that the agricultural workforce can be increased without rising sectoral wage levels. In reality, higher labor demand could drive up wages and lead to a poverty-reducing effect for laborers.

S1.4.3 Biosphere measures

REDD+

The *Biosphere* FSM *REDD+* is implemented in the MAgPIE model.

In REDD+, a carbon price is included in the objective function (cost-minimisation) so that it is less attractive for the model to generate CO2 emissions from land-use change. The carbon price is based on a price trajectory in line with a 1300 Gt CO2 budget, as described in the description of the EnergyTrans measure (S1.4.5), and generated tax revenues are redistributed to citizens (see description of poverty model). The carbon price also incentivises forest restoration, which is driven via a reward on expected, discounted future carbon sequestration[133], for which we assume the same temporal evolution of the carbon density as during the succession of native vegetation. Reforestation is moreover constrained to tropical areas to avoid warming albedo-effects[204] and to areas with a potential carbon density of natural vegetation exceeding 20 t C/ha. This restoration-based approach to reforestation minimizes trade-offs with other environmental targets, such as biodiversity conservation[205].

The measure effectively protects existing forest and other natural vegetation land from conversion and drives carbon-focused land restoration activities. However, the marked constraining effect on agricultural land expansion also drives agricultural intensification via irrigation, shorter crop rotations and cropland relocation to increase area efficiency in production. While the tax burden is not accounted for in the *Production-Factor Use* indicator because tax revenues are used as transfers and not costs, the reallocation of production and the

induced intensification create deadweight losses that increase production costs and poverty. Limitations include that, similar to other supply-side FSMs, we do not simulate any demand-side reaction of diets to the demand-side shock. Food demand is yet relatively inelastic, in particular when considering that elasticity is often measured as the change in food expenditure rather than the change in physical quantities. Much of the elasticity likely occurs in the supply-chain value-added by processing and marketing, and only to a lower degree physical quantities (see SI of Bodirsky et al.[20]). Still, the interaction with food intake is of high relevance, in particular in LIRs where the share of post-farm value-added is lower[50]. Further limitations include that we do not simulate interaction of afforestation with the water cycle[206], include no fertilization requirements for the forest establishment, and simulate no changes in albedo or local climate[207].

LandConservation

The Biosphere FSM LandConservation is implemented in the MAgPIE model. The LandConservation FSM simulates a doubling of protected areas by 2030, such that they rise from currently about 15% to 30% of the global land surface (Supplementary Fig. 2). We assume that the enlargement of protected areas includes both a reactive and proactive component using the conservation priority templates suggested by Brooks et al.[45] as implemented by Kreidenweis et al.[135]. The reactive component focuses on the remaining native vegetation in biodiversity hotspots (BH). Biodiversity hotspots harbor nearly 43% of the world's bird, mammal, reptile and amphibian species and more than half of the world's plant species as endemics, and are characterized by a loss of native habitat of >70%. Biodiversity Hotspots, for instance, include the Atlantic Forest, the Cerrado, the Andes or the Chilean Forest in Latin America; or the Horn of Africa, the Guinean Forests or Madagascar. The proactive component considers large areas (>500 km2) of unprotected intact forest landscapes (IFL) that have so far not been affected by drastic human alteration. IFLs are mainly located in the Amazon and Congo basins in Latin America and Africa and in the boreal zone across North America and Asia.

Protected area enlargement precludes agricultural land expansion by the model in the targeted areas and involves the restoration of land based on pre-industrial conditions (reference year 1750). Direct effects include an improvement of the Biodiversity Intactness Index. Indirect effects are triggered by increased land scarcity, which provides incentives to shift production to unregulated areas within the same world region, to increase imports from other world regions, as well as to intensify production on existing land. This can be achieved via shorter crop rotations, higher nitrogen inputs or irrigation, which could cause trade-offs with croparea diversity, nitrogen surpluses and unsustainable water withdrawals.

Limitations of our modeling include that land conservation within protected areas can in reality be aligned with some degree of food production, including for example extensive grazing or some traditional or other ecosystem management schemes. To some degree, these practices already occur today outside of classified agricultural land and are therefore implicitly covered in the “other land” pool, yet the agricultural outputs from these lands are not explicitly modeled in MAgPIE.

PeatlandRewetting

The Biosphere FSM PeatlandRewetting is implemented in the MAgPIE model.

The initial map for intact and drained peatland (424 Mha and 55 Mha globally, respectively) is based on the Global Peatland Map 2.0[25] and the Global Peatland Database[208], both for the year 2022 (479 Mha in total; ~3.7% of global ice-free land). Future peatland dynamics are estimated by multiplying changes in cropland, pasture and forestry land with a peatland scaling factor[136]. CO_2, N_2O and CH_4 emissions from drained and rewetted peatlands as well as from peat extraction are calculated by multiplication of drained and rewetted peatland area with corresponding GHG emission factors. For boreal and tropical climates, GHG emissions factors are based on IPCC[209] and Wilson[210]. For temperate climates, GHG emissions factors are based on more recent estimates from Tiemeyer et al.[211]. CO_2 emissions from rewetted peatlands are close to zero, i.e. considerably lower compared to drained peatlands. At the same time, CH_4 emissions are higher in rewetted compared to drained peatlands. However, the net effect of peatland rewetting compared to a drained state, on the basis of GWP_{100} equivalents, is a reduction of GHG emissions.

In the FSM PeatlandRewetting, the global AFOLU GHG price is applied as a tax on GHG emissions from peatlands, which penalizes the drainage of intact peatlands and incentivizes the rewetting of drained peatlands. The carbon price is based on a price trajectory in line with a 1300 Gt CO2 budget, as described in the description of the EnergyTrans measure (S1.4.5), and generated tax revenues are redistributed to citizens (see description of poverty model). CH_4 and N_2O emissions are priced by adapting the CO_2 price via GWP100 factors. Costs for peatland rewetting consist of costs for the initial rewetting of 7000 USD per ha and recurring annual costs of 200 USD per ha for maintenance[136].

Rewetted peatlands are assumed to be taken out of production, thereby reducing the cropland, pasture or forestry land, depending on how the drained peatland was used for agriculture or forestry. This increases land competition and leads to relocation of production to other areas, potentially interacting with land expansion, intensification, or increasing agricultural commodity prices. However, impacts on land-use are small compared to other land-related biosphere measures like REDD+ and LandConservation because drained peatland areas account for only ~0.4% of global ice-free land.

Limitations of our modeling include that we do not model alternative uses of rewetted peatlands such as paludiculture or solar panels. The impact of peatland rewetting on water stress only comes from the indirect effects of increasing land scarcity. Direct effects on the water cycle, e.g. by increasing water holding capacity and reducing variability, is not considered. Effects on local climate are also not considered.

WaterConservation

The Biosphere FSM WaterConservation is implemented in the MAgPIE model.

Minimum environmental flows as well as the timing of high flows and low flows are important for freshwater ecosystems as well as riparian vegetation[212], which is why we include the WaterConservation measure as part of the Biosphere package. The WaterConservation FSM refers to a scenario where the protection of environmental flow is enforced. We choose a conservative scenario with minimum flow requirements corresponding to a "fair" conservation status according to Smakthin et al.[137]. For a detailed description of how environmental flow requirements are calculated refer to S.1.1.12. Agricultural water withdrawals that violate the EFR volumes in a given area are prohibited in this scenario.

Water conservation in the form of environmental flow protection can trigger trade-offs with respect to other environmental indicators and inclusion indicators. The direct effect of the

measure is that in water-scarce areas, a part of the irrigated production systems have to shift to rainfed cultivation, eliminating violations of environmental water flows. The reduction of yields in areas that cannot be irrigated for reasons of water conservation trigger indirect systemic effects such as the intensification of rainfed areas, land expansion, irrigation expansion in other areas with abundant water, or increased international trade. All these reactions can increase costs and therefore cause higher food expenditures and poverty. Cropland expansion in turn can have negative effects on the biodiversity intactness both in cropland landscapes and biodiversity hotspots, and causes potentially CO_2 emissions from land-use change. Similarly, land scarcity can create incentives for shorter crop rotations resulting in lower Croparea Diversity.

As a limitation, our modeling does not account for deficit irrigation. In particular in areas where irrigation equipment is already installed, deficit irrigation may be an attractive solution to reduce water withdrawals while increasing water use efficiency on the remaining areas, thereby reducing yield impacts. While the motivation of protection of EFRs is the protection of aquatic and riverine ecosystems, the direct positive effect on biodiversity is not modeled and the effect is therefore not reflected in the BII indicator. Furthermore, the model limitations with respect to modeling water availability and irrigation presented in section S1.1.12 related to the temporal and spatial resolution also affect the outcome of the WaterConservation FSM in that the volume of EFRs is resolution-dependent.

BiodiffOffset

The Biosphere FSM BiodivOffset is implemented in the MAgPIE model. The measure does not allow a net decrease of aggregated BII values in each biome type of each world region[213] as compared to 2020. Any BII reduction (e.g. through cropland expansion) at the biome level must therefore be compensated by increasing the land area of land types with higher BII values (e.g. forest and other natural vegetation). The reference land use (BII = 1) refers to pristine areas with low human influence, while young secondary forest and non-forest vegetation have a lower BII value. Cropland and managed pastures are assigned the lowest BII values[26,28]. The conversion of pristine habitat therefore requires larger compensation areas than the loss of, for instance, young secondary forest.
While being effective in compensation and curbing the loss of intact land, the measure drives agricultural specialization and cropland relocation to increase production efficiency but also has the trade-off of higher production costs, and therefore also food expenditures and poverty.
As a limitation, biodiversity changes resulting from changes in landscape structure, management or pollution levels or the conservation of specific (endangered) species could not be considered here.

S1.4.4 Agriculture measures

NitrogenEfficiency

The NitrogenEfficiency scenario assumes the adoption of technical mitigation measures in croplands and pastures, e.g. improved land-manure application, spreader maintenance, agronomic practices, or the use of nitrification inhibitors. Within the model these changes are implemented through marginal abatement cost curves (MACC) applied to nitrogen emissions. In

NitrogenEff, we use the maximum mitigation rates and their associated costs from Harmsen et al. [98]. Of the three variants ‘optimistic’, ‘default’ and ‘pessimistic’ MAC curves developed in *Harmsen et al.* [98] and available in MAgPIE, we selected the ‘default’ MAC curves. A limitation of this approach is that Harmsen et al. assessed the mitigation potential only with regard to N2O emissions in their analysis. Yet, as the considered mitigation options achieve the reduction of N2O primarily through reducing N surplus, we generalized the mitigation coefficients to apply to the entire nitrogen surplus. Based on the current state of SNUpE, we translated the mitigation rates by Harmsen et al[98] into equivalent proportional changes of the loss rate (1-SNUpEmit) (see section S.1.1.7). Regions with already high SNUpE can therefore reduce their losses less than regions with a low SNUpE. This also leads to a better biophysical consistency as mitigation does not only change emissions but the entire nitrogen cycling, including increasing shares of organic fertilizers when SNUpEmit rises. The technical mitigation costs by Harmsen et al. [98] increase in our model labor and capital demand based on general cost shares in agricultural production.

The direct effect of the measure is a decrease of nitrogen surplus and N2O emissions from croplands and an increase of production costs and Agricultural Labor Demand in agriculture. Increased costs also increase food expenditures and thereby increase poverty.

A limitation of our study is that the individual technical mitigation measures are not implemented on a process-basis into the model, influencing further variables beyond nitrogen surplus and crops. Some measures may for example change crop yields; these effects are partially included in monetarized form within the costs of the measure but do not change the production quantities, and therefore do not include market reaction to changed production quantities.

Nitrogen pollution has several interactions with other outcome indicators. Next to its contribution to climate change via the N2O emissions which is included in our study, nitrogen pollution also reduces biodiversity, increases clean water scarcity or impacts human health via air pollution [214,215]. Increased treatment costs of drinking water may also substantially increase household expenditures[216] and therefore have a poverty impact. These interactions are not modeled in this study.

CropRotations

The CropRotations FSM simulates a tax for the violation of crop rotations, incentivizing short fallow periods, the inclusion of pulses into crop rotations, higher diversity of cereals in crop rotations and lower fractions of cereals within the crop rotation. Moreover, we incentivize that bioenergy crops such as fast-growing trees, oil palms or sugarcane are not cultivated in agglomeration but dispersed (e.g. as agroforestry systems). Similarly, we incentivize the widespread instead of agglomerated cultivation of minor crops such as vegetables and fruits. The parametrization of the tax can be found in Supplementary Data 1. Generated revenues from the tax from the violation of rotational constraints are redistributed to citizens. We assume a distributionally neutral redistribution of tax revenues (broadly similar to a reduction of the value-added tax) but do not include any specific pro-poor redistribution policies (see Soergel et al.[36] for a discussion of their effects).

The direct impact of the measure are more heterogeneous cultivation patterns and therefore a higher Croparea Diversity. As the rotations are hindering the model at placing crops at high-yielding locations, the competition for land increases and incentivizes land expansion, irrigation and intensification, potentially causing trade-offs with other environmental indicators, and increasing production costs.

We do not cover biophysical ecosystem service feedbacks (e.g. on yields, pest control intensity, pollination) which would cause different systemic effects (e.g. via land scarcity, reduced costs).

Landscape Habitats

The LandscapeHabitats FSM restricts cropland area to 80% of the potential cropland area[205] to support native species diversity in cropland landscapes and to sustain a stable supply of critical ecosystem services that underpin land productivity[217]. The area of potential cropland at grid cell level is derived from Zabel et al.[138]. Constraining cropland to 80% of the available cropland is based on evidence that maintaining 20% of other semi-natural vegetation in cropland landscapes promotes resource diversification, connectivity and stability of species populations and supports critical ecosystem services such as pollination, erosion and pest control, as well as climate and air quality regulation[46,205,218–221]. The cropland area requiring relocation in order to fulfill this landscape target at the 1 km level is estimated from high resolution land cover information from the Copernicus Global Land Service[222] and affects approximately 9% of the global cropland area in 2019. The abandonment and relocation of affected cropland areas, including associated shifts in production, are modeled as part of the measure within the MAgPIE model. The measure only marginally raises production costs and incentivises further specialization and increased production efficiency, while considerably improving biodiversity intactness in cropland landscapes. Barriers to implementing the landscape target at the global scale, such as differences in tenure rights, administrative capacity for implementation and monitoring of the measure could not be considered here. In addition we do not explicitly model various land-use options for semi-natural areas, such as extensive grazing, forestry patches, short rotation coppice or dedicated species and ecosystem management activities.

RiceMitigaiton

In the RiceMitigation scenario – similar to the NitrogenEff scenario – technical measures to reduce methane emissions are implemented, including measures such as direct seeding, composting of straw, alternated flooding and draining, as well as improved fertilization. Of the three variants 'optimistic', 'default' and 'pessimistic' MAC curves developed in *Harmsen et al.* [98] and available in MAgPIE, we selected the 'default' MAC curves, but using the most ambitious mitigation measures with the highest mitigation costs.
As methane is a short-term forcer, the resulting reduction of emissions allows to reduce global warming strongly in the short-term. The higher costs are also associated with higher labor requirements.
Our model does not include any demand-reaction such as a substitution of rice by other cereals which could be a consequence of the higher production costs due to rice mitigation. The mitigation measures connected to alternative irrigation regimes are not connected to different water demands, also as it is unclear which proportion of the water requirements of rice are fulfilled using blue water as opposed to rainwater harvesting in paddies.

LivestockManagement

The *LivestockManagement* FSM is implemented in the MAgPIE model. It combines two levers to change livestock production systems, namely a catch-up in the intensification of more extensive ruminant production systems to reduce overall feed and land requirements, and technical mitigation measures to reduce CH_4 emissions from enteric fermentation.

We assume livestock productivity increases beyond those projected for our baseline scenario, with a focus on ruminant production systems and regions with comparatively low productivity levels. As livestock production has been shown to grow much faster than both arable land used for feed cultivation and pasture during the second half of the 20th century[223], disregarding livestock productivity increases in the baseline scenario would lead to an overestimation of future land requirements from the expected continuing nutrition transition and the related shift from plant-based to animal-source foods. In the baseline, aggregate conversion of feed into edible products from ruminant production systems improves from 35 to 32 on gross energy basis between 2020 and 2050 (defined as gross energy content of feed use divided by the gross energy content of products), even though demand for beef and milk is projected to increase more strongly in regions with extensive livestock production.

The LivestockManagement FSM assumes a catch-up in the intensification of ruminant production systems in regions which currently have low productivity levels, leading to an aggregate feed use per unit product of 25 (gross energy basis) in 2050. The catch-up is most pronounced in SSA where feed conversion improves from feed use per unit product of 63 (gross energy basis) in the baseline to 36 in LivestockManagement, while in EUR the feed use per product remains in both scenarios at 13 (gross energy basis). The LivestockManagement FSM modifies both the level of feed demand, but also the feed composition[94,95], since productivity gains go along with a shift from feed sources with a low nutrient content such as grazed biomass or crop residues to feed types with a higher nutrient content cultivated on cropland[154,224]. CH_4 emissions, which are calculated based on the Tier-2 approach of the IPCC guidelines for national Greenhouse Gas inventories[172] are calculated on the basis of the feedmix and fall with higher feed efficiencies.

In addition, technical mitigation measures are implemented in MAgPIE using the marginal abatement cost (MACCs) from *Harmsen et al.* [98], which were drawn from an extensive review of mitigation options found in the literature. Of the three variants 'optimistic', 'default' and 'pessimistic' MACCs developed in *Harmsen et al.* [98] and available in MAgPIE, we selected the 'default' MACCs and the respective levels of additional production costs required to exploit the full 'default' mitigation potential to parametrize this FSM. This set-up includes the measures of "addition of nitrate to the feed", "genetic selection and breeding", "adding tannins as a food supplement", "grain processing" and "improved health monitoring and illness prevention", were the ranges refer to reduction efficiencies, see Table 1 in *Harmsen et al.* [98]. The measure "Seaweed (Asparagopsis taxiformis)" is only included in the *optimistic* variant of the MAC curves, and therefore not part of the *LivestockManagement* FSM.

The direct effects of the measure are a decrease in total feed demand, especially for pasture-based biomass and crop residues, while feed demand for crops and processing-byproducts increases. This is reflected by a reduction of pasture and a higher cropland area compared to the baseline, as well as lower nitrogen surplus from pastures and higher surplus from cropland soils and animal waste management systems. CH_4 emissions are strongly reduced. Production-Factor Use and Agricultural Labor Demand in agriculture increase, which also drives food expenditures.

Limitations of this measure include that the development of feed basket composition over time is prescribed exogenously for the different production systems based on productivity assumptions and based on the feed baskets at the start of the simulation period. The model cannot endogenously respond to the scarcity of individual feed types, e.g. it cannot decide as part of the cost-optimisation to switch between similar feed sources. Therefore, the feed mix is not cost-optimal. Another limitation is that grazed biomass is a homogenous product in the model and improvements in pasture management can therefore not be modeled as a potential measure to improve livestock productivity. Moreover, technical measures to reduce CH_4 emissions from enteric fermentation are only captured via abatement costs and not on a process basis, therefore not affecting mass flows beyond some minor cost-induced trade adjustments.

ManureManagement

The ManureManagement FSM is implemented in the MAgPIE model.
In the *ManureManagement* scenario, we firstly assume for nitrogen that 50% of the manure that is excreted in confinements is going into anaerobic digesters with 90% N recycling rate, while the remaining manure is managed according to historical fractions[56,172]. Manure excreted on pastures is not affected. Secondly, CH_4 emissions from manure management are reduced by applying technical mitigation measures as proposed by Harmsen et al. [98], including measures like reduced storage times, anaerobic digesters, manure acidification, or installing covers over storages. We here use the maximum mitigation rates and their associated costs from Harmsen et al. [98]. Of the three variants 'optimistic', 'default' and 'pessimistic' MAC curves developed in *Harmsen et al.* [98] and available in MAgPIE, we selected the 'default' MAC curves.
The direct effect of *ManureManagement* is a reduction in N surplus from manure management and higher recycling rates, resulting in a replacement of inorganic fertilizers by recycled manure from confinements, saving also some costs for fertilizers. N_2O and CH_4 costs are directly reduced, and Production-Factor use and Agricultural Labor Demand increase due to the implementation costs. Second order effects include a rise of agricultural commodity prices and price-induced poverty due to rising production costs.

Limitations include that we use two different approaches for nitrogen and methane which are not fully congruent, as for methane a wider range of measures is included. In line with the methodology used in the NitrogenEff scenario, the same health, inclusivity, and environmental limitations apply.

SoilCarbon

The FSMs SoilCarbon is implemented in the MAgPIE model. Within this measure soil organic carbon (SOC) degradation is disincentivized and SOC sequestration is incentivized through a carbon price on SOC (including litter layer). The carbon price is based on a price trajectory in line with a 1300 Gt CO2 budget, as described in the description of the EnergyTrans measure (S1.4.5), and generated tax revenues are redistributed to citizens (see description of poverty model).

To track decline or built-up of SOC, we use the IPCC tier 1 approach of the IPCC guidelines for national greenhouse gas inventories[169,172]. Here, stocks are calculated via a reference SOC stock under natural vegetation and stock change factors given by the IPCC for the topsoil (0–30 cm) and based on observational data. Estimates of stock change factors are for this study differentiated by crop type, irrigation regime and climatic zones. The reference SOC stocks are based on topsoil SOC estimates for potential natural vegetation from LPJmL[32]. The subsoil SOC is assumed to be not affected by land-use change and land management practices. All land-use types besides cropland are modeled as undisturbed, following the idea that grasslands and managed forest areas are rather similar to potential natural stocks[225]. Assuming a fixed C:N ratio in cropland soils, building up SOC requires nitrogen while depletion of SOC makes nitrogen plant-available.

Pricing SOC emission and incentivizing SOC gains leads to an overall reduction of emissions from the whole AFOLU sector, as production patterns are optimized for emission reduction. The model has an incentive not to place cropland on SOC rich soils, and to reduce land conversion as this leads to a steep decline in SOC. As more products need to be produced on potentially smaller and less fertile soils, the model has an incentive to intensify the remaining areas through endogenous yield improvements, shorter crop rotations or irrigation expansion. For the latter we also assume a positive impact on SOC compared to the unirrigated system[226]. As the pricing changes the land allocation compared to the previous cost-optimal state, production cost as well as *Expenditures for Agricultural Products* increase as an indirect effect. Other indirect effects include that the higher incentives for irrigation can lead to higher environmental flow violations, and that cropland becomes more agglomerated in areas where SOC losses through management are not so high.

Our approach does not consider that rising residue and manure recycling could increase SOC on croplands. We also do not consider yield benefits arising from higher SOC[227].

S1.4.5 Cross-Sector measures

Population

The Population Cross-Sector measure is changing the settings in the MAgPIE model, poverty model and dietary health model.

In the Population scenario we switch the population, demographic structure, and urbanization patterns from SSP2 to SSP1, which includes a relatively lower global population, stemming from

lower fertility rates across all world regions. Global population in the year 2050 is reduced to 8.9 billion people. In line with the population growth, built-up land projections change from SSP2 to SSP1 based on Hurtt et al. [139].
As population is one of the main drivers of the MAgPIE model, this leads to slightly lower demand for agricultural commodities, lower pressure on environmental systems, lower total production costs, and lower agricultural commodity prices. The lower total population also reduces the absolute number of underweight and obese individuals. However, as this population with SSP1 demographic patterns has an older population, the population is also more vulnerable to diet-related diseases.
As a limitation, the Population and HumanDevelop measures are modeled as independent drivers, while in reality they would strongly interact bidirectionally. While this allows decomposing the effects, both measures are only consistent when activated together, e.g. in the CrossSector package. As REMIND only simulates Population and HumanDevelop together, we didn't have consistent emission scenarios for all economic sectors available and grayed out the temperature effects in Figure 3. The bioenergy demand in this scenario stays the same as in $SSP2_{BASE}$.

Human Develop

The *HumanDevelop* Cross-Sector measure is changing the settings in the MAgPIE model, REMIND model, the dietary health model and the poverty model.
In the *HumanDevelop* scenario that includes a more equal economic development and better functioning and more inclusive institutions, we assume a switch from SSP2 to SSP1 for the settings of per-capita income[228], as well as reduced risk premiums investments leading to a reduction of discount rates in low income countries from 10% to 6%[197] and dietary preferences using a different functional form for the demand regressions[2], leading to lower food waste, and lower consumption of animal products and empty calories in HICs (see also section 1.4) . For consistency with a faster general technological development and a technological catch-up between world-regions, we also assume a higher $SNUPE_{base}$ development (see section S.1.4). In line with healthier lifestyles, physical activity levels in countries with sedentary lifestyles are changed to moderate activity[2]. In the poverty model, the switch of GDP from SSP2 to SSP1 also goes along with a different projection of intra-national inequality, which is parametrized through the Gini projections by Rao et al.[123].

Higher economic development, especially for lower-income countries, accelerates the nutrition transition and increases food demand, especially for livestock products. At the same time, more sustainable dietary preferences modestly reduce the consumption of livestock products in HICs. Higher incomes also reduce the prevalence of underweight in low-income countries faster, and lead to a faster rise in obesity. Increased physical activity levels further increase global food requirements by roughly 6%[2], mainly in middle and high-income regions.
This increased food demand drives the need for increased agricultural production, by expanding and/or intensifying land. In this scenario, investment in yield-increasing is less costly in lower-income regions due to the lowered interest rates. Despite these improvements, the model may still expand agricultural lands to meet food demand.
The limitations discussed above for the Population scenario also hold for the HumanDevelop scenario.

EnergyTrans

The CrossSector measure EnergyTrans is changing settings in the REMIND model and the MAgPIE model. This section describes the REMIND model methodology as it was applied in Strefler et al[124,125] and Soergel et al.[26], and is therefore an abridged version from the description in these papers.

In the EnergyTrans measure, we assume a robust energy transformation in line with the SSP2 900Gt scenario of Soergel et al.[26] in the REMIND model. In this study, we use a consistent set of non-AFOLU emissions and bioenergy demand from this scenario. In this scenario, a carbon budget of 900 Gt CO_2 from 2011 (corresponding to around 410 Gt CO_2 from 2023) until the time of net-zero CO2 emissions (approximately peak warming) is not exceeded, in line with restricting warming to 1.5°C with a small overshoot. The carbon prices are adapted as part of the REMIND optimisation to limit global cumulative CO_2 emissions in line with the carbon budget. The scenarios by Soergel et al.[26] introduce a carbon price in 2025, but at different regional price levels differentiated by regional per-capita income levels. The carbon prices in high-income regions increase linearly until the carbon budget is reached, while lower-income regions initially face substantially lower prices. After the carbon budget has been reached, the CO_2 price increase flattens off to US$3 per ton CO_2 per yr, which is sufficient to ensure that the GMT increase declines from its peak to values consistent with the 1.5 °C target with at least 67% probability by the end of the century. Non-CO_2 GHGs are priced according to their 100-yr global warming potentials (using IPCC Fifth Assessment Report values).

The EnergyTrans measure triggers several dynamics in the models. In the energy system, the CO_2 price induces higher energy efficiency, a shift in electricity generation from fossil to renewable energy, and a replacement of fossil fuels in the three main energy demand sectors (transportation, residential & commercial buildings, industry). Electrification is by far the largest contributor to decarbonising the demand sectors, with smaller roles played by hydrogen and second-generation bioenergy (the latter mainly in the form of liquid biofuels). Compared to other technologies, bioenergy also provides the option to remove CO_2 from the atmosphere which can be captured after burning and stored, even when used for liquid biofuels[229].

Cumulative emissions of the non-AFOLU sectors in the period 2020-2050 fall from 1020 Gt CO_2 in the $BASE_{SSP2}$ to 521 Gt CO_2 in the EnergyTrans scenario, shifting the global-warming trajectory from RCP6.0 to RCP2.6. 2nd generation cellulosic biomass demand increases from 7 EJ in 2050 in the baseline case to 84 EJ in the EnergyTrans scenario.

From this scenario, which assumes mitigation in the entire economy, we extract a consistent set of non-food system emissions as well as the bioenergy demand. If the EnergyTrans measure is activated in isolation, the non-food system emissions as well as the bioenergy demand are consistent with this 900 Gt budget, while we assume no mitigation in the food system beyond the NPIs and NDCs that are also active in the BAU_{SSP2} (see section S1.3). The EnergyTrans measure only affects the land system via an increase in bioenergy demand, a reduction of global warming, as well as a reduced non-agricultural water demand. The latter is simulated by retaining a reduced quantity of surface water for energy, industry and residential use. The quantities are based on trajectories estimated by the WaterGAP model[141], switching from the SSP2 trajectory in BAU_{SSP2} to the SSP1 trajectory when EnergyTrans is activated. According to these trajectories, domestic water use intensity in SSP1 is 20% lower than in an SSP2 scenario by 2050 because of behavioral changes. Both the energy and the manufacturing sector experience higher technological change rates in an SSP1 compared to an SSP2 scenario and better cooling technologies for power plants in SSP1.

Direct effects include an increase of the value of the *Bioeconomy Supply* indicator, as well as an increase of *Production-Factor Use* and *Agricultural Labor Demand* for the production of bioenergy crops. Indirect effects are triggered by the increasing competition for land and water caused by the additional bioenergy supply. The *EnergyTrans* scenario does not include any accompanying measures in the land system which would reduce the environmental impacts of bioenergy, such as such as carbon-pricing or land protection. The cultivation of bioenergy therefore causes an intensification of existing croplands as well as an expansion into semi-natural land, reducing the *Biodiversity Intactness Index* as well as the *Croparea Diversity*, and increasing *Nitrogen Surplus*. Even though bioenergy crops are not irrigated in our model, and even though non-agricultural water demand is falling, the intensification of croplands for other crops is partially achieved through irrigation expansion, causing higher violation of environmental water flows.

If the EnergyTrans measure is activated as part of the *CrossSector* and FST_{SDP} scenarios, which simulate a world that is not only engaged in mitigation but also in sustainable development more generally, we use a projection of the Sustainable Development Pathway (SDP)[26]. While the SDP pathway from this study employed a 1000 Gt budget, here we choose an otherwise identical setup but a 900 Gt budget to stay comparable to the EnergyTrans scenario. This scenario achieves the same mitigation target within the context of broader shift towards sustainable development. This includes lower population growth, faster expansion of the education system, more equitable GDP growth (in line with our Population and HumanDevelop measures), lower final energy demand (especially in HIRs) due to both efficiency measures and a shift towards more sustainable lifestyles, and a number of energy-system sustainability measures (e.g. phase-out of coal, phase-out of traditional biomass for cooking and heating, electric vehicle mandate, restrictions to bioenergy and CCS use; see Soergel et al.[26]). From this scenario, we again use the non-food system emissions and the bioenergy demand, resulting in a demand for 2nd generation cellulosic biomass of 57 EJ by 2050, and cumulative CO2 emissions of 512 Gt CO_2 from the non-AFOLU sectors in the period 2020-2050.

Limitations in the model implementation that affect the land system include that we do not simulate the land requirements for other energy carriers such as solar-PV, wind or coal within the land-use system which could create further competition for land (van de Ven et al 2021). While the linkage to the LPJmL model takes into account that mitigation reduces the impacts on crop yields and changes the water availability, we do not consider a broader set of climate impacts. Within the food system, this includes for example a rising risks for extreme events and maladaptation[230], as well as potential impacts of heat on labor productivity[62]. We also do not account for the reduction of climate impacts on the macroeconomy[231], or directly on biodiversity in natural systems[16].

We use a different, lower carbon price trajectory (based on an SSP2 scenario with a carbon budget of 1300 Gt CO2, also using the model setup of Soergel et al. [26]) for the food system FSMs that require a carbon price, REDD+, SoilCarbon, and PeatlandConservation, in order to avoid unnecessarily high tax payments and food price changes at a tax rate where mitigation is saturating. As such, the two different tax rates (185USD_{05MER}/tCO_2 in AFOLU and 494USD_{05MER}/tCO_2 in other sectors in the year 2050 in the FST_{SDP} scenario) diverge from the theoretical allocation optimum of a uniform carbon price; as we estimate global warming ex-post and keep consistency between the energy-scenario and bioenergy demand, our scenario remains biophysically fully consistent.

Bioplastics

The Bioplastics FSM is implemented in the MAgPIE model. It assumes that 30% of the projected plastic demand by 2050 is replaced by bioplastics.
Total plastic demand is assumed to increase to 675 Mt[142]. Demand for bioplastics leads to an increased material demand from agricultural products, as the production of bioplastics requires biomass in the form of oils, sugar, starch, glycerol, or cellulose (see section S1.1.2).
The increase in demand for agricultural products increases overall production costs in agriculture. The area and intensification requirements for the production of additional materials can create trade-offs with biodiversity, nitrogen pollution and environmental water flows.
We did not account for potential efficiency improvements in bioplastics production, but assumed a constant average conversion factor from biomass to bioplastic is used for all substrate sources[150]. The composition between different biomass sources for bioplastic production is assumed to stay constant based on current shares[150]. Glycerol is a byproduct from other production and therefore assumed to be available as substrate for bioplastic production without additional agricultural production and not modeled explicitly.

TimberCities

The "TimberCities" cross-sector measure is implemented in the forestry module of the MAgPIE model. In the TimberCities measure, we assume an increase in global demand for engineered wood. Demand for engineered wood to be used in construction of mid-rise urban buildings is derived as a function of the new influx in urban population newly moving into cities, woody biomass demand per capita and amount of peak urban population expected to live in cities[143,232]. We assume that by the end of this century, 50% of people newly moving into cities after 2020 could be housed in buildings made of engineered wood rather than conventional cement and steel structures.

The direct effects of the TimberCities Cross-Sector measure is an increasing demand for industrial roundwood that leads to an increased biomass harvest from both forest plantations and natural forests as well as to an increased establishment of forest plantations to meet increasing demand for engineered wood. The clearing of natural forests and the establishment of plantations in semi-natural areas reduces BII. Using engineered for construction leads to an additional pool of long term carbon storage in harvested wood products. Also, harvest of forests and establishment of plantations are connected to additional production costs.
Indirect effects are caused via the land-use and water-use competition. The increasing competition and scarcity on land markets leads to an intensification of production with shorter crop rotations, as well as rising food expenditures as poverty risk.

The following dynamics are not represented in the model:
TimberCities only targets new buildings, but also the renovation or rebuilding of existing building infrastructure could be wood-based. The growth curves for forest biomass do not include the effects of CO2 fertilization and impacts of climate change. Rotation lengths in plantations do not take into account changing industrial roundwood prices due to increase in demand for engineered wood. We assume no additional nitrogen fertilization needs for the establishment of plantations. The global warming potential does not include albedo effects caused by changes in land cover through forest plantations and loss in natural forest cover. Agricultural Labor Demand does not consider the jobs created within forestry. The predominant

choice for industrial roundwood processing is softwood, primarily due to its material properties and the fact that the machinery used in processing wood for construction is primarily designed for softwood rather than hardwood. MAgPIE does not differentiate between hardwood and softwood, and assumes that engineered wood is produced from both sources, which would require building up new production facilities to handle hardwood.

S2: Supplementary Results

S2.1: Validation and model intercomparison

Historical data for our core outcome indicators (Fig. 2) were derived as follows. Agricultural products expenditures were estimated for each product by multiplying FAO food demand with a global producer price (averaged by demand). The poverty headcount of people living with less than 3$20 per day was from the World Bank database[233], which contains country-level data from 1967 to 2020. The downloaded version still did not include the COVID impacts on poverty in the year 2020, which explains why our 5-yearly estimates diverge from the trend. To derive a global dataset from World Bank[233], missing data were filled either by linearly interpolating existing data for the specific country or, where interpolation was not possible, by using average regional shares of the population living under the poverty line. Agricultural Labor Demand is based on a dataset by ILOSTAT reporting aggregated employment in agriculture, forestry, and fishery, which was disaggregated based on employment shares from a more detailed but incomplete ILOSTAT dataset[160]. Agricultural wages are calculated by dividing total labor costs in agricultural production (based on value of production data by FAOSTAT[146], and labor cost shares by USDA[159]) by total hours worked (based on agricultural employment and average weekly working hours[160]). Gaps in the resulting dataset were filled based on a regression between hourly labor costs in agriculture[160] and per-capita income. The historical BII estimate is based on Phillips et al.[234] and differs from our estimate due to a lower estimate of forests in their land use patterns (see also Fig. S10 d,e). Historical *Croparea Diversity* was estimated by applying the Shannon Index on the historical landuse patterns from Ostberg et al.[235].
Historic nitrogen surpluses were estimated based on the same method as in the MAgPIE model, yet using historical activity numbers for crop and livestock production, land areas, atmospheric deposition, and fertilizer consumption. Cumulative greenhouse gas emissions are based on FAOSTAT[236]. Global surface warming is based on the IPCC AR6 Summary for Policymakers Global Surface Temperature Anomalies (GSTA) time series relative to 1850-1900, which uses the observations of HadCRUT4.6[65].
Supplementary Fig. 3-Supplementary Fig. 15 provide further central model indicators and compares them to historical trends.
The involved models are well-established in the literature. Supplementary Table 6 lists previous studies of these models in which results have been validated or compared to other models.

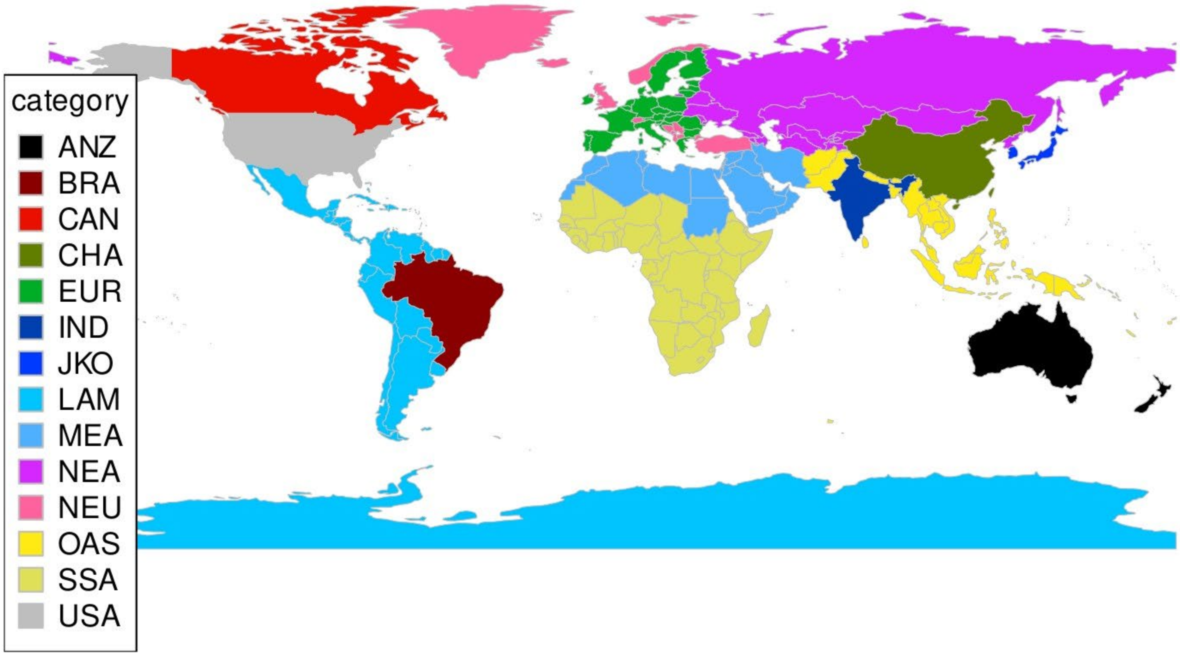

Supplementary Fig. 1

Map with world regions used for the MAgPIE simulations in this assessment. Regional abbreviations are in the Supplementary Table 2.

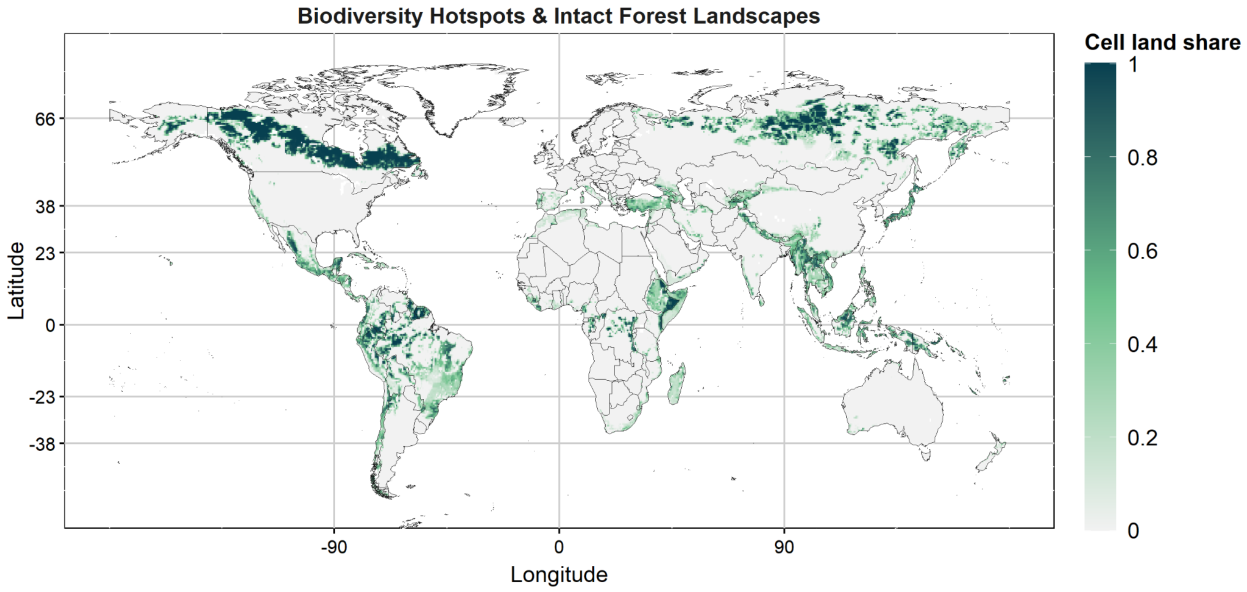

Supplementary Fig. 2

Biodiversity Hotspots & Intact Forest Landscape areas covered by the "LandConservation" food system measure.

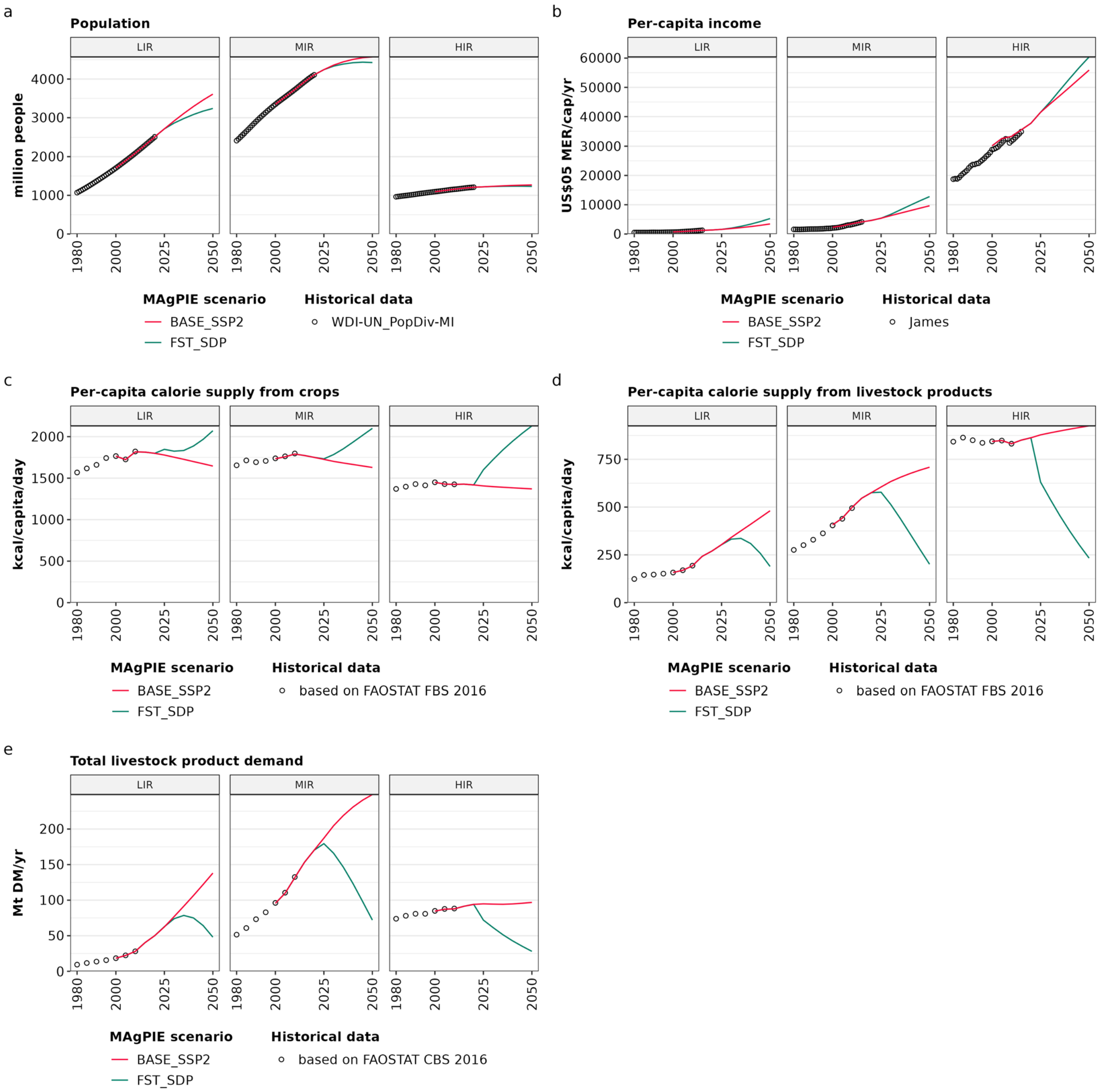

Supplementary Fig. 3

Comparison of population, per-capita income, per-capita calories supply from crops and livestock products, and total livestock product demand with historical trends from the UN WDI[237], James et al[238], and FAOSTAT Food Balance Sheets and Commodity Balance Sheets[146] for current low-income, middle-income, and high-income regions (see S1.1.1).

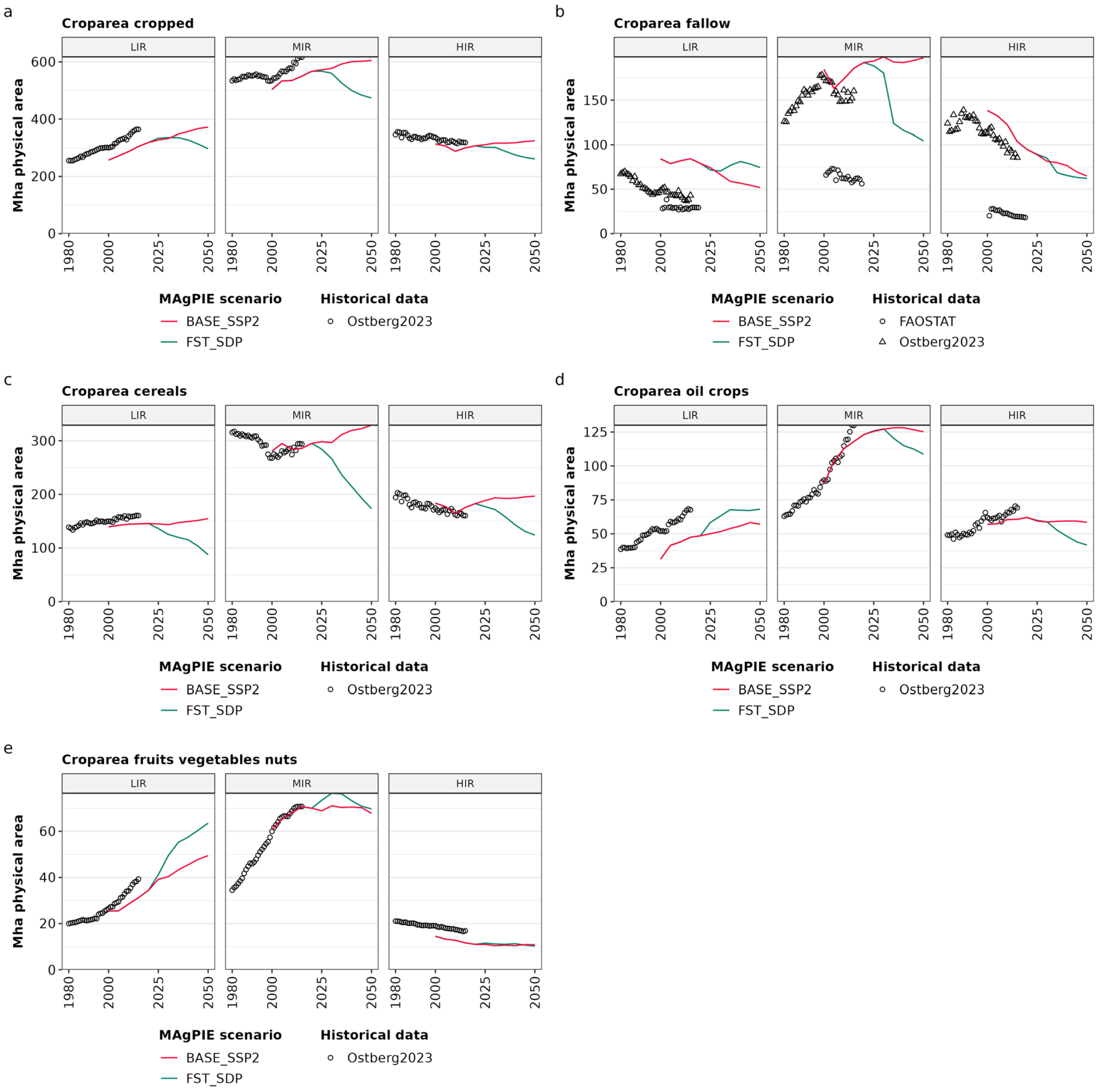

Supplementary Fig. 4

Comparison of physical croparea under cultivation (double-cropped areas count only once, fallow is excluded), croparea under fallow, and croparea under cereal, oil crop or fruits, vegetables and nuts cultivation with historical trends from Ostberg et al.[235], and FAOSTAT Land Use [239] for current low-income, middle-income, and high-income regions (see S1.1.1).

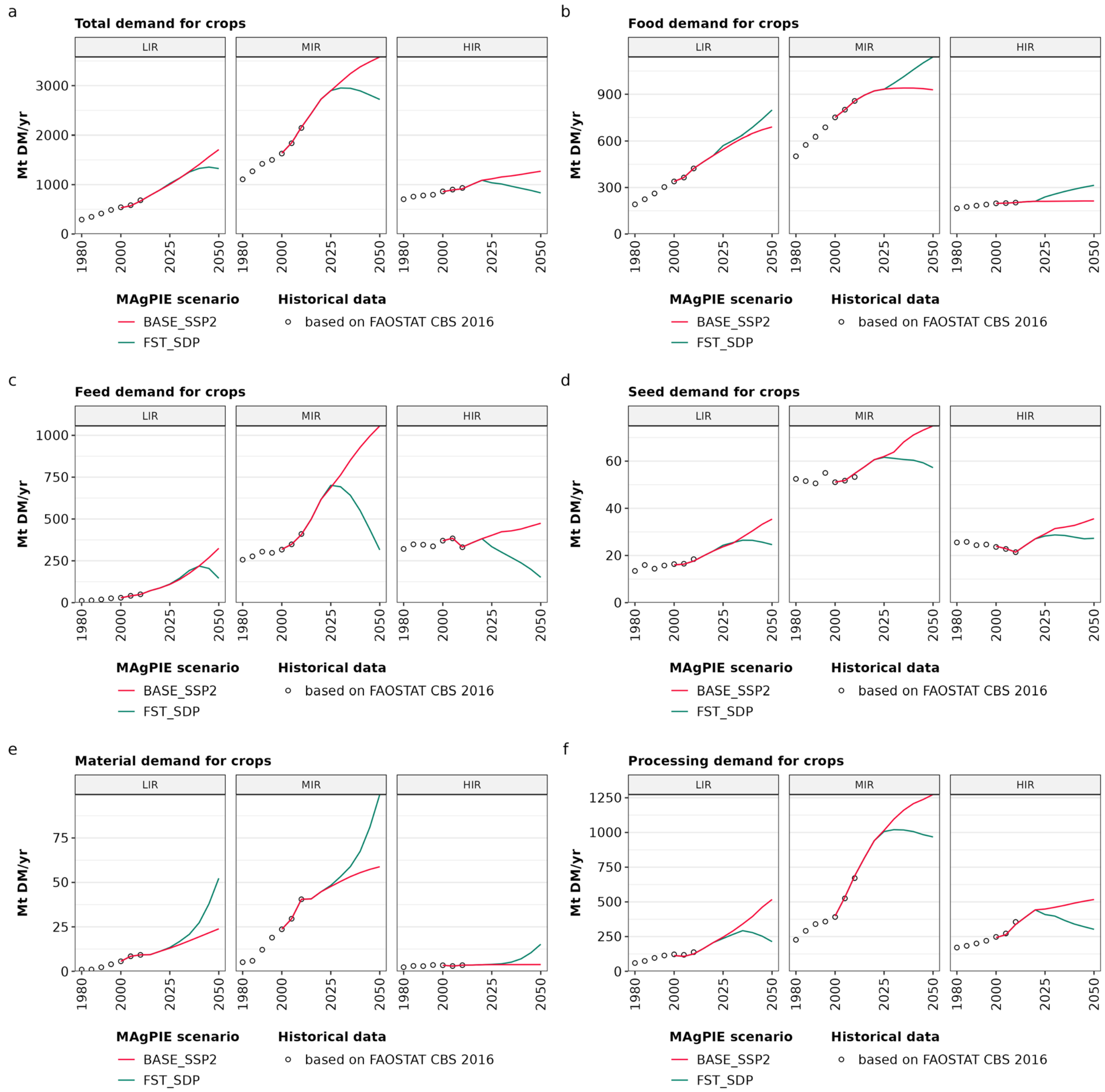

Supplementary Fig. 5

Comparison of various demand categories for crops with historical trends from FAOSTAT Commodity Balance Sheets[146] for current low-income, middle-income, and high-income regions (see S1.1.1). Demand categories are based on FAOSTAT use categories, yet assigning crops for ethanol production to Processing demand instead of Material demand for both model outputs and validation data.

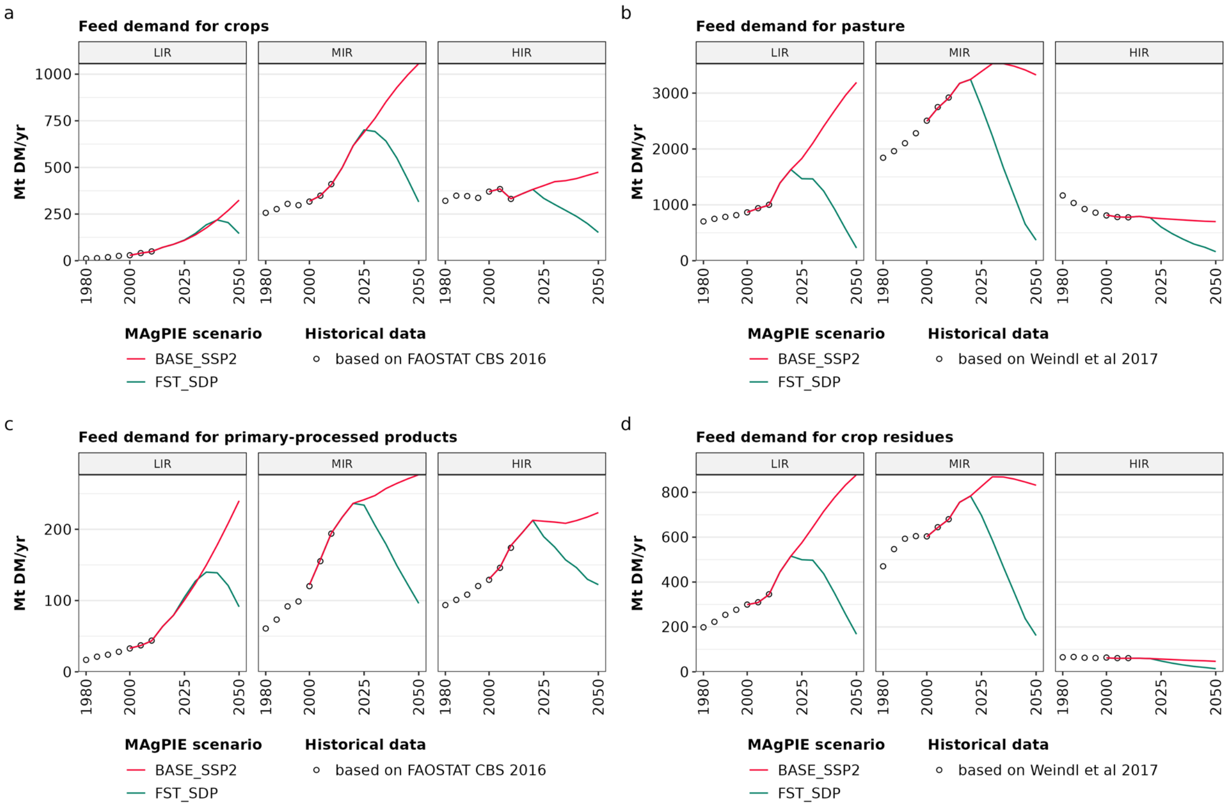

Supplementary Fig. 6

Comparison of feed demand for crops, pasture, primary-processed products (oilcakes, molasses, brans) and crop residues with historical data from FAOSTAT CBS[146] and FAOSTAT derived data using the feed model of Weindl et al.[95] for current low-income, middle-income, and high-income regions (see S1.1.1).

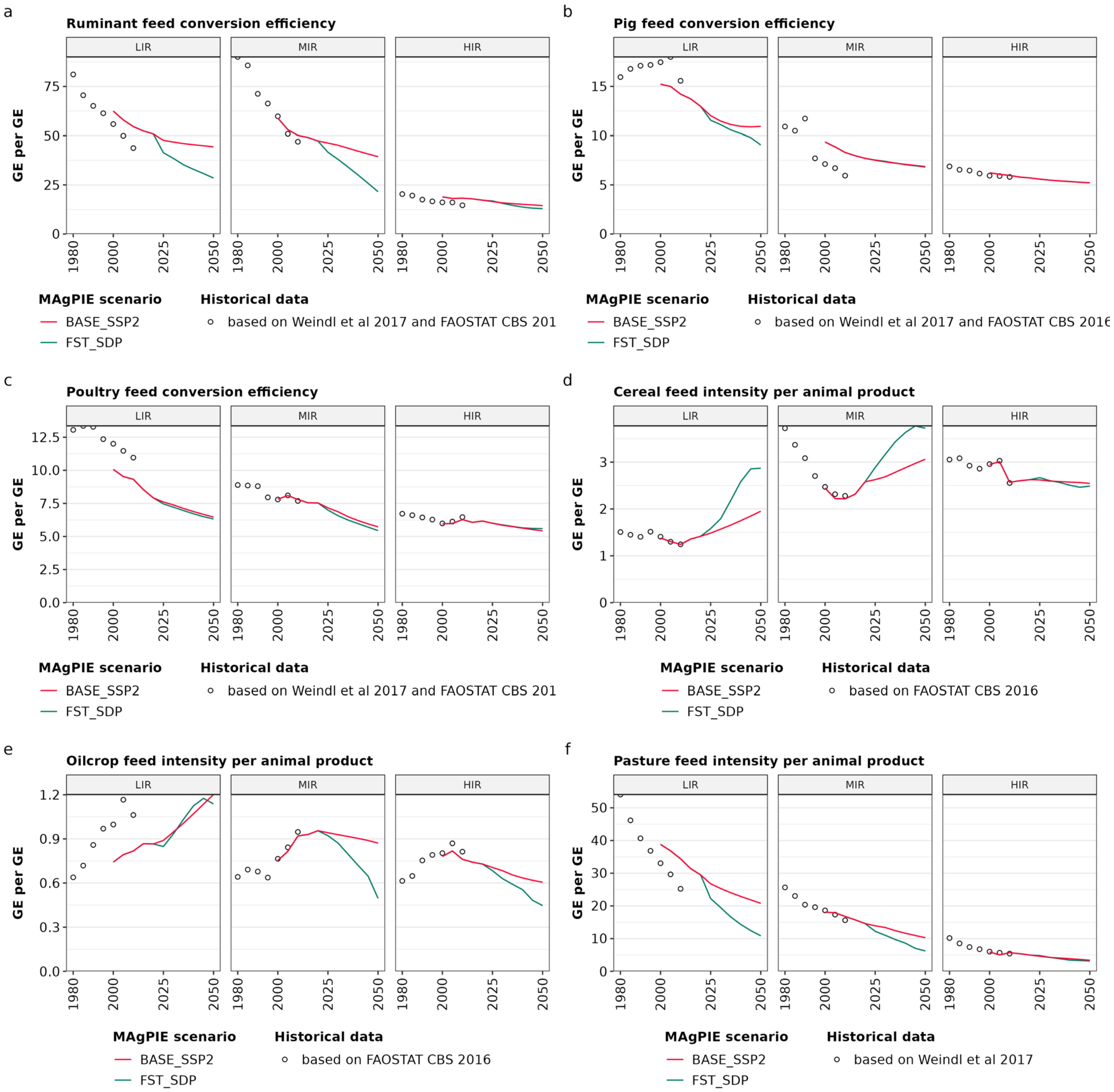

Supplementary Fig. 7

Comparison of feed conversion efficiency of ruminant meat and milk (a) pig meat (b), poultry meat and eggs (c), cereal feed intensity over all animal products (d), oilcrop feed intensity over all animal products (e), and pasture feed intensity over all animal products (f) with historical trends from FAOSTAT Commodity Balance Sheets[146], and feed baskets estimated based on FAOSTAT using the methodology of Weindl et al.[95]. Estimates are plotted for current low-income, middle-income, and high-income regions (see S1.1.1).

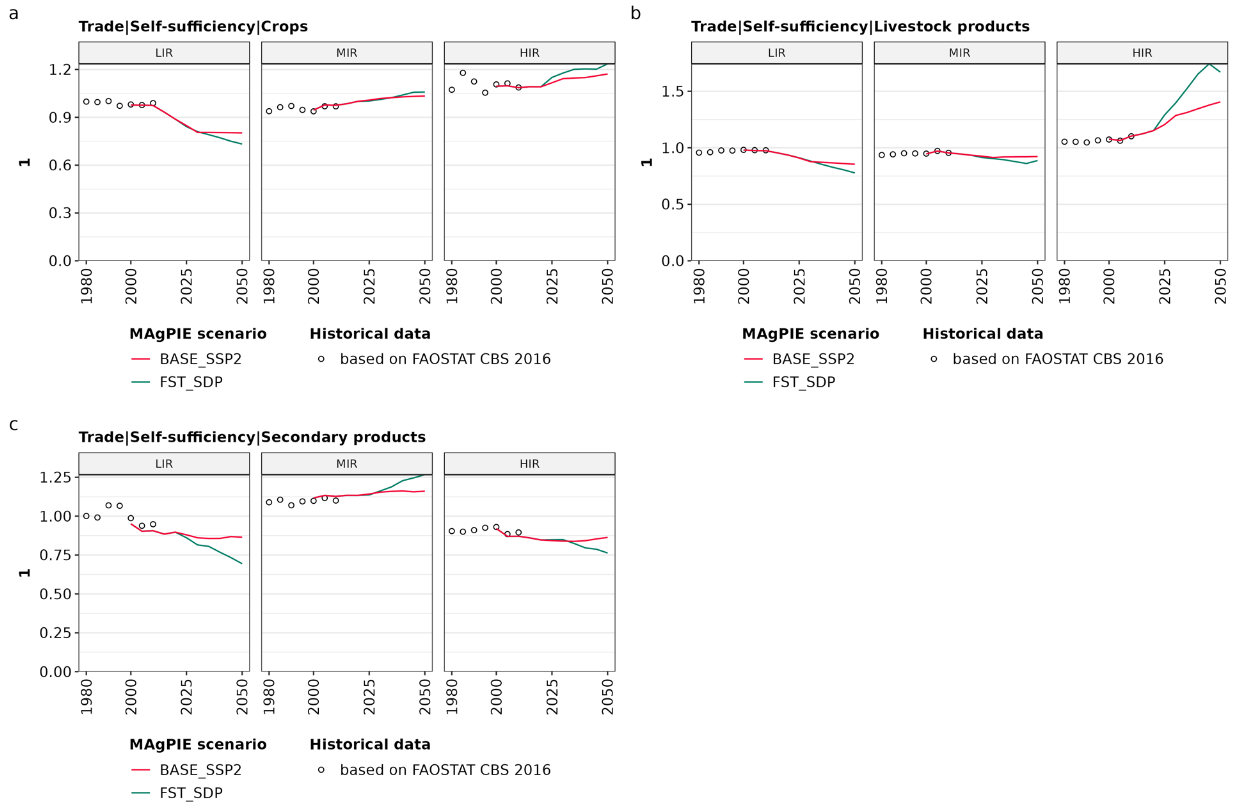

Supplementary Fig. 8

Comparison of self-sufficiencies (Production / Demand) for Crops, Livestock Products and Primary-Processed Products (oils, sugar, alcohol, ethanol and byproducts) with historical trends from FAOSTAT Commodity Balance Sheets[146]. Estimates are plotted for current low-income, middle-income, and high-income regions (see S1.1.1).

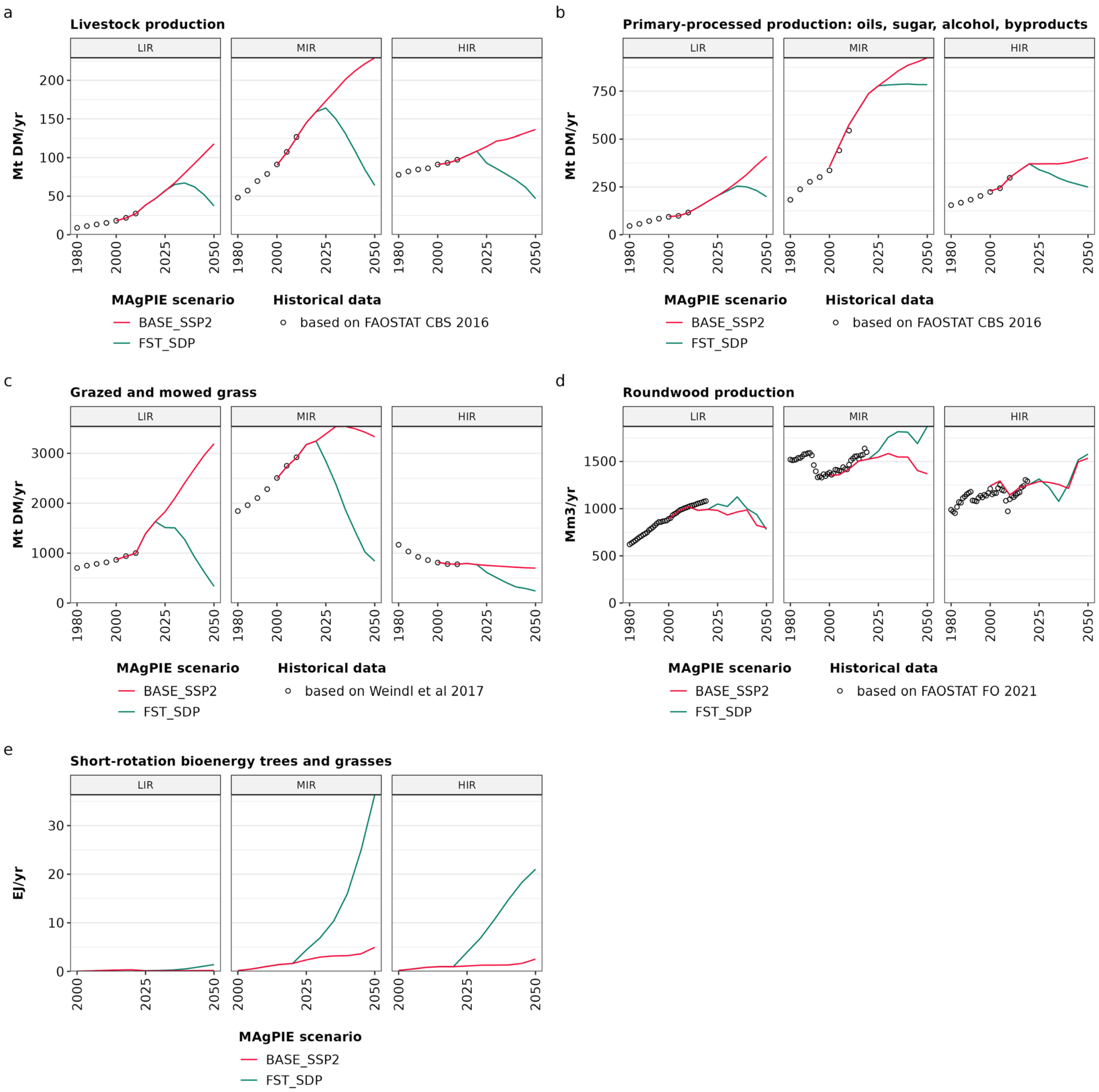

Supplementary Fig. 9

Comparison of production of livestock products (a), oils, sugar, alcohol, ethanol and byproducts (b), grazed and mowed grass (c) , roundwood (d) and short-rotation cellulosic grasses and trees for bioenergy (e) with historical trends from FAOSTAT Commodity Balance Sheets[146], grazed biomass based on method by Weindl et al[95], FAOSTAT Forestry Production and Trade[240], for current low-income, middle-income, and high-income regions (see S1.1.1).

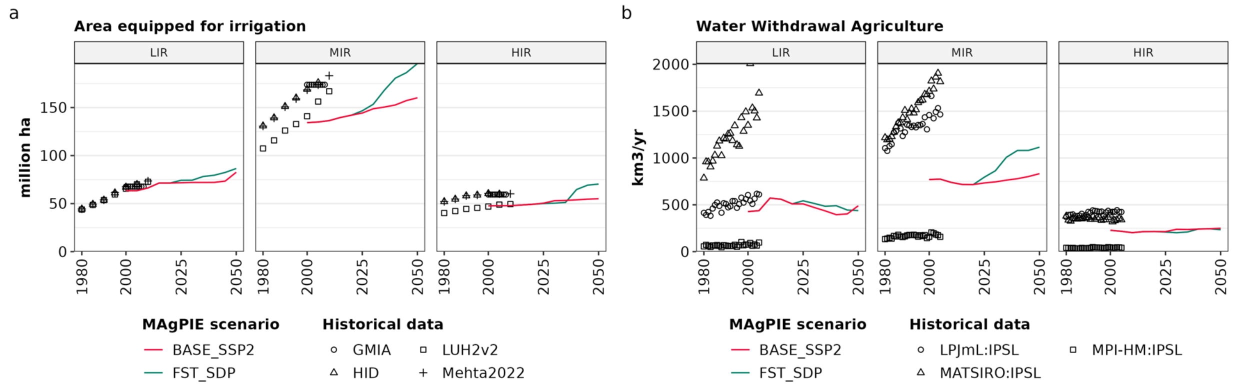

Supplementary Fig. 10

Comparison of area equipped for irrigation with historical trends from Siebert et al. (GMIA) [241], Hurtt et al. (LUH)[139], Siebert et al. (HID)[242], Mehta et al.[243] (a); and agricultural water withdrawals with historical trends from the LPJmL, MATSIRO and MPI-HM models extracted from ISIMIP2b[244] (b), for current low-income (LIR), middle-income (MIR), and high-income regions (HIR) (see S1.1.1).

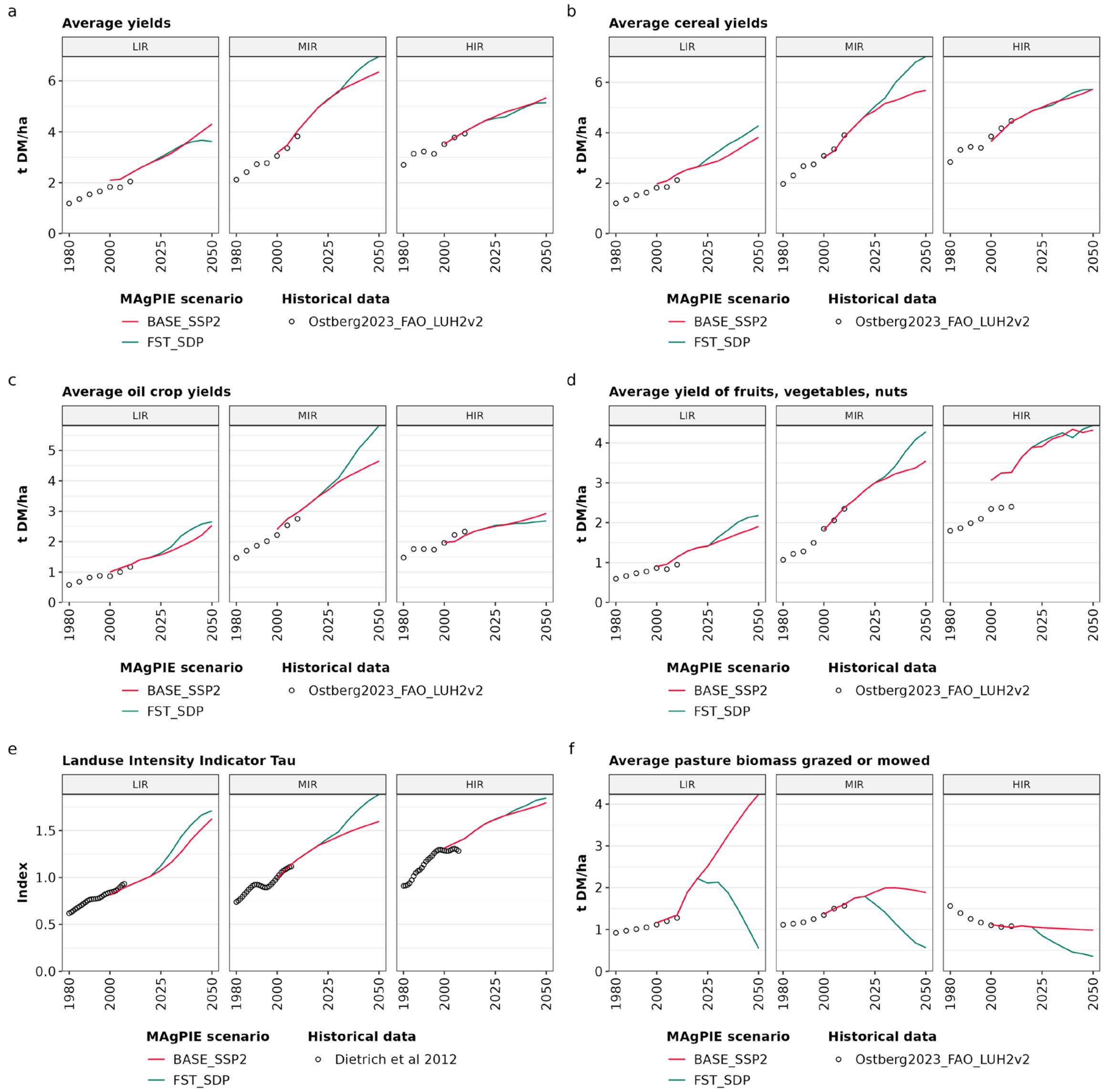

Supplementary Fig. 11

Comparison of crop yields for an all-crop-average (a), cereals (b), oil crops (c), fruits vegetables and nuts (d), as well as the Landuse Intensity (e), and the average biomass grazed or mowed (e). All yields are per physical area, including double cropping and excluding fallow land. Historical data for yields divides FAOSTAT CBS production[146] by physical area estimates by Ostberg et al.[235] which were harmonized with total croparea by LUH2v2[139]. Historical trends for Landuse Intensity is based on Dietrich et al[245]. Pasture biomass grazed or mowed is based on estimates of pasture feed requirements using the historical FAOSTAT data and the methodology of Weindl et al[95] divided by the pasture area by LUH2v2[139]. In India (part of LIR), pasture feed requirements are very high for the estimated pasture areas, leading to very high yields; this is likely caused by poor data quality for feed, and by occasional grazing and feeding of cows on biomass from non-pasture areas[154,224]. Comparison is done separated by current low-income, middle-income, and high-income regions (see S1.1.1).

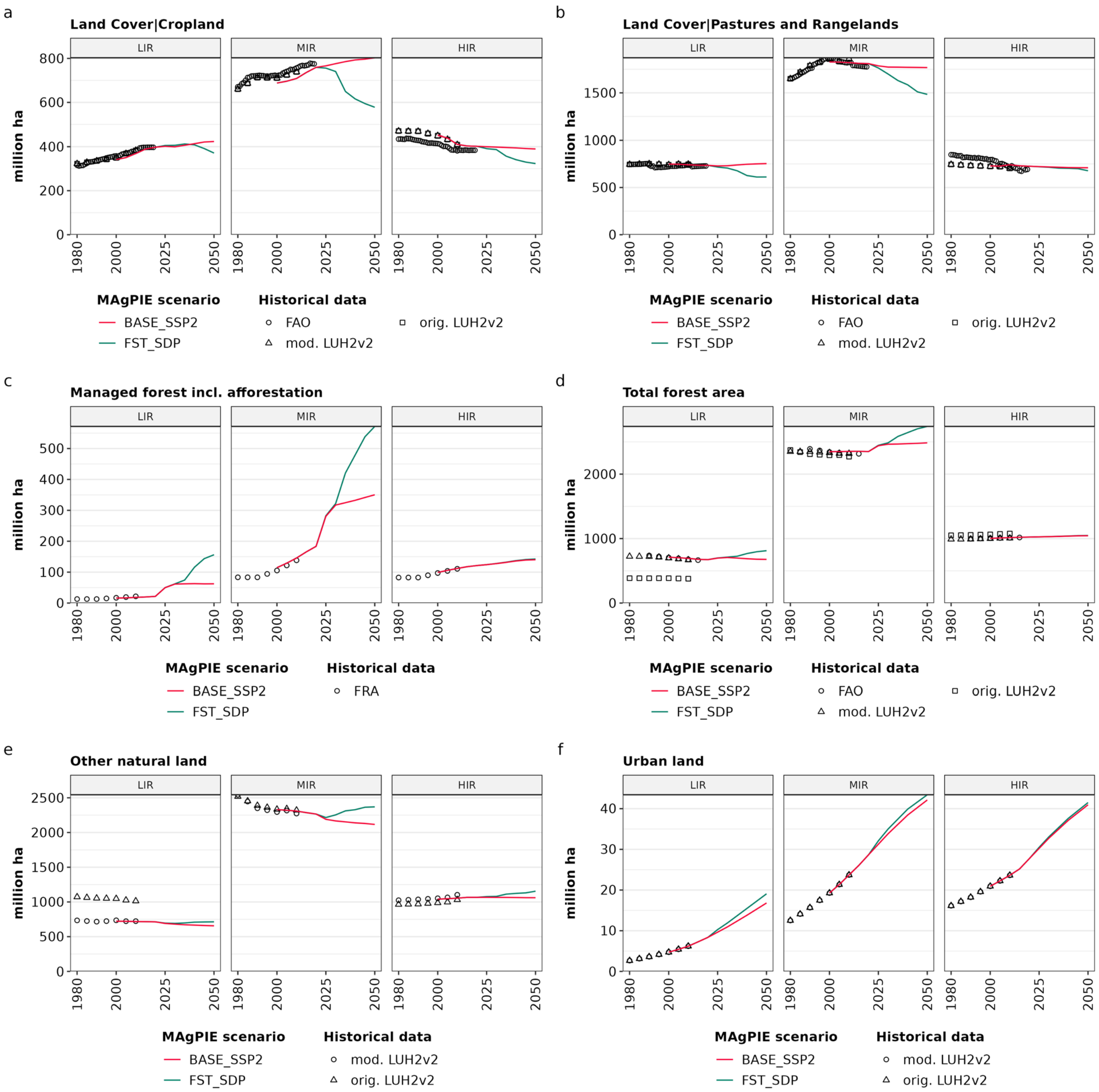

Supplementary Fig. 12

Comparison of land use for cropland (physical area, including fallow) (a), pasture and rangelands (b), managed forests including afforestation (c), total forest area (d), other semi-natural land (e) and urban land (f) with historical trends from LUH2v2[139] for current low-income, middle-income, and high-income regions (see S1.1.1). Modified LUH2v2 was harmonized to match the forest areas from the Global Forest Resource Assessment[246], original LUH2v2 is the original data from LUH2v2[139].

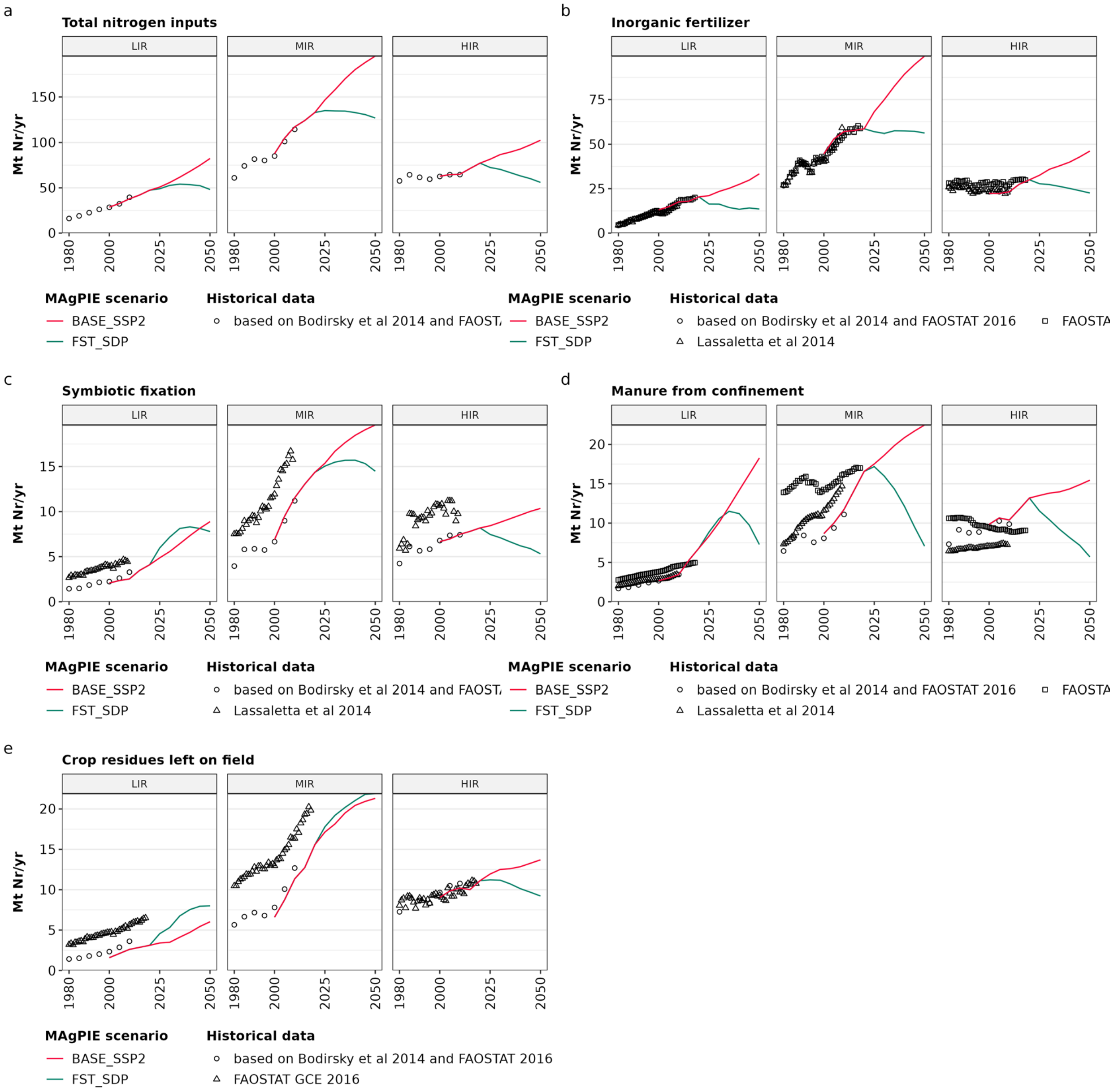

Supplementary Fig. 13

Comparison of nitrogen inputs to croplands, including total nitrogen inputs (a), inorganic fertilizer (b), symbiotic fixation (c), manure from confinement (d), crop reisdues left on field (e) with historical trends from Lassaletta et al[247], FAOSTAT global crop emissions [146], and a dataset that uses the methodology for nitrogen flux estimates of Bodirsky et al.[56] with national historical input data from FAOSTAT[146]. Allocation of inorganic fertilizer between croplands and pastures is based on shares from Lassaletta et al. [247] for all data sources. Comparison is done for current low-income, middle-income, and high-income regions (see S1.1.1).

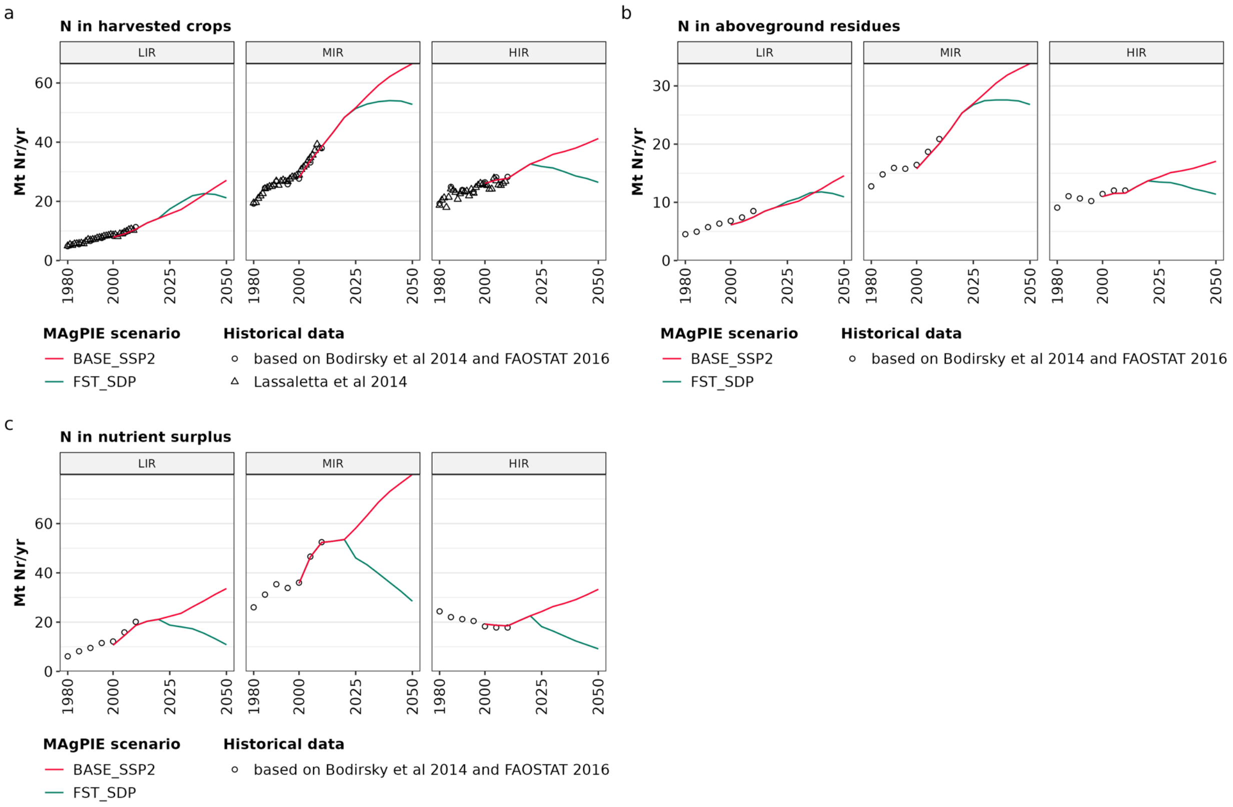

Supplementary Fig. 14

Comparison of nitrogen in harvested crops (a), in aboveground crop residues (b), and nitrogen surpluses on croplands (c). Historical trends from Lassaletta et al. [247] and a dataset that uses the methodology for nitrogen flux estimates of Bodirsky et al. [56] with national historical input data from FAOSTAT[146]. Comparison is done for current low-income, middle-income, and high-income regions (see S1.1.1).

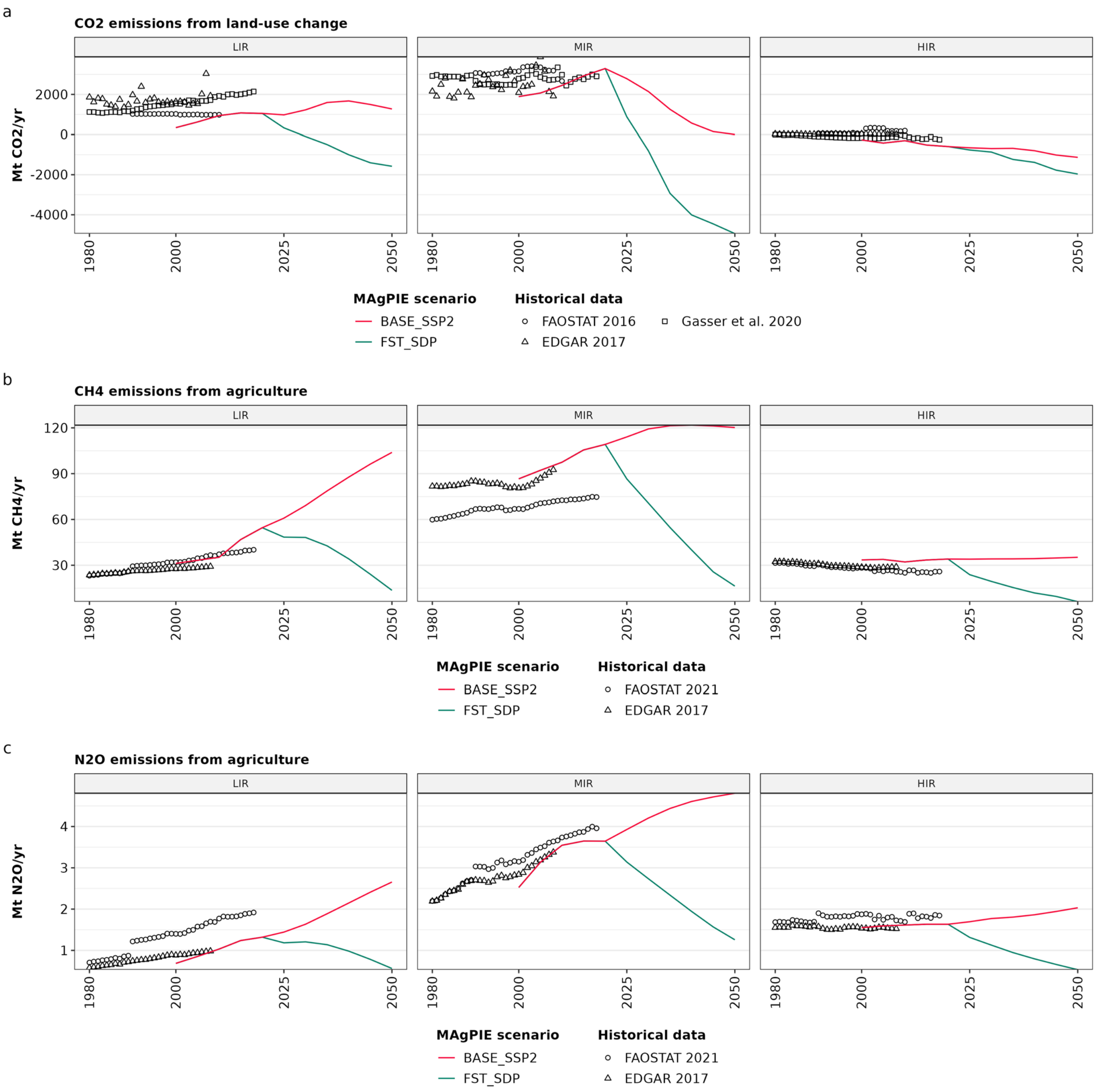

Supplementary Fig. 15

Comparison of the anthropogenic greenhouse gas emissions CO2 (a), CH4 (b) and N2O (c) with historical trends from FAOSTAT Emission Totals[146,240], EDGAR[248] , GASSER et al.[249] (2020) for current low-income, middle-income, and high-income regions (see S1.1.1).

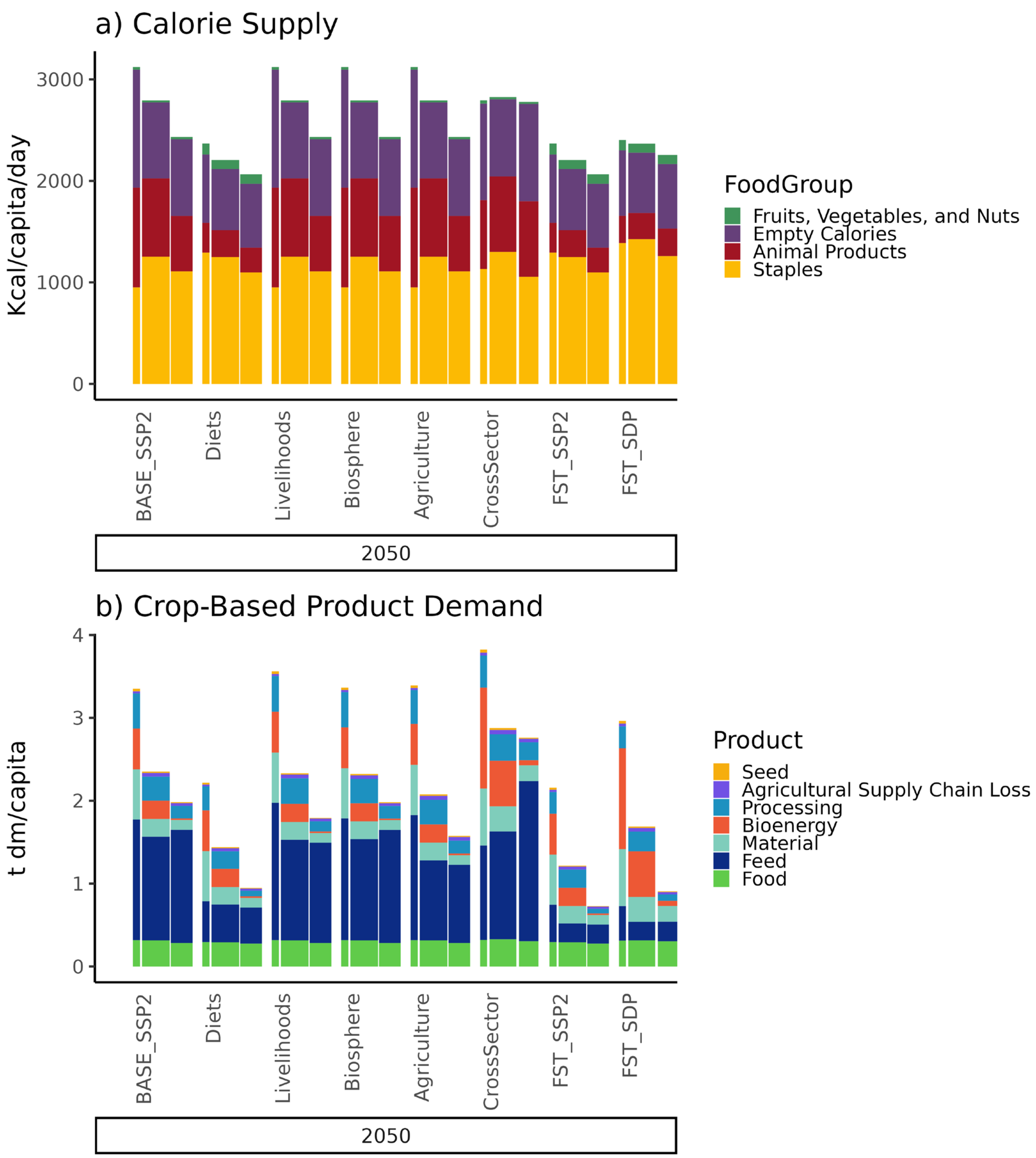

Supplementary Fig. 16

Intermediate results for demand: a) Per-capita food demand by product in kcal per capita per day and b) crop-based product demand by utilization category in t DM per capita per year. Grouped bars include, from left to right, the average values for current high-income, middle-income, and low-income world regions (see S1.1.1). Bar width indicates the population size of these groups. Grouped bars are arranged by scenario and year.

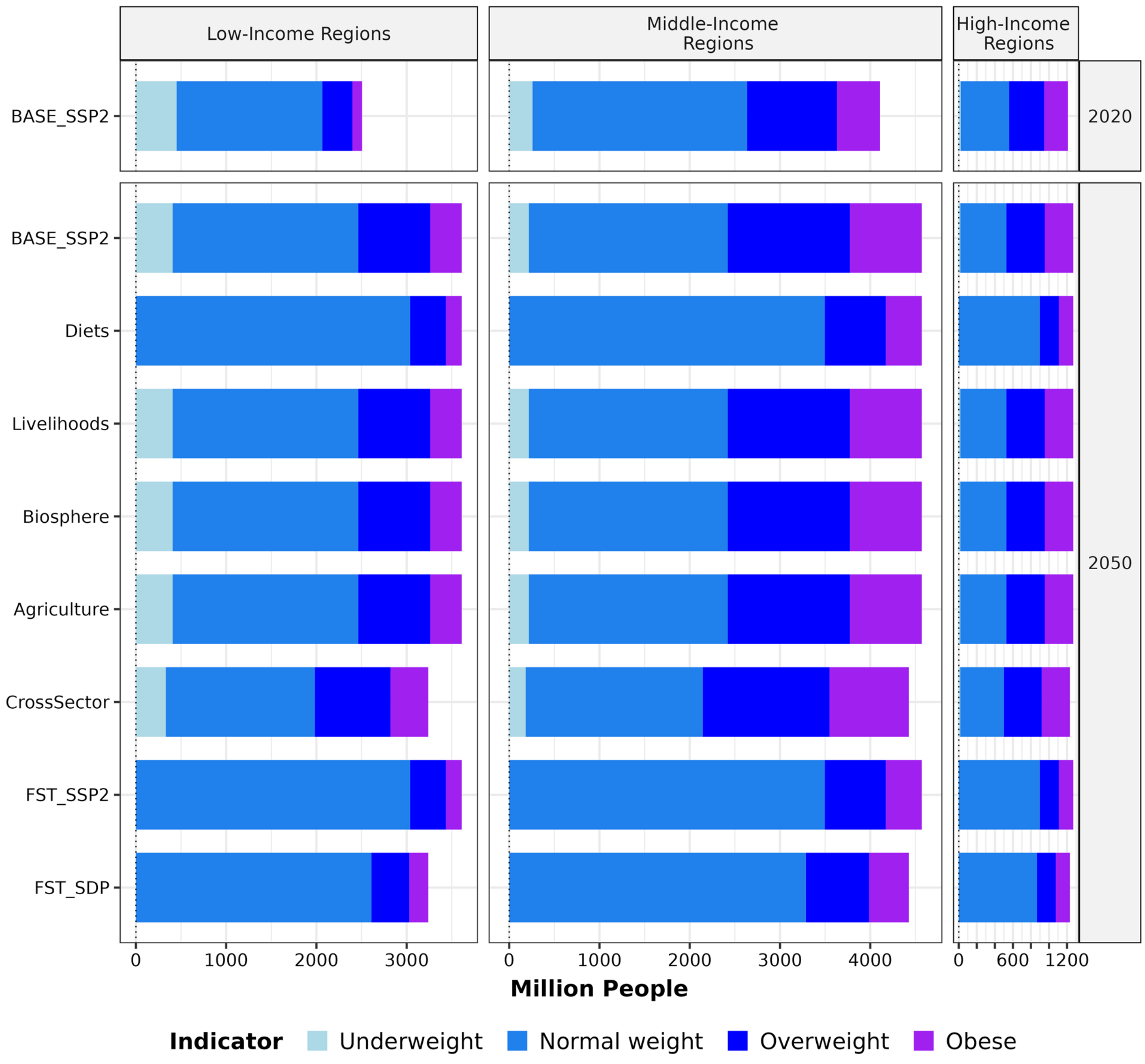

Supplementary Fig. 17

1435 Intermediate results for body-mass-index: Population in million people by weight class for current low-income regions, middle-income, and high-income regions (see S1.1.1).

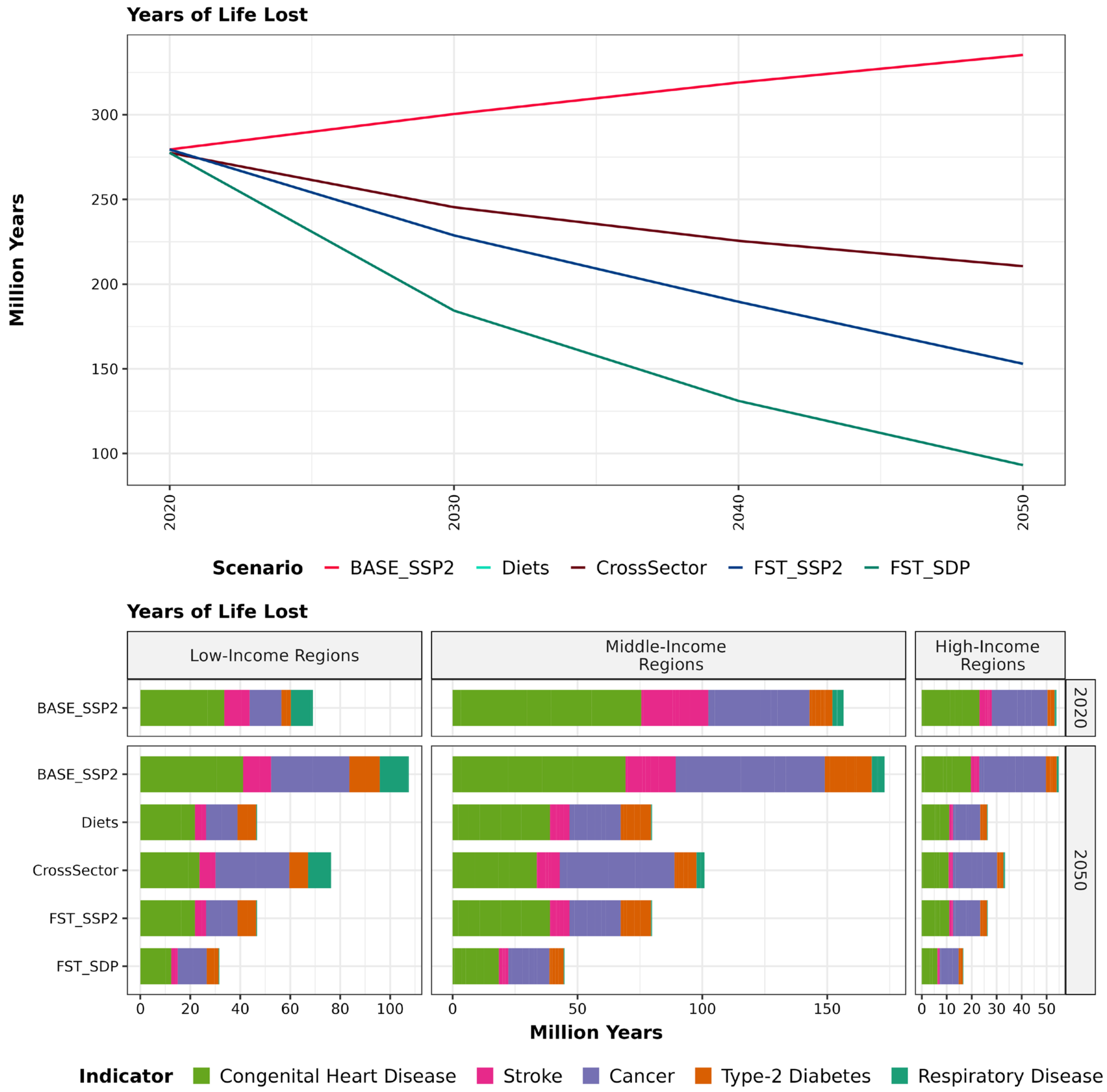

Supplementary Fig. 18

Intermediate results for diet-related diseases. a) Total years of life lost by scenario over time. b) Years of life lost by year and scenario for current low-income regions, middle-income, and high-income regions (see S1.1.1).

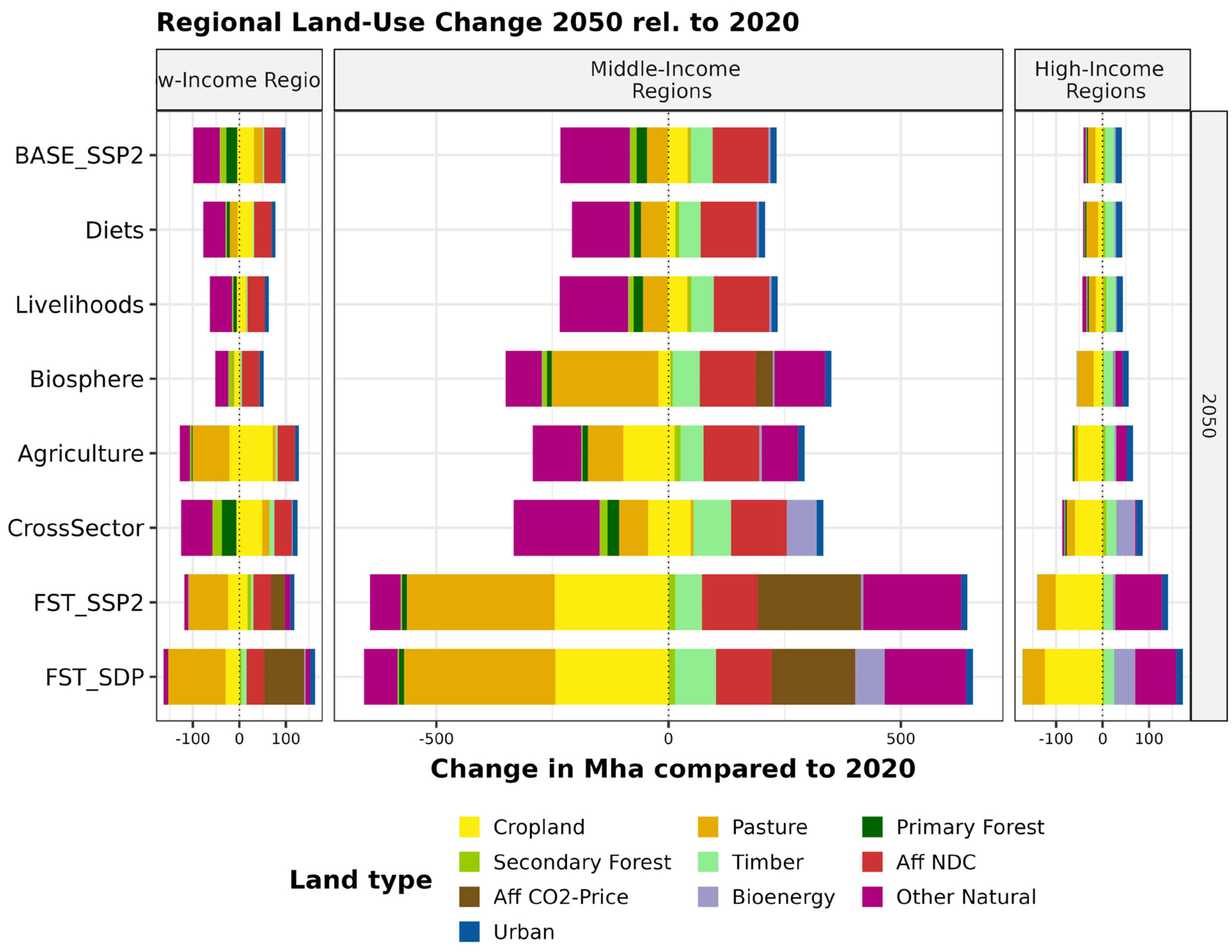

Supplementary Fig. 19

Intermediate results for land use change from 2020 to 2050 in million hectares divided into different land types and scenarios, divided by current low- , middle and high-income regions (see S1.1.1).

a

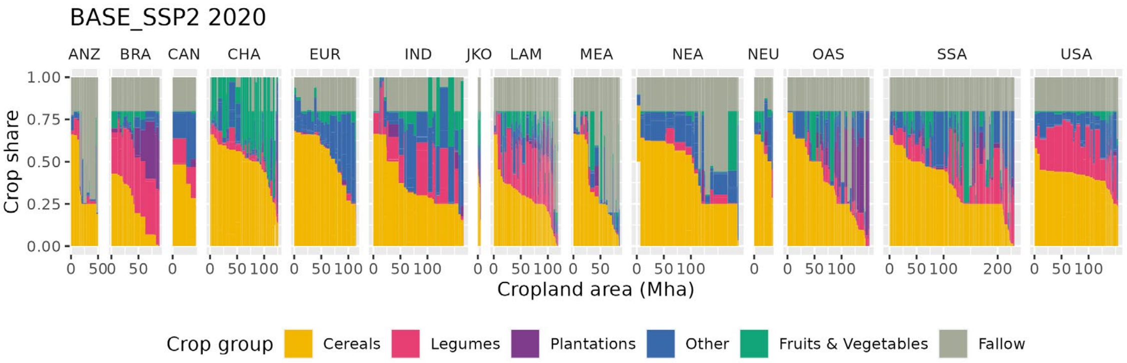

b

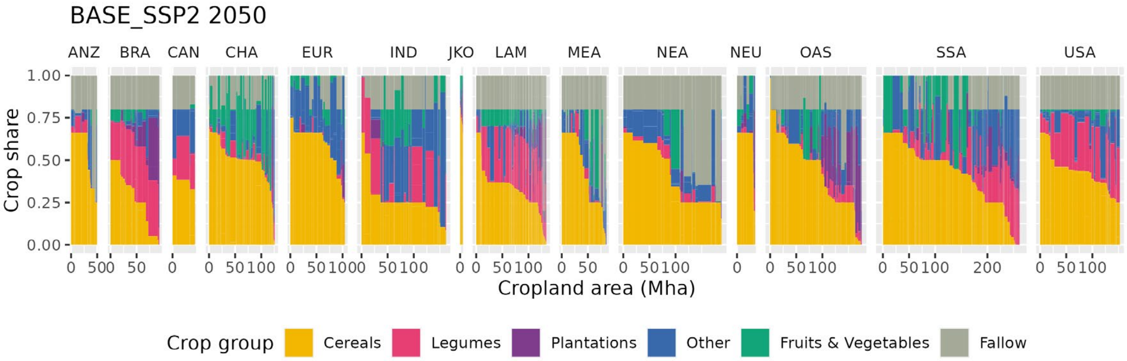

c

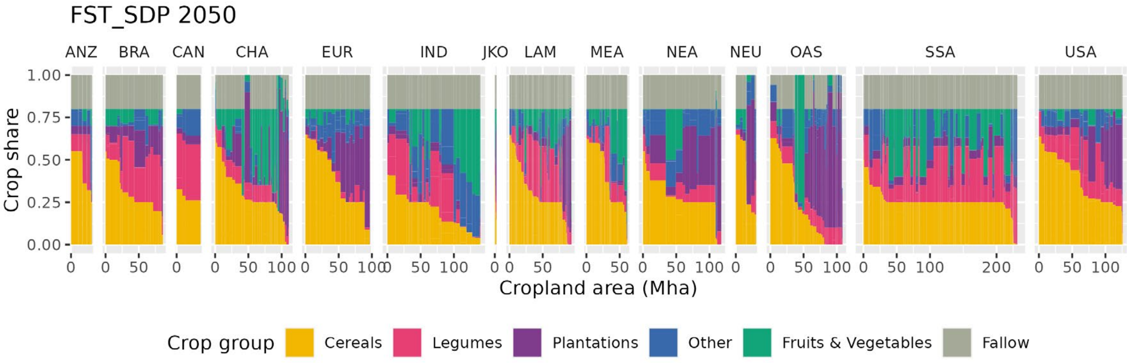

Supplementary Fig. 20

Intermediate results for croparea composition for (a) the scenario $BASE_{SSP2}$ in the year 2020, (b) in the year 2050, and (c) for the FST_{SDP} pathway in the year 2050. Y-axis shows the shares of major crop groups within the crop area of a cluster cell, and x-Axis shows the size of a cluster cell within major world regions (see section S1.1.1). Plantations include grassy and woody cellulosic bioenergy plants, oilpalms and sugar cane. Other crops include for example roots and forage crops.

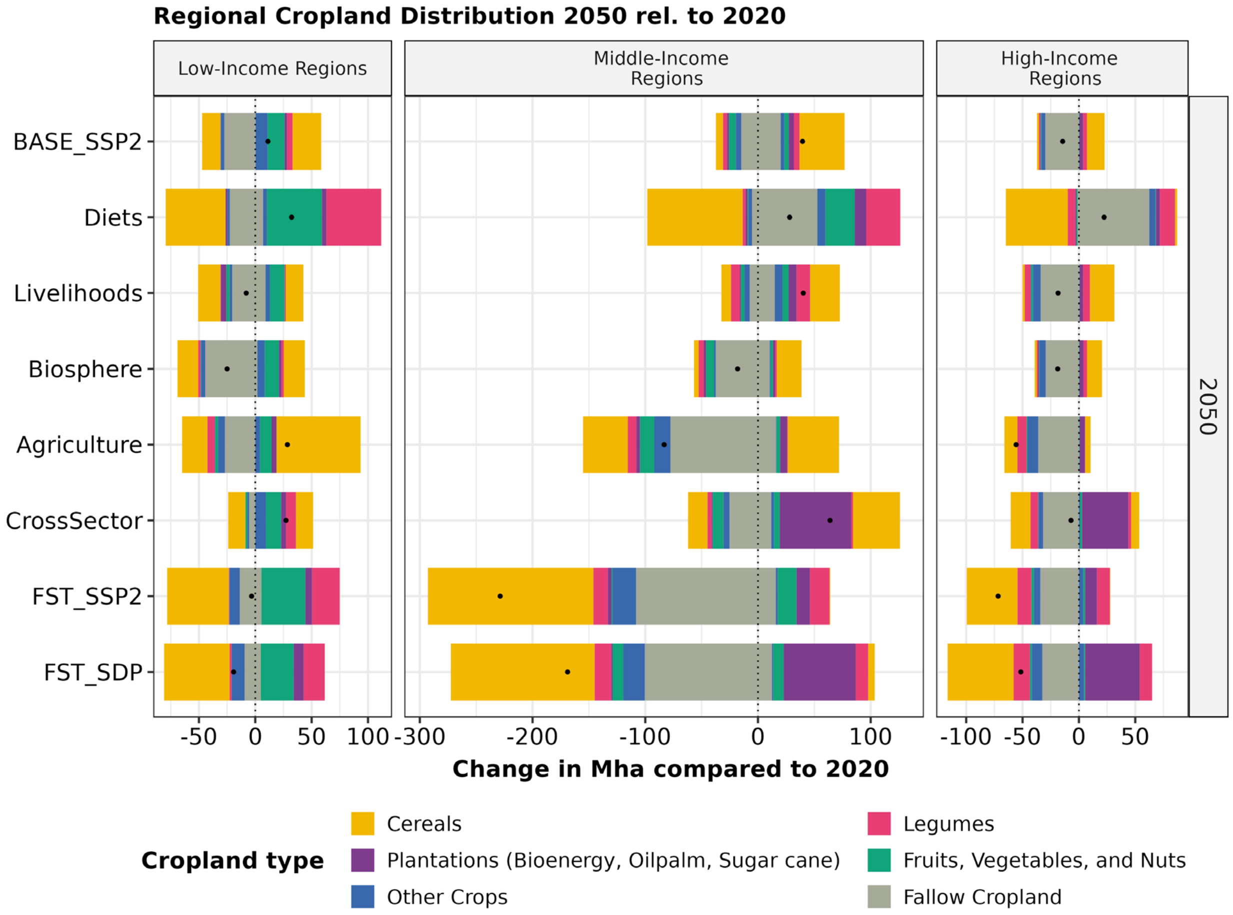

1465

Supplementary Fig. 21

Intermediate results for croparea change for major crop groups.

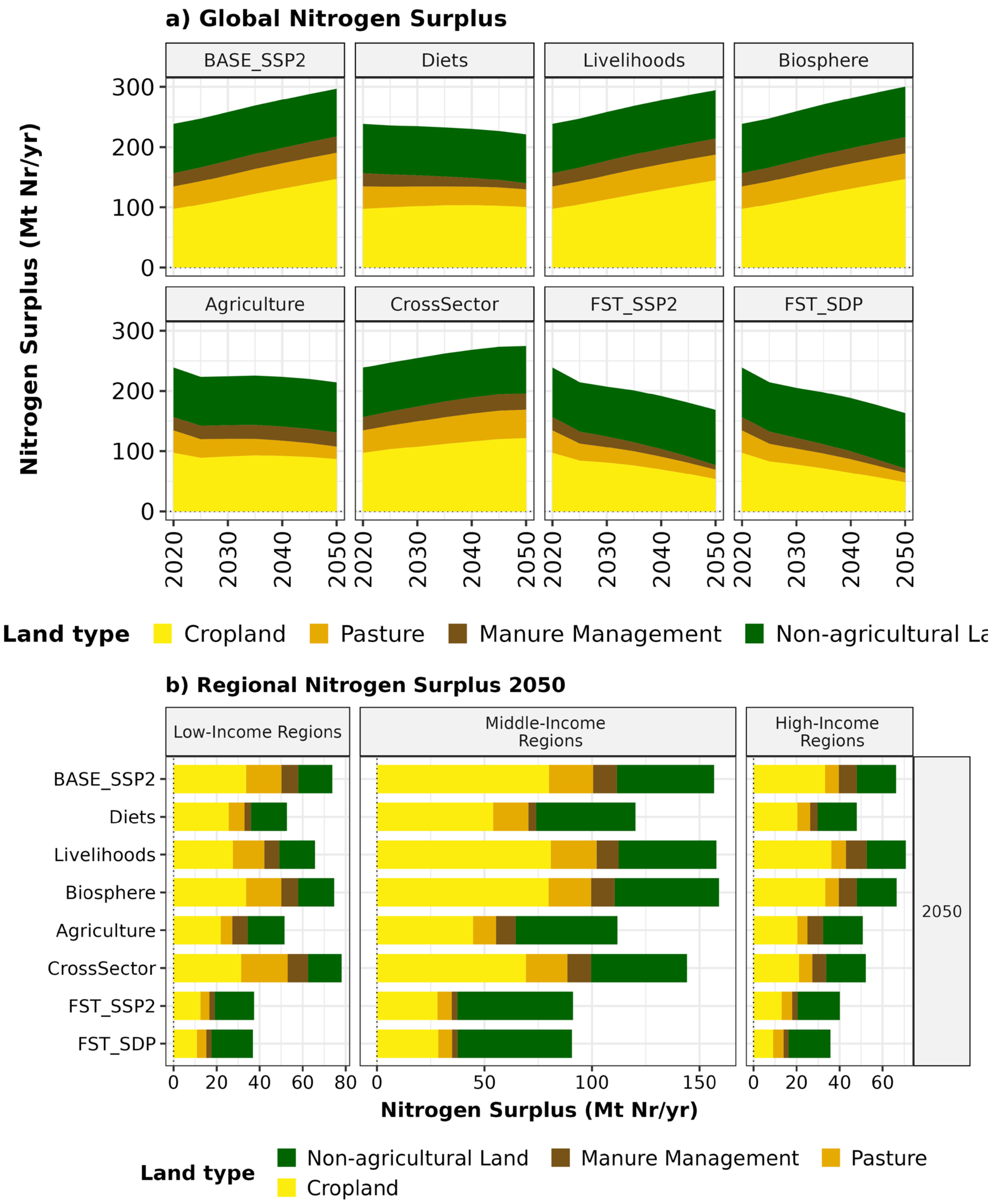

Supplementary Fig. 22

Intermediate results for nitrogen surpluses in million tons of reactive nitrogen (Nr) per year by source for various scenarios. (a) Global over time; (b) For current low-, middle- and high-income regions (see S1.1.1) in the year 2050.

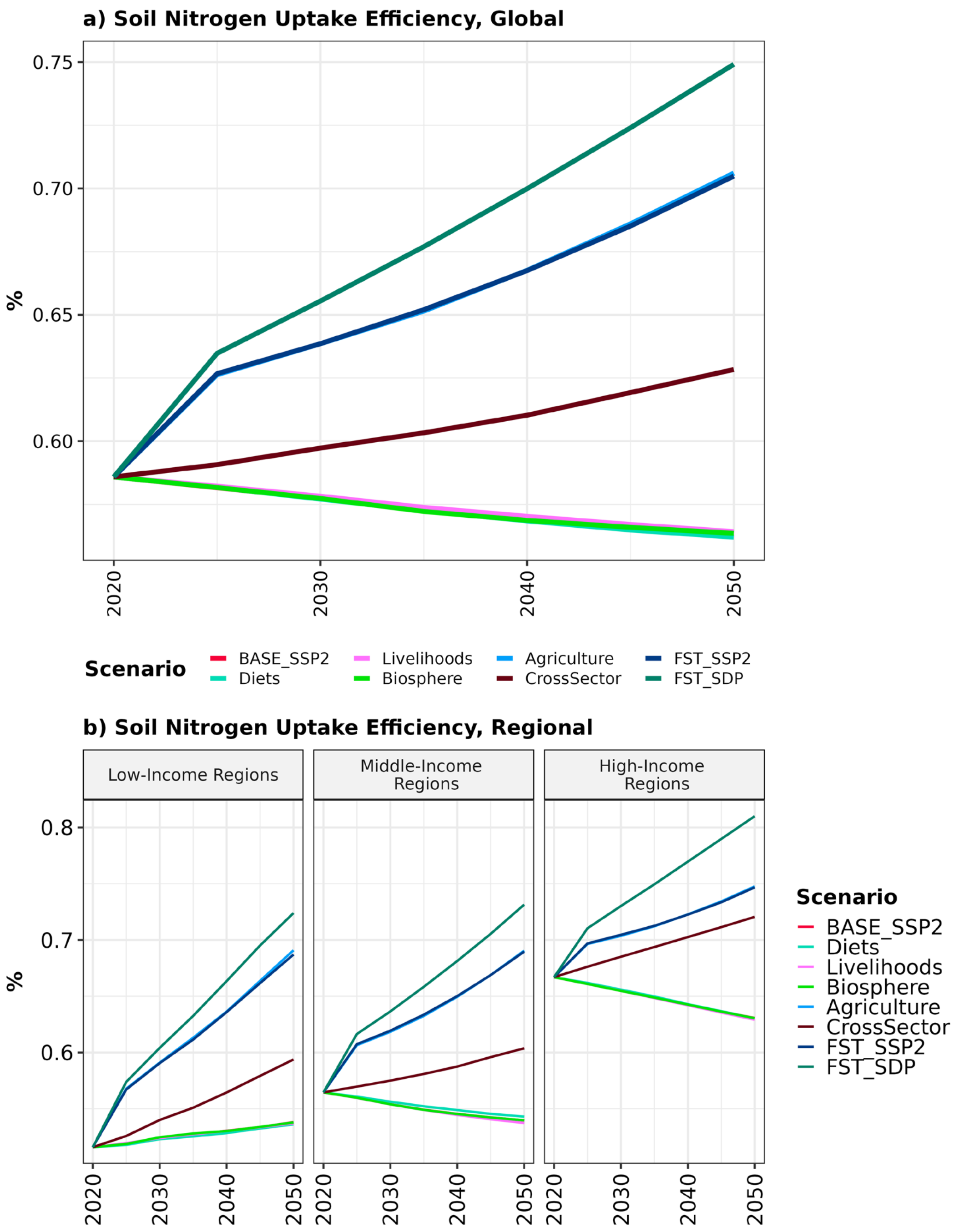

Supplementary Fig. 23

Intermediate results for soil nitrogen uptake efficiency (SNUpE) globally over time (a), and differentiatedd by low-, middle- and high-income regions (b) for different scenarios.

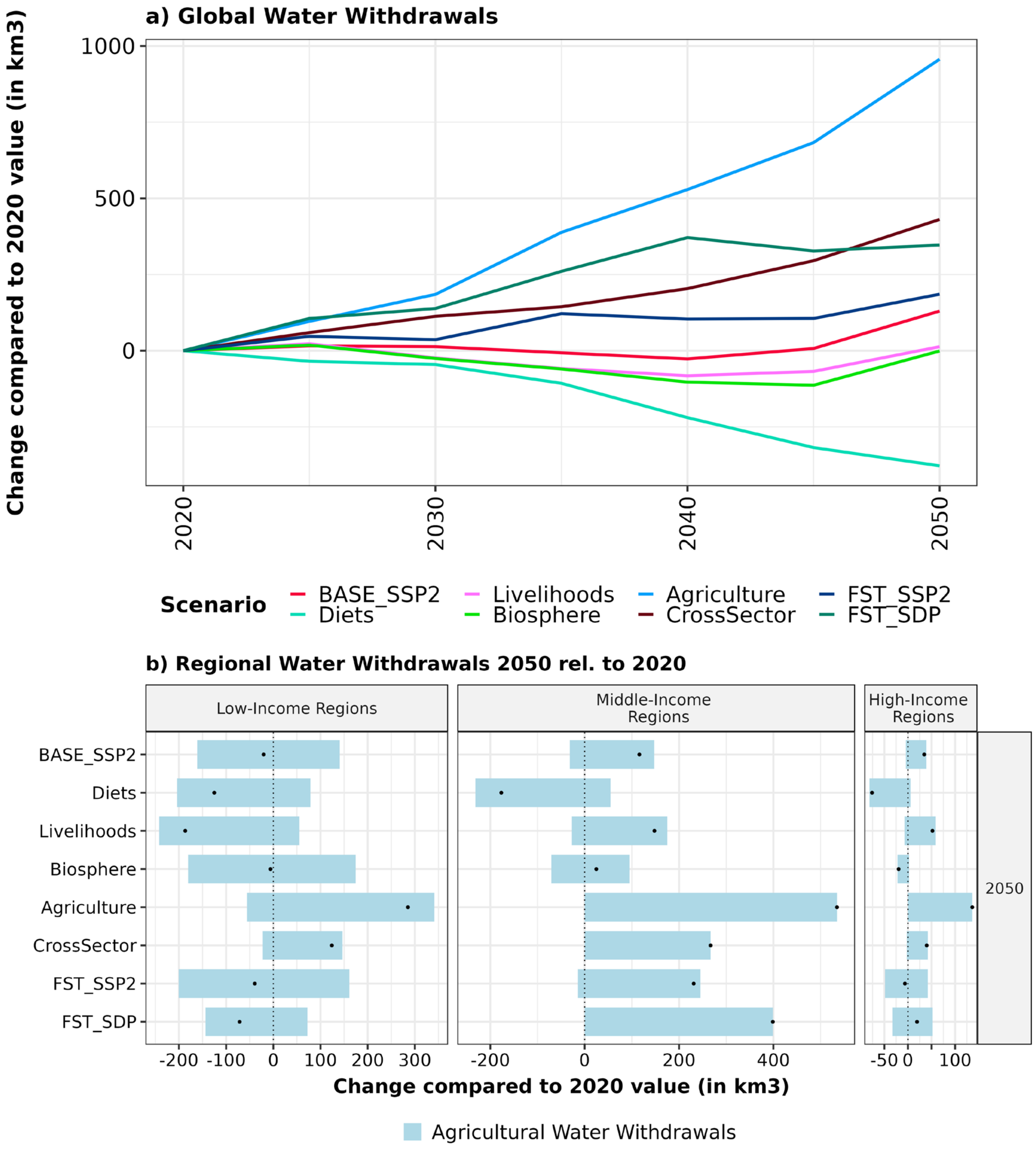

Supplementary Fig. 24

Intermediate results for agricultural water withdrawals in km³ per year for various scenarios over time (a) and in 2020 and 2050 for current low-income, middle-income, and high-income regions (see S1.1.1).

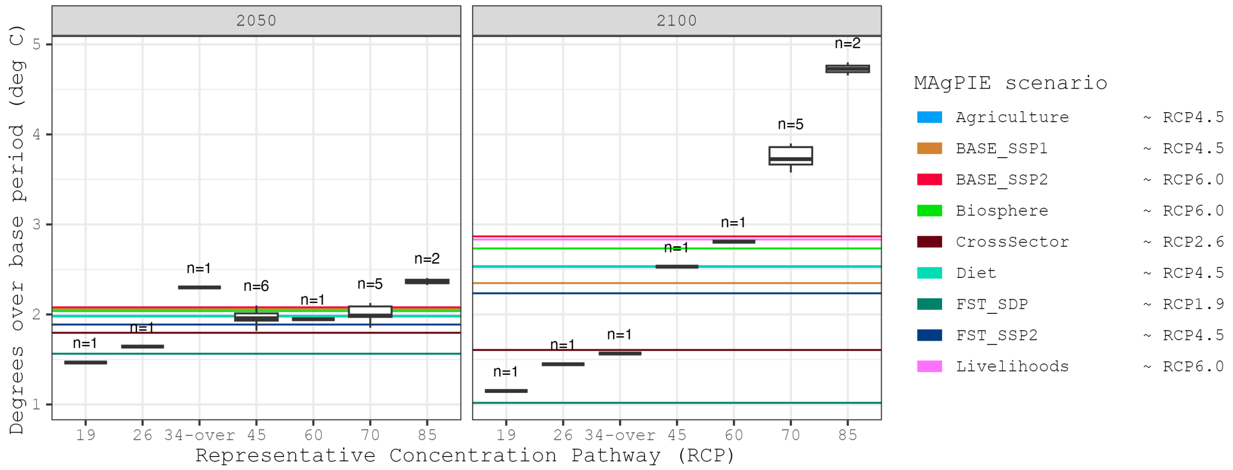

Supplementary Fig. 25

Intermediate results for Global Surface Temperature warming relative to reference period (1850-1900). Boxplots are estimates by the MRI-ESM2 climate model for the Representative Concentration Pathways (RCPs). Box bounds represent the 25th and 75th percentiles (first and third quartiles), with the centre line showing the median (50th percentile). Whiskers extend to the most extreme data points within 1.5 times the interquartile range (IQR) from the box bounds. Some scenarios have only a single climate model run, others have an ensemble of n runs based on multiple random seeds for the model. Colored lines are estimates by the MAGICC climate emulator for the greenhouse gas emission trajectories of the scenarios simulated for this analysis. In the legend, our scenarios are mapped to the respective RCP with the lowest sum of temperature divergences in 2050 and 2100.

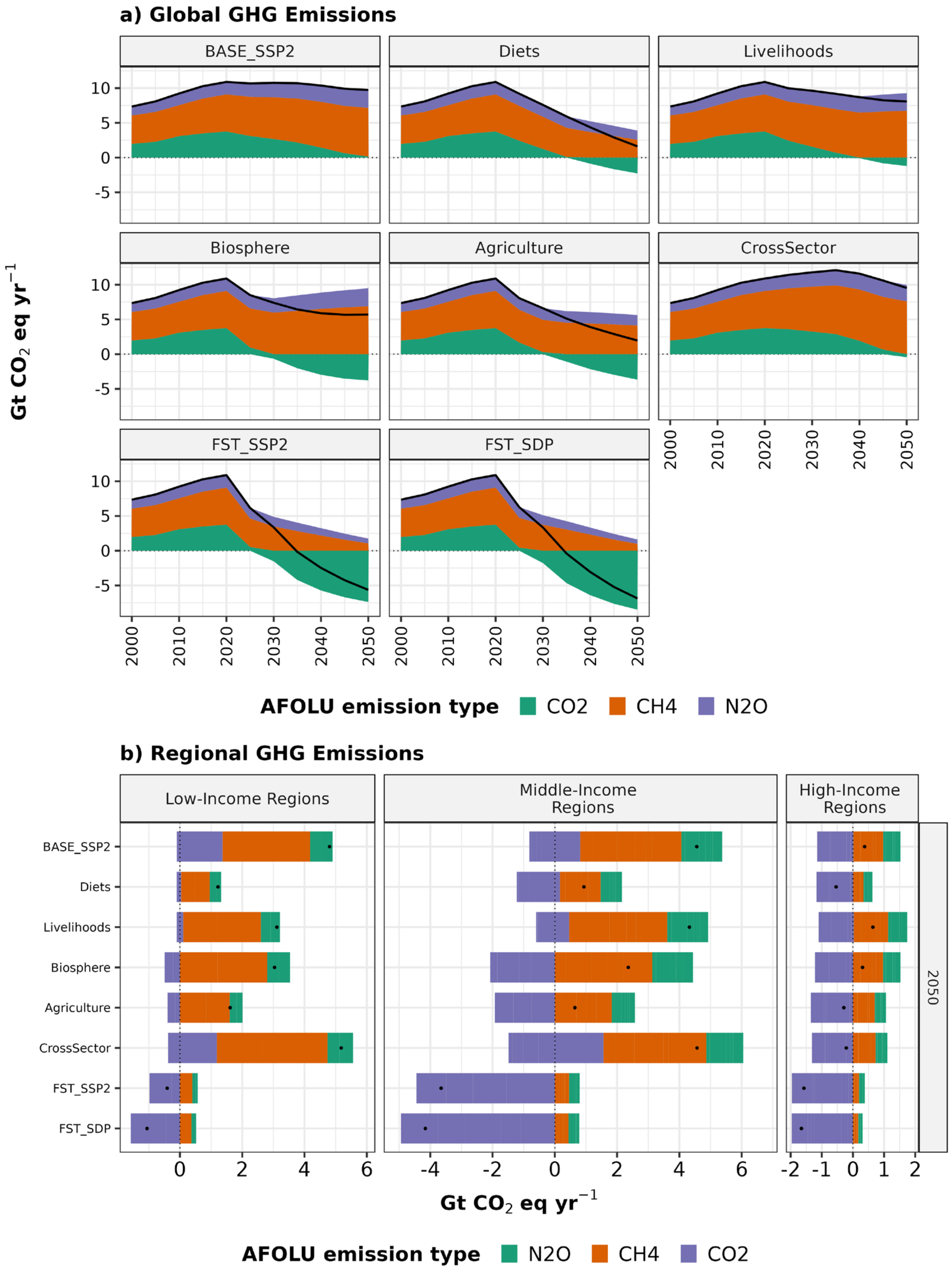

Supplementary Fig. 26

Intermediate results for greenhouse gas emissions of N_2O, CH_4 and CO_2 in CO_2-equivalents over time (a) and cumulated for the period 2020-2050 for current low-, middle-and high-income world regions (see S1.1.1) (b). Dots indicate net value.

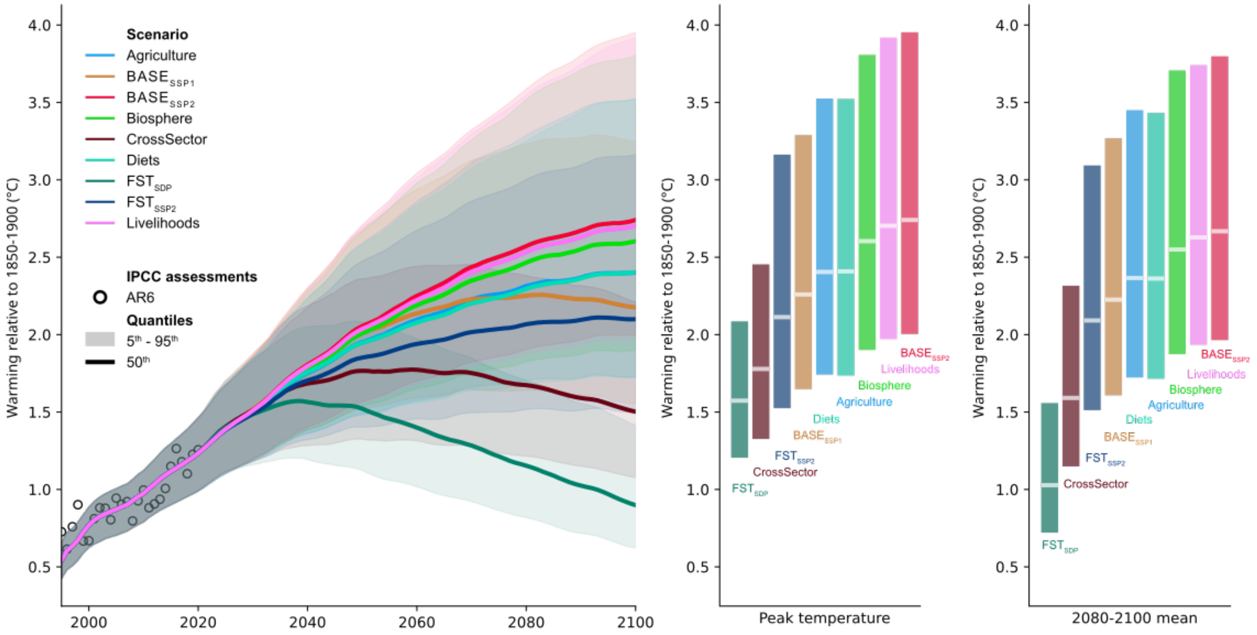

Supplementary Fig. 27

Intermediate results for global warming relative to 1850-1900 from MAGICC7.5.3 for key scenarios until 2100 (left panel); peak temperature during this period (middle panel); mean temperature in the later fifth of the 21st century (right panel). Lines represent median value across 600 runs of the MAGICC reduced-complexity climate model. Ribbons represent 5th-95th percentiles.

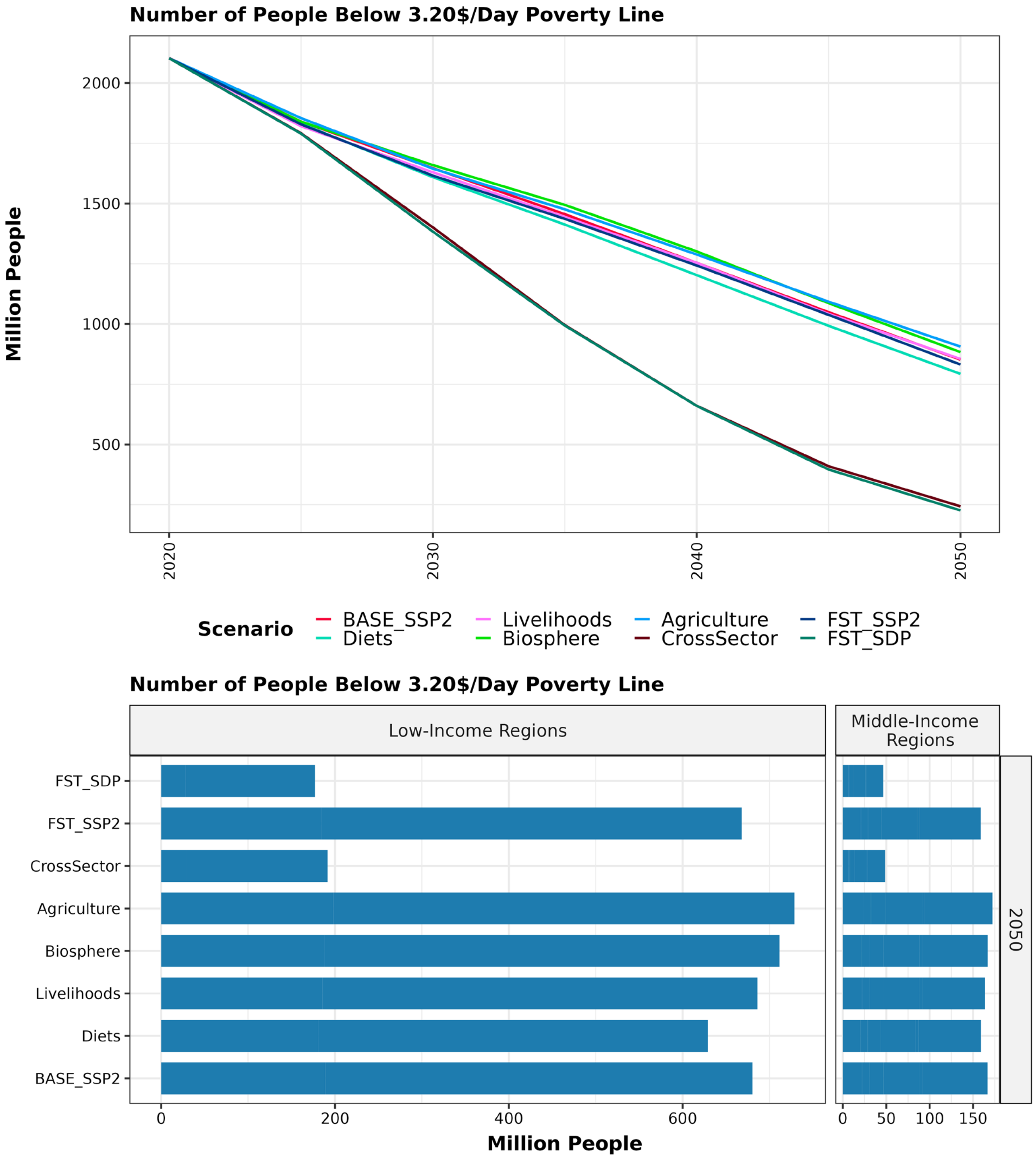

Supplementary Fig. 28

Intermediate results for the number of people living with less than 3.20 $USD_{2011PPP}$ as global total over time (a) and for current low- and middle-income regions (see S1.1.1) in the year 2050.

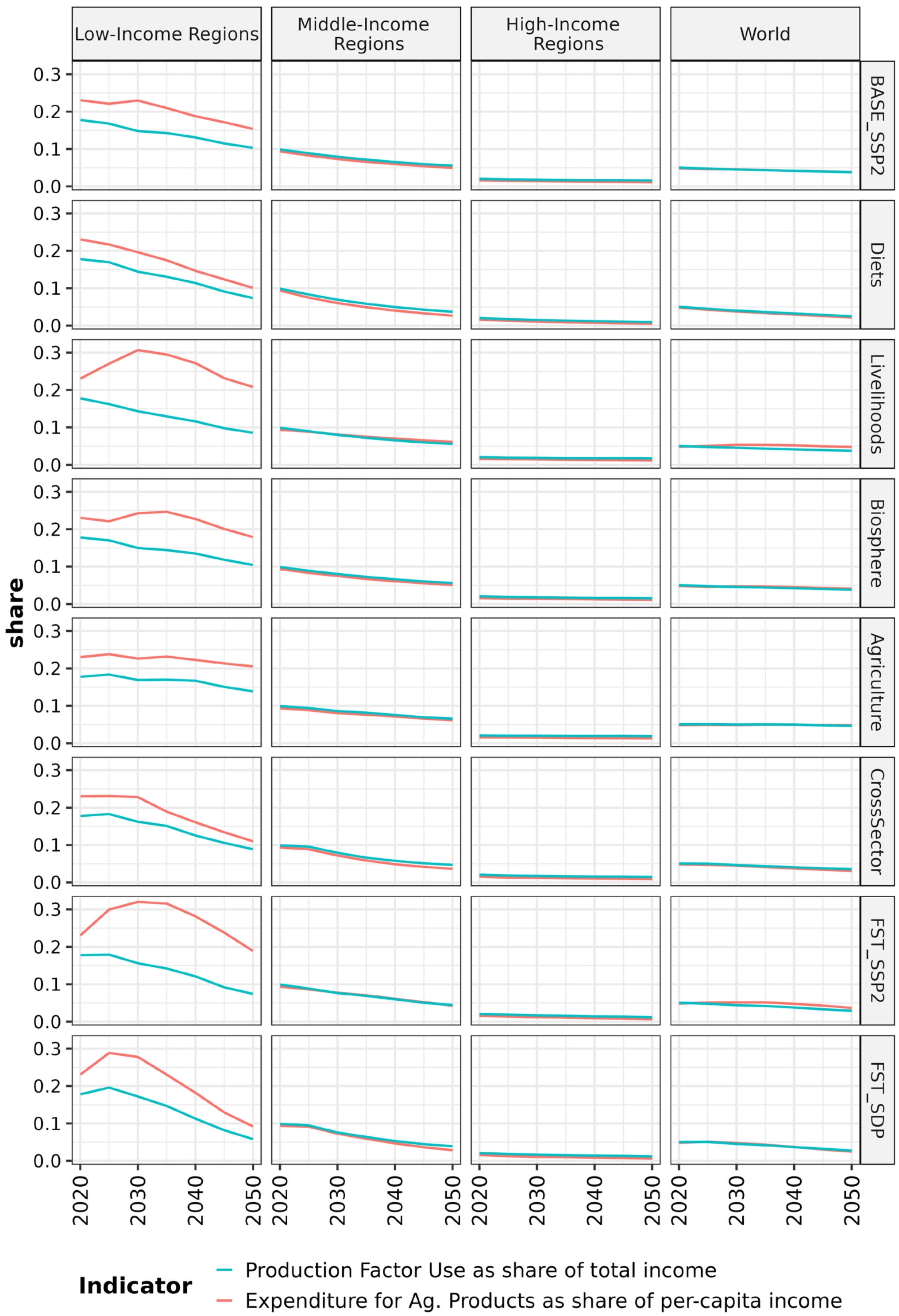

Supplementary Fig. 29

Intermediate results for the Production-Factor Use as share of total income and the Expenditure for Agricultural Products as share of per-capita income in current low-, middle- and high-income world regions (see S1.1.1).

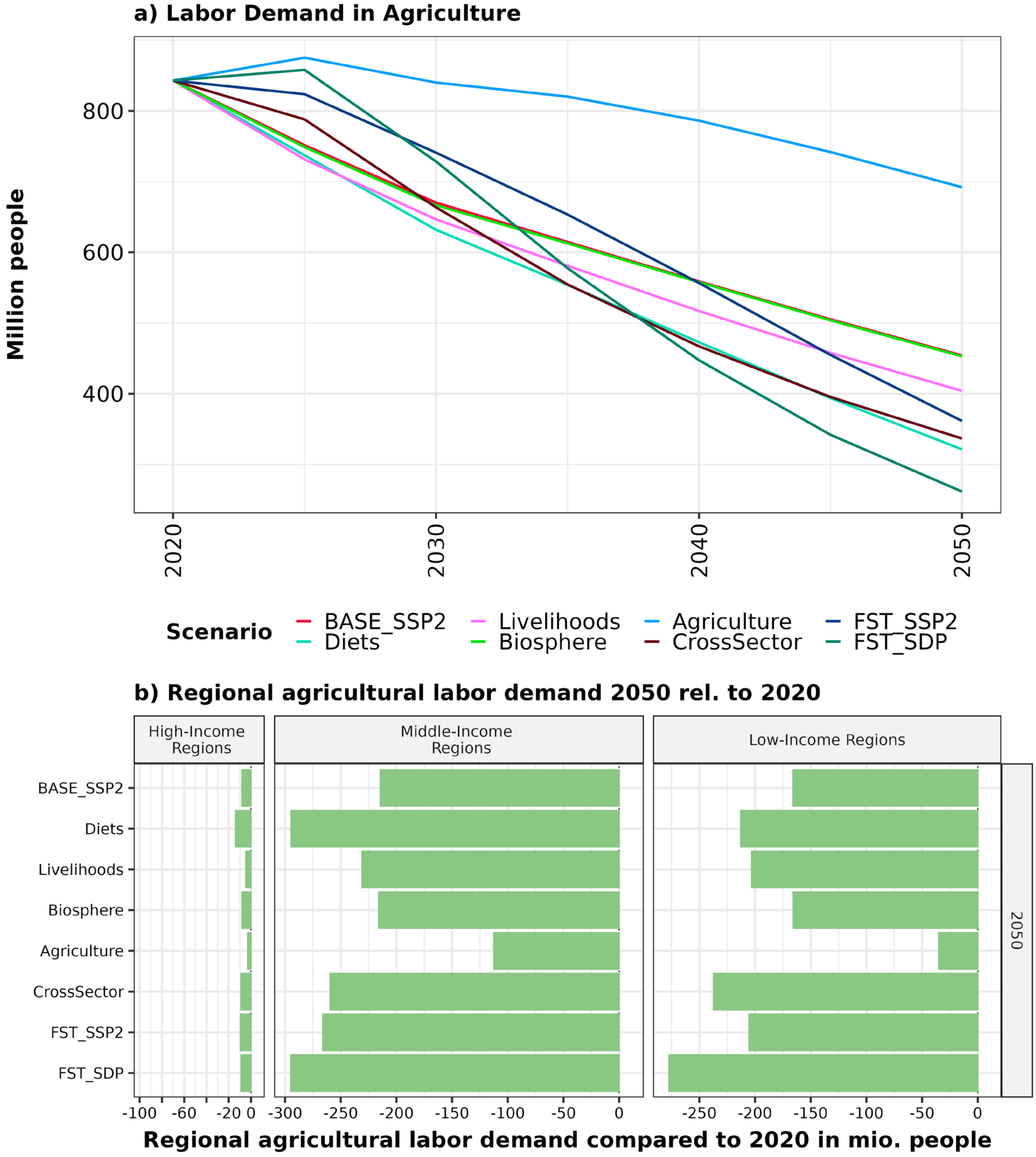

1540 Supplementary Fig. 30

Intermediate results for *Agricultural Labor Demand* in million people for various scenarios, (a) over time and (b) in 2050 as change relative to 2020 in current low-, middle- and high-income world regions (see S1.1.1).

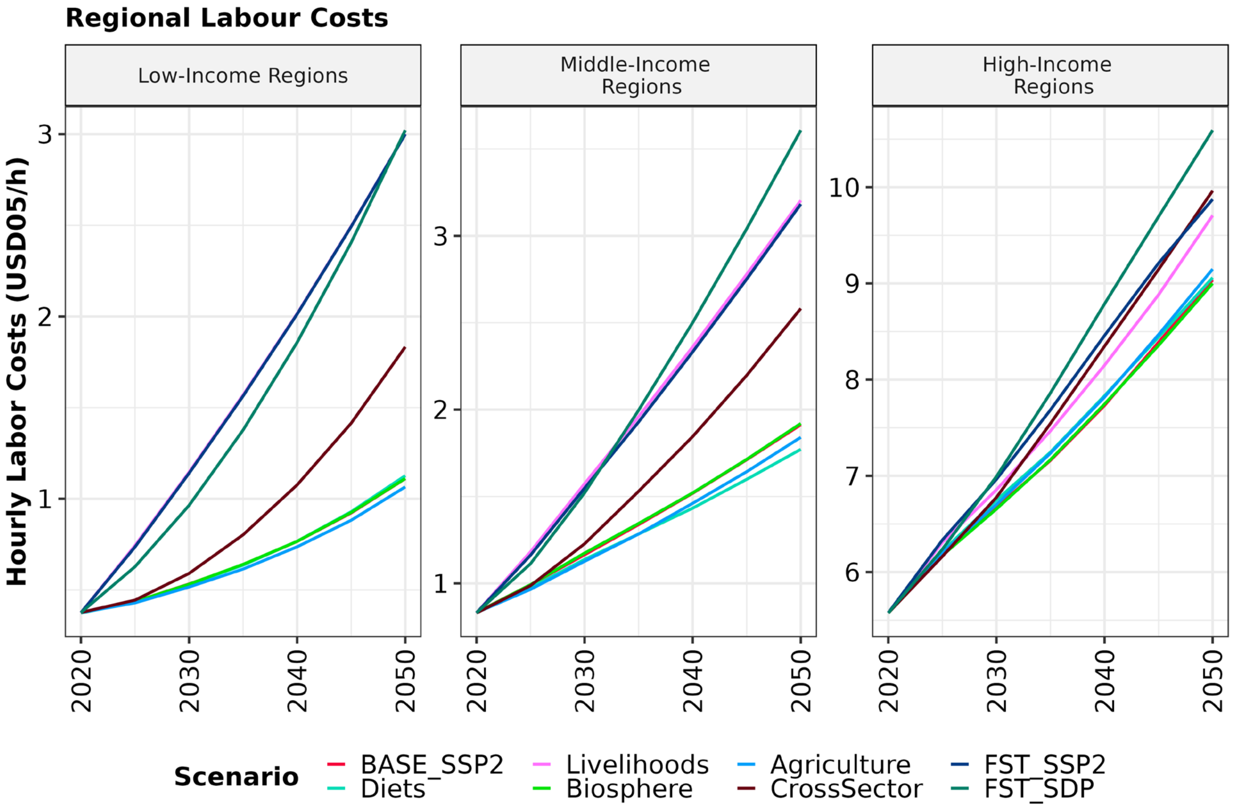

Supplementary Fig. 31

Intermediate results for hourly labor costs in $USD_{2005MER}$ in current low-, middle- and high-income regions (see S1.1.1) over time for various scenarios.

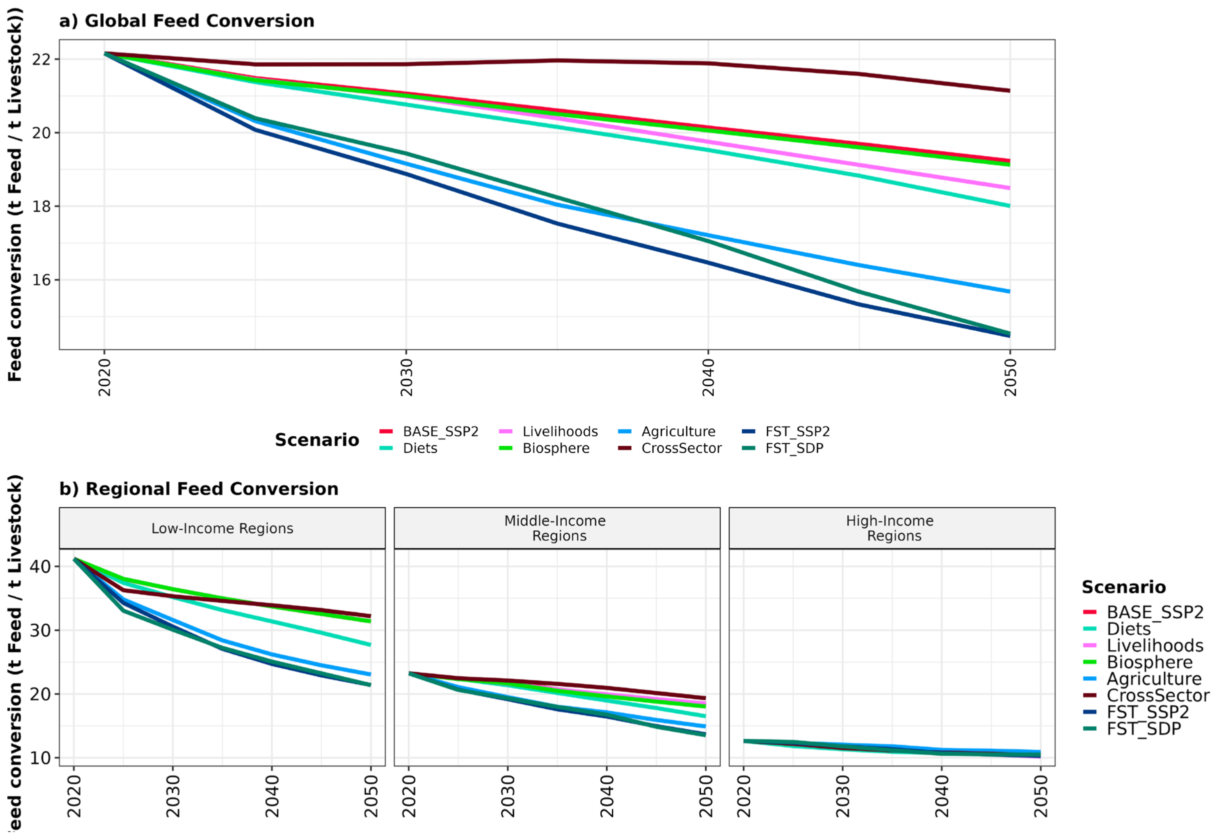

Supplementary Fig. 32

Intermediate results for the development of feed conversion efficiency in tons of feed per tons of livestock over time as global average (a) and in current low-, middle- and high-income regions (see S1.1.1) over time for various scenarios.

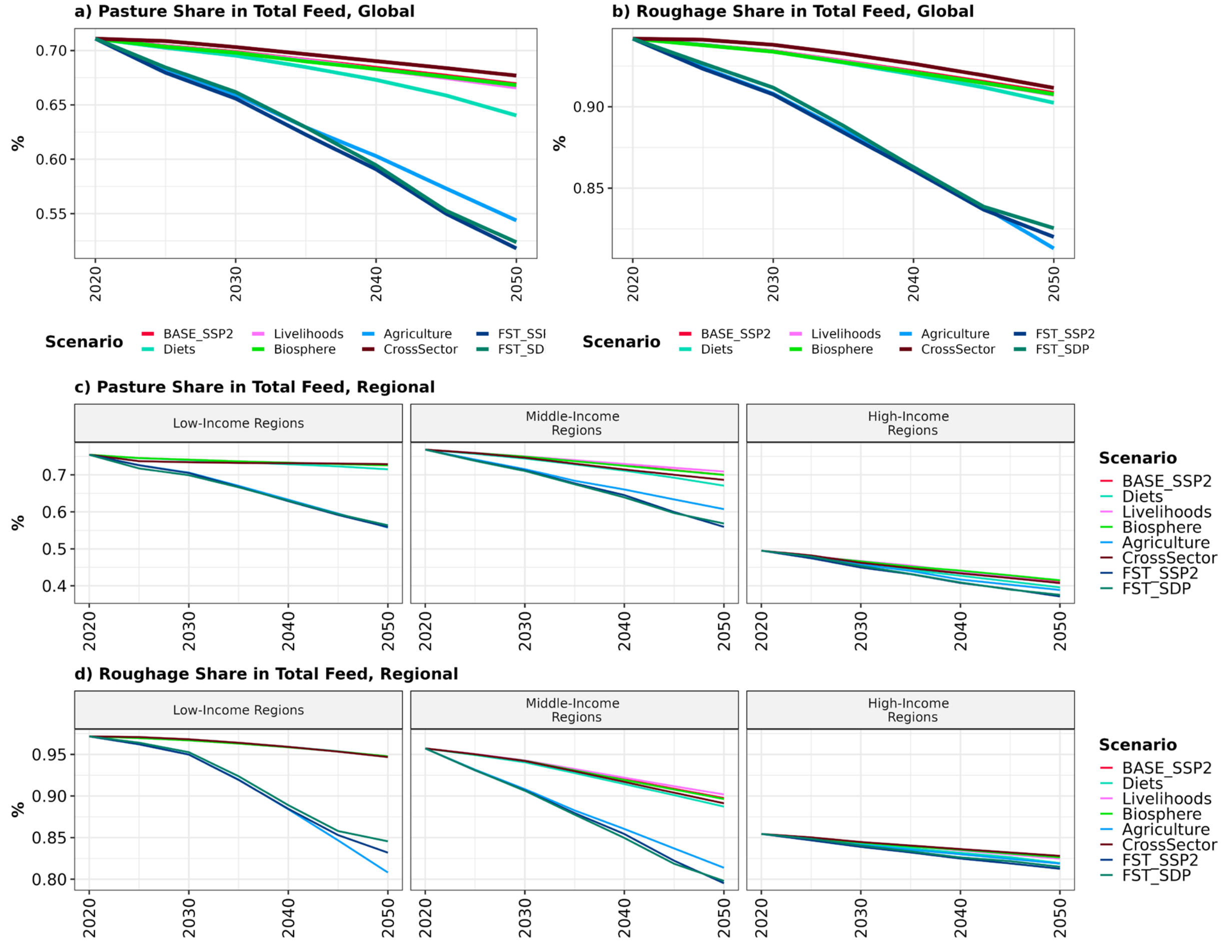

Supplementary Fig. 33

Intermediate results for the share of pasture (a, c) and roughage - including pasture, forage crops and crop residues (b, d) within total feed ratios on a dry matter basis. Globally (a, b) and separated by current low, medium and high-income regions (c, d)

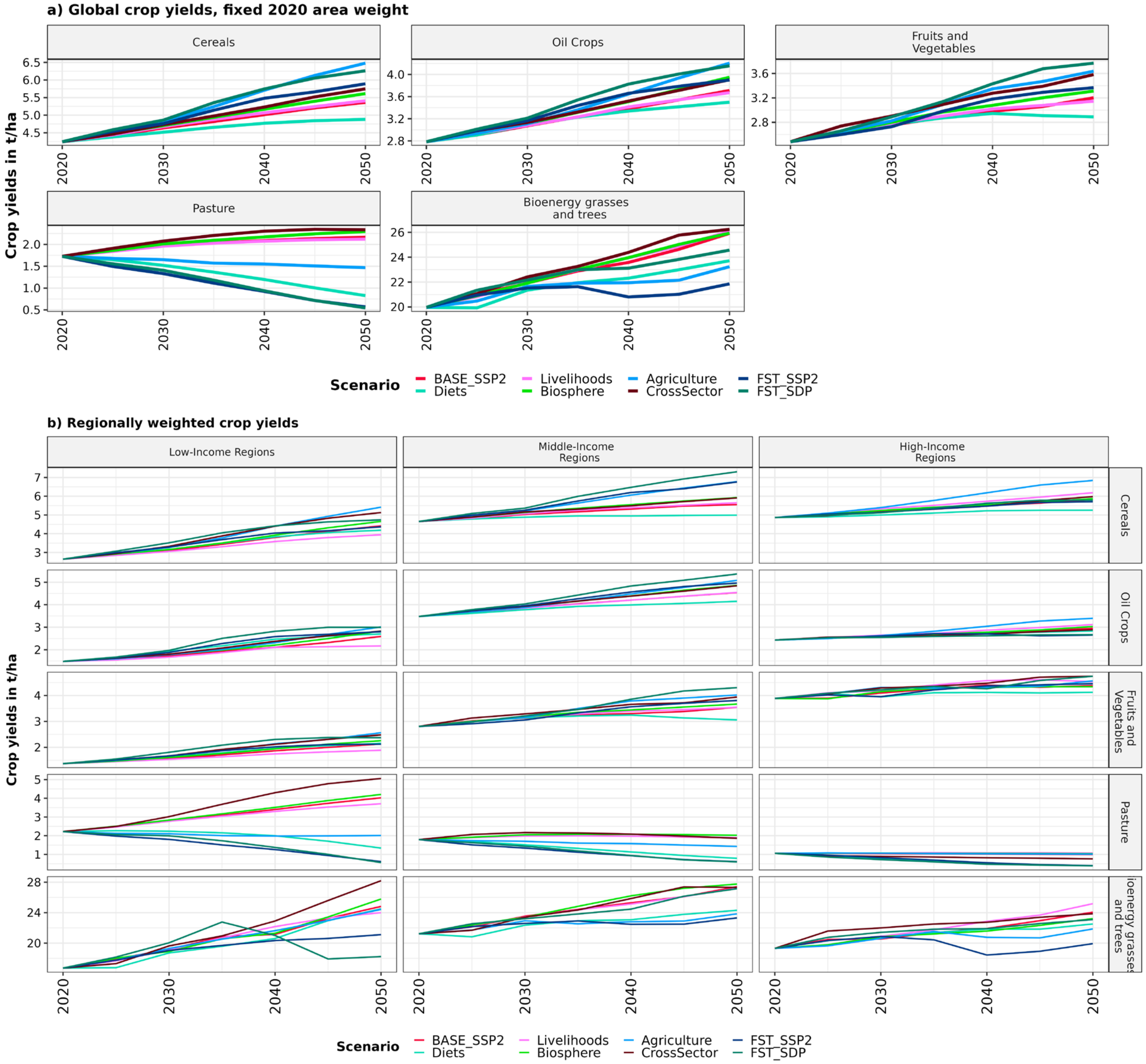

Supplementary Fig. 34

Intermediate results for crop yields in t DM per hectare by crop type, globally (a) and by current low-, middle-, and high-income regions (b).

Milestone	Indicator	2020	2030	2040	2050	Mapping to SDG/GBF targets and indicators
Total per-capita food supply in current LIRs	kcal/capita/day	2592	2831	2960	2980	
Total per-capita food supply in current MIRs	kcal/capita/day	3020	3079	3048	3000	
Total per-capita food supply in current HIRs	kcal/capita/day	3441	3183	3110	3042	
Total per-capita food intake in current LIRs	kcal/capita/day	2167	2309	2412	2486	
Total per-capita food intake in current MIRs	kcal/capita/day	2357	2445	2493	2513	
Total per-capita food intake in current HIRs	kcal/capita/day	2555	2573	2576	2574	
Per-capita intake of cereals in current LIRs	kcal/capita/day	1060	924	848	886	
Per-capita intake of cereals in current MIRs	kcal/capita/day	965	906	935	993	
Per-capita intake of cereals in current HIRs	kcal/capita/day	635	795	892	972	
Per-capita intake of legumes in current LIRs	kcal/capita/day	121	191	263	345	
Per-capita intake of legumes in current MIRs	kcal/capita/day	69	156	246	335	
Per-capita intake of legumes in current HIRs	kcal/capita/day	60	161	252	341	
Per-capita intake of fruits, vegetables and nuts in current LIRs	kcal/capita/day	96	147	196	236	
Per-capita intake of fruits, vegetables and nuts in current MIRs	kcal/capita/day	171	203	244	269	
Per-capita intake of fruits, vegetables and nuts in current HIRs	kcal/capita/day	162	210	229	245	
Per-capita intake of alcoholic beverages in current LIRs	kcal/capita/day	26	28	31	25	
Per-capita intake of alcoholic beverages in current MIRs	kcal/capita/day	65	57	39	24	
Per-capita intake of alcoholic beverages in current HIRs	kcal/capita/day	126	93	63	35	
Per-capita intake of sugar in current LIRs	kcal/capita/day	181	180	160	104	
Per-capita intake of sugar in current MIRs	kcal/capita/day	192	186	150	96	
Per-capita intake of sugar in current HIRs	kcal/capita/day	338	257	184	112	
Per-capita intake of pig meat in current LIRs	kcal/capita/day	10	12	13	7	
Per-capita intake of pig meat in current MIRs	kcal/capita/day	156	120	66	18	
Per-capita intake of pig meat in current HIRs	kcal/capita/day	124	77	44	14	
Per-capita intake of ruminant meat in current LIRs	kcal/capita/day	27	28	29	15	
Per-capita intake of ruminant meat in current MIRs	kcal/capita/day	85	67	41	15	
Per-capita intake of ruminant meat in current HIRs	kcal/capita/day	121	69	36	11	
Per-capita intake of dairy products in current LIRs	kcal/capita/day	157	177	153	96	
Per-capita intake of dairy products in current MIRs	kcal/capita/day	118	126	112	86	
Per-capita intake of dairy products in current HIRs	kcal/capita/day	281	209	165	125	
Per-capita intake of poultry and eggs in current LIRs	kcal/capita/day	33	52	58	40	

Per-capita intake of poultry and eggs in current MIRs	kcal/capita/day	103	104	82	54	
Per-capita intake of poultry and eggs in current HIRs	kcal/capita/day	136	102	81	57	
Production of livestock products, global	Mt DM	314	301	241	148	
Production of fruits, vegetables and nuts, global	Mt DM	286	369	440	482	
Share of food wasted in current LIRs	Percentage	16.41	18.44	18.51	16.57	
Share of food wasted in current MIRs	Percentage	21.94	20.59	18.2	16.21	Similar to GBF indicator for target 16 "Food waste index" and SDG indicator 12.3.1b "food waste index"
Share of food wasted in current HIRs	Percentage	25.75	19.18	17.16	15.38	
Cereal yields with fixed area weights from 2020, global in current LIRs	t DM/ha	2.7	3.5	4.4	4.7	
Cereal yields with fixed area weights from 2020, global in current MIRs	t DM/ha	4.7	5.4	6.5	7.3	
Cereal yields with fixed area weights from 2020, global in current HIRs	t DM/ha	4.9	5.1	5.6	5.8	
Oil crop yields with fixed area weights from 2020, global in current LIRs	t DM/ha	1.5	2	2.8	3	
Oil crop yields with fixed area weights from 2020, global in current MIRs	t DM/ha	3.5	4	4.8	5.4	
Oil crop yields with fixed area weights from 2020, global in current HIRs	t DM/ha	2.4	2.6	2.6	2.7	
Fruits, vergetables and nuts yields with fixed area weights from 2020, global in current LIRs	t DM/ha	1.4	1.8	2.3	2.4	
Fruits, vergetables and nuts yields with fixed area weights from 2020, global in current MIRs	t DM/ha	2.8	3.2	3.9	4.3	
Fruits, vergetables and nuts yields with fixed area weights from 2020, global in current HIRs	t DM/ha	3.9	4.2	4.3	4.7	
Short rotation bioenergy crop yields with fixed area weights from 2020, global in current LIRs	t DM/ha	16.7	20	21	18.2	
Short rotation bioenergy crop yields with fixed area weights from 2020, global in current MIRs	t DM/ha	21.2	23.2	24.5	27.1	
Short rotation bioenergy crop yields with fixed area weights from 2020, global in current HIRs	t DM/ha	19.3	21.4	21.9	23.1	
Feed conversion for ruminant meat and dairy, global in current LIRs	GE per GE	50.8	38.5	32.9	28.5	
Feed conversion for ruminant meat and dairy, global in current MIRs	GE per GE	47.3	38	30.1	21.5	
Feed conversion for ruminant meat and dairy, global in current HIRs	GE per GE	17.2	15.6	13.7	12.9	
Feed conversion for pig meat, global in current LIRs	GE per GE	13	11.1	10.2	9.1	
Feed conversion for pig meat, global in current MIRs	GE per GE	7.7	7.4	7.1	6.8	
Feed conversion for pig meat, global in current HIRs	GE per GE	5.7	5.5	5.3	5.2	
Feed conversion for poultry meat and eggs, global in current LIRs	GE per GE	7.9	7.2	6.7	6.3	
Feed conversion for poultry meat and eggs, global in current MIRs	GE per GE	7.5	6.6	6	5.4	
Feed conversion for poultry meat and eggs, global in current HIRs	GE per GE	6.2	5.9	5.6	5.6	
Area used for bioenergy crops, global	Mha	8	33	70	119	
Area used for fruits, vegetables and nuts, global	Mha	115	137	142	143	
Area used for legumes, global	Mha	180	206	200	189	

Total cropland area, global	Mha	1193	1198	1103	1032	
Irrigated cropland area, global	Mha	116	132	175	175	
Afforestation compared to 2020, global	Mha	0	157	333	424	
Managed forest area, global	Mha	326	512	732	870	
Natural forest area (primary + secondary), global	Mha	3721	3716	3720	3729	
Total forest area, global	Mha	4047	4228	4452	4599	Similar to SDG indicator 15.1.1 “Forest area as a proportion of total land area”
Total other semi-natural vegetation, global	Mha	405	4025	4161	4238	
Timber plantations, global	Mha	149	178	222	269	
Demand for roundwood, global	Mm3/yr	3773	4005	4072	4228	
Rewetted formerly drained peatlands, global	Mha	0	0	31	37	
Protected areas, global	Mha	1699	3490	3483	3482	Similar to GBF indicator target 3 “Protected area coverage of Key Biodiversity Areas”
Croparea diversity Shannon Index in current LIRs	Index	2.23	2.3	2.43	2.55	Similar to GBF target 10 indicator “Agrobiodiversity Index”
Croparea diversity Shannon Index in current MIRs	Index	2.08	2.16	2.23	2.18	
Croparea diversity Shannon Index in current HIRs	Index	2.22	2.22	2.21	2.18	
Inorganic fertilizer on cropland and pastures, global	Mt N/yr	127	108	98	93	GBF indicator for target 7 “Fertilizer use”
Soil Nitrogen Uptake Efficiency (SNUpE), global	Percent	58	65	70	75	
Manure recycling quota, excluding field losses, global	Percent	62	67	71	74	
Nitrogen surplus from cropland, global	Mt N/yr	97	78	64	48	similar to GBF target 7 “Trends in loss of reactive nitrogen to the environment”
Nitrogen surplus from pasture, global	Mt N/yr	37	26	23	16	
Nitrogen surplus from animal waste management, global	Mt N/yr	22	18	13	7	
Nitrogen surplus from semi-natural vegetation, global	Mt N/yr	82	83	88	92	
Soil organic carbon in top 30 cm, global	Gt C	645	648	655	661	Similar to GBF target 10 indicator “Changes in soil organic carbon stocks”
Anthropogenic LUC emissions, global	Gt CO2/yr	3.7	-1.8	-6.4	-8.5	
AFOLU emissions, global	Gt CO2eq/yr	11.6	4	-2.4	-6.2	similar to GBF indicator target 8 “National greenhouse inventories from land use and land use change"
Labor productivity in current LIRs	USD_{05MER}/worker	1711	2556	5322	11313	Connected to SDG target 2.3 “By 2030, double the agricultural productivity”
Labor productivity in current MIRs	USD_{05MER}/worker	4905	6747	10608	15360	
Labor productivity in current HIRs	USD_{05MER}/worker	40895	44461	49374	50024	
Alternative livelihoods needed for people formerly working in agriculture, difference to 2020 in current LIRs	Mio people	0	20	168	277	Connected to SDG target 8.5 “achieve full and productive employment” and

Alternative livelihoods needed for people formerly working in agriculture, difference to 2020 in current MIRs	Mio people	0	91	221	295	target 9.3 “raise industry’s share of employment”
Alternative livelihoods needed for people formerly working in agriculture, difference to 2020 in current HIRs	Mio people	0	3	7	9	
Share of working age population employed in agriculture, global in current LIRs	Percent	24	18	10	4	
Share of working age population employed in agriculture, global in current MIRs	Percent	16	12	8	5	
Share of working age population employed in agriculture, global in current HIRs	Percent	3	3	2	2	
Gini, global	Index	0.42	0.42	0.4	0.38	Connected to SDG goal 10 “Reduce inequality within and among countries”
People below poverty line of $3.20 in current LIRs	Mio people	1327	982	509	177	Similar to SDG goal 1.1. “By 2030, eradicate extreme poverty for all people everywhere, currently measured as people living on less than $1.25 a day”
People below poverty line of $3.20 in current MIRs	Mio people	766	396	147	47	
People below poverty line of $3.20 in current HIRs	Mio people	10	6	4	3	
Deaths attributed to dietary risks in current LIRs	Mio people	2.6	2.1	1.9	1.5	similar to SDG 3 indicator 3.4.1 “Mortality rate attributed to cardiovascular disease, cancer, diabetes or chronic respiratory disease”
Deaths attributed to dietary risks in current MIRs	Mio people	6.6	4.4	3.1	2.3	
Deaths attributed to dietary risks in current HIRs	Mio people	2.8	1.8	1.5	1.3	
Lifetime per capita lost due to dietary and metabolic risks, global	Days per year	13	8	5	4	

Supplementary Table 1

Potential intermediate milestones for achieving the food system transformation in the context of a cross-sectoral sustainable development pathway, and mapping to the aims, targets and indicators of the Sustainable Development Goals (SDGs)[250] and the Global Biodiversity Framework[251]. Moreover, the comprehensive SDG indicator 2.4.1 “Proportion of agricultural area under productive and sustainable agriculture” and its quantification by FAO[252] through various sub-indicators can also be partly mapped to our indicators. LIR, MIR and HIR refers to current low-income, middle-income, and high-income regions (see S1.1.1)

ANZ	Australia & New Zealand	High-income region (HIR)
BRA	Brazil	Middle-income region (MIR)
CAN	Canada	High-income region (HIR)
CHA	China	Middle-income region (MIR)
EUR	European Union	High-income region (HIR)
IND	India	Low-income region (LIR)
JKO	Japan & South Korea	High-income region (HIR)
LAM	Latin America (excl. Brazil)	Middle-income region (MIR)
MEA	Middle East & North Africa	Middle-income region (MIR)
NEA	Northern Eurasia	Middle-income region (MIR)
NEU	Europe (Non-EU)	High-income region (HIR)
OAS	Other Asia	Middle-income region (MIR)
SSA	Sub-Saharan Africa	Low-income region (LIR)
USA	United States of America	High-income region (HIR)

Supplementary Table 2

World regions used for MAgPIE simulations and their classification into low-income, middle-income, and high-income.

Food group	Endpoint	Unit	RR mean	RR low	RR high	Reference
Red meat	CHD	100 g/d	1.15	1.08	1.23	Bechthold et al (2019)
	Stroke	100 g/d	1.12	1.06	1.17	Bechthold et al (2019)
	Colorectal cancer	100 g/d	1.12	1.06	1.19	Schwingshackl et al (2018)
	Type 2 diabetes	100 g/d	1.17	1.08	1.26	Schwingshackl et al (2017)
Fruits	CHD	100 g/d	0.95	0.92	0.99	Aune et al (2017)
	Stroke	100 g/d	0.77	0.70	0.84	Aune et al (2017)
	Cancer	100 g/d	0.94	0.91	0.97	Aune et al (2017)
Vegetables	CHD	100 g/d	0.84	0.80	0.88	Aune et al (2017)
	Cancer	100 g/d	0.93	0.91	0.95	Aune et al (2017)
Legumes	CHD	57 g/d	0.86	0.78	0.94	Afshin et al (2014)
Nuts	CHD	28 g/d	0.71	0.63	0.80	Aune et al (2016)
Underweight	CHD	15<BMI<18.5	1.17	1.09	1.24	Global BMI Collab (2016)
	Stroke	15<BMI<18.5	1.37	1.23	1.53	Global BMI Collab (2016)
	Cancer	15<BMI<18.5	1.10	1.05	1.16	Global BMI Collab (2016)
	Respiratory disease	15<BMI<18.5	2.73	2.31	3.23	Global BMI Collab (2016)
Overweight	CHD	25<BMI<30	1.34	1.32	1.35	Global BMI Collab (2016)
	Stroke	25<BMI<30	1.11	1.09	1.14	Global BMI Collab (2016)
	Cancer	25<BMI<30	1.10	1.09	1.12	Global BMI Collab (2016)
	Respiratory disease	25<BMI<30	0.90	0.87	0.94	Global BMI Collab (2016)
	Type 2 diabetes	25<BMI<30	1.88	1.56	2.11	Prosp Studies Collab (2009)
Obesity (grade 1)	CHD	30<BMI<35	2.02	1.91	2.13	Global BMI Collab (2016)
	Stroke	30<BMI<35	1.46	1.39	1.54	Global BMI Collab (2016)
	Cancer	30<BMI<35	1.31	1.28	1.34	Global BMI Collab (2016)
	Respiratory disease	30<BMI<35	1.16	1.08	1.24	Global BMI Collab (2016)
	Type 2 diabetes	30<BMI<35	3.53	2.43	4.45	Prosp Studies Collab (2009)
Obesity (grade 2)	CHD	30<BMI<35	2.81	2.63	3.01	Global BMI Collab (2016)
	Stroke	30<BMI<35	2.11	1.93	2.30	Global BMI Collab (2016)
	Cancer	30<BMI<35	1.57	1.50	1.63	Global BMI Collab (2016)
	Respiratory disease	30<BMI<35	1.79	1.60	1.99	Global BMI Collab (2016)
	Type 2 diabetes	30<BMI<35	6.64	3.80	9.39	Prosp Studies Collab (2009)
Obesity (grade 3)	CHD	30<BMI<35	3.81	3.47	4.17	Global BMI Collab (2016)
	Stroke	30<BMI<35	2.33	2.05	2.65	Global BMI Collab (2016)
	Cancer	30<BMI<35	1.96	1.83	2.09	Global BMI Collab (2016)
	Respiratory disease	30<BMI<35	2.85	2.43	3.34	Global BMI Collab (2016)
	Type 2 diabetes	30<BMI<35	12.49	5.92	19.82	Prosp Studies Collab (2009)

Supplementary Table 3

Relative risk parameters (mean and low and high values of 95% confidence intervals) for dietary risks and weight-related risks.

Food group	Endpoint	Association	Certainty of evidence
Fruits	CHD	reduction	NutriCoDE: probable or convincing;<br>NutriGrade: moderate quality of meta-evidence
	Stroke	reduction	NutriCoDE: probable or convincing<br>NutriGrade: moderate quality of meta-evidence
	Cancer	reduction	WCRF: strong evidence (probable) for some cancers<br>NutriGrade: moderate quality of meta-evidence for colorectal cancer
Vegetables	CHD	reduction	NutriCoDE: probable or convincing<br>NutriGrade: moderate quality of meta-evidence
	Cancer	reduction	WCRF: strong evidence (probable) for non-starchy vegetables and some cancers<br>NutriGrade: moderate quality of meta-evidence for colorectal cancer
Legumes	CHD	reduction	NutriCoDE: probable or convincing<br>NutriGrade: moderate quality of meta-evidence
Nuts and seeds	CHD	reduction	NutriCoDE: probable or convincing<br>NutriGrade: moderate quality of meta-evidence
Red meat	CHD	increase	NutriGrade: moderate quality of meta-evidence
	Stroke	increase	NutriGrade: moderate quality of meta-evidence
	Cancer	increase	WCRF: strong evidence (probable) for colorectal cancer<br>NutriGrade: moderate quality of meta-evidence for colorectal cancer
	Type-2 diabetes	increase	NutriCoDE: probable or convincing<br>NutriGrade: high quality of meta-evidence

NutriCoDE: Nutrition and Chronic Diseases Expert Group

NutriGrade: Grading of Recommendations Assessment, Dvelopment, and Evaluation (GRADE) tailored to nutrition research

WCRF: World Cancer Research Fund

Supplementary Table 4

Overview of existing ratings on the certainty of evidence for a statistically significant association between a risk factor and a disease endpoint. The ratings include those of the Nutrition and Chronic Diseases Expert Group (NutriCoDE) [179], the World Cancer Research Fund[181], and NutriGrade[110–112]. The ratings relate to the risk-disease associations in general, and not to the specific relative-risk factor used for those associations in this analysis.

Country	Base Year	Target Year NPI	NPI Target (Mha)	Target Year NDC	NDC Target (Mha)
China	1995	2020	40	2030	138
India	2005	2020	5	2030	31.7
Brazil	2010	2020	3	2030	12
The EU	1995	2020	9.5	2020	9.5
USA	1995	2015	7.1	2015	7.1
Ethiopia	2020	NA	NA	2030	7
Bolivia	2020	NA	NA	2030	4.5
Thailand	2020	NA	NA	2030	3.69

Supplementary Table 5

List of National Policies Implemented (NPI) and Nationally Determined Contributions (NDCs) for countries with major targets for afforestation policy (in Mha).

Model	Publication	Focus of validation	Scenarios	Validation type	
				Comparison with historical data / trends	Comparison to other model projections
MAgPIE	Stehfest et al (2019)[253]	Cropland projections, pasture projections, crop production projections	Baseline scenarios		x
	Bodirsky et al (2012)[254]	N2O emission projections, fertilizer projections	Baseline scenarios	x	x
		Manure excretion, biological fixation, residues, nitrogen surplus, crop harvest	Current state	x	
	Bodirsky, Leclère et al (accepted)[255]	Manure excretion, biological fixation, atmospheric deposition, nitrogen surplus, inorganic fertilizer, crop harvest, manure management	Different SSPs with different nitrogen mitigation measures based on Kanter et al (2020)	x	x
	Popp et al (2017)[7]	Demand, production and trade for crops, livestock and bioenergy, cropland area, bioenergy area, pasture area, forests, other natural land, cereal crop yields, CO_2, CH_4 and N_2O emissions, agricultural commodity prices,	5 SSP baseline scenarios, 2*5 GHG mitigation scenarios	x	x
	Doelmann et al (2022)[29]	Water withdrawal, Landuse change, food prices, AFOLU emissions, nitrogen surplus	Baseline scenario,<br>water protection, biodiversity protection, fertilizer efficiency, diet change, food waste, GHG price, bioenergy production, climate impacts		x
	Leclere et al (2020)[28]	BII	Baseline scenarios,<br>biodiversity protection scenarios	x	x
	Humpenöder et al (2022)[256]	Population, income, livestock demand and production, land-use change, water withdrawal, AFOLU GHG emissions, nitrogen fixation	Baseline scenario,<br>ruminant meat substitution scenarios	x	
	Humpenöder et al (2022)[31]	Population, Income, Per-capita calorie supply, Crop and Livestock demand, land-use change, AFOLU GHG emissions, nitrogen fixation	GHG mitigation scenarios	x	
	Von Jeetze et al. (2023)[205]	Agricultural price index, forest cover, grassland cover, other non-forest vegetation cover, cropland area, annual average yield changes, CO2 emissions from land-use change	Biodiversity conservation and GHG mitigation scenarios	x	
	Weindl et al (2017)[95]	Cropland projections, pasture projections, land-use intensity	Baseline scenario, livestock productivity scenarios, diet change and food waste scenario	x	
	Bonsch et al 2015[49]	Water and environmental flow requirements (EFR)	SRES scenarios A2 and B1;<br>3 different EFR scenarios (Hanasaki, Smakthin, Tennant methods)	x	

	Molina Bacca et al 2023[257]	Crop yields under climate change and repercussions in land system adaptation	SSP1-RCP2.6 and SSP5-RCP8,5		x
	Leip et al, in preparation[157]	Agricultural labor demand, wages, labor costs in crop and livestock production, labor-to-capital ratio	SSP1-5	x	
	Mishra et al. 2021[97]	Plantation rotation lengths, industrial roundwood and wood fuel demand, forest growing stocks, forest plantation area, annual land-use change emissions, land-use intensity	SSP2, MAgPIE with forestry	x	x
	Fujimori et al., 2019[76]	Change in agricultural prices, CO2 emissions, N2O emissions, CH4 emissions	SSP2-NDC, 2° mitigation target, 1.5° mitigation target		x
Food demand model	Bodirsky et al (2020)[2], SI	Food demand and composition, underweight, overweight, obesity	Baseline scenarios	x	x
	Fujimori et al., 2019[76]	Population at risk of hunger, per-capita food consumption	Baseline scenarios	x	x
LPJmL	Schaphoff et al. 2018[32]	Carbon fluxes, carbon stocks, permafrost extent, burnt area, river discharge, evapotranspiration, crop yields		x	
	von Bloh et al. 2018[33]	Nitrogen stocks and fluxes		x	x
	Müller et al. 2017[258]	Crop yields		x	x
	Jägermeyer et al 2021[259]	Crop yields under climate change			x
REMIN D	Baumstark et al. 2021[37]	Global primary energy consumption of different energy carriers, global total and sectoral final energy, population, GDP, regional GHG emissions	Baseline & policy scenarios	x	
	Luderer et al. 2019[260]	Electricity generation	Baseline & policy scenarios		x
	Bauer et al. 2018[261]	Bioenergy use	Baseline & policy scenarios		x
	Luderer et al. 2018[262]	Global total and sectoral fossil emissions, global total emissions, carbon price, global total and sectoral final energy, electricity demand	Policy scenarios		x
Poverty model	Soergel et al. 2021[36]	National and global poverty headcount data (3 poverty lines)	Baseline & policy scenarios	x	(x)
MAGIC C	IPCC AR6 WG1 Cross-Chapter Box 7.1[116]	Response across range of IPCC assessment (temperature projections, key climate metrics)	Emulation of historical warming, emissions scenarios (SSP1-1.9 – SSP5-8.5)	x	x
	Meinshausen et al. (2011)[88]	Global-mean surface temperature, heat uptake, effective radiative forcing, effective climate sensitivity, land-ocean warming ratio, terrestrial C-uptake, oceanic C-uptake, gross primary production, terrestrial living C-pool, terrestrial dead C-pool, total respiration,	SRES scenarios; CMIP3 AOGCM idealized scenarios	x	x

		CO_2 concentrations			
	Meinshausen et al. (2020)[34]	Greenhouse gas concentrations, radiative forcing, surface air temperatures with seasonal and regional differentiation	SSP1-5	x	
MRI-ESM-2-0	Yukimoto, S., et al. 2019[38]	Climate drift (surface air temperature); Present-day: climate radiation (shortwave, longwave), surface air temperature, implied ocean heat transport, present-day precipitation, atmospheric circulation, sea ice, ocean surface temperature, ocean interior temperature and salinity, El Niño Southern Oscillation, Stratospheric Quasi-Biennial Oscillation; Historical climate change	CMIP6 historical simulations, using CMIP6 forcing dataset version 6.2.1	x	x
Dietary health model	Willet et al (2019)[6]	Premature mortality	Effects of the adoption of the PhD diet, using a comparative risk model, the global burden of diseases model, and an empirical disease risk model		x

Supplementary Table 6

Previous studies of the models involved in this analysis in which results have been validated with historical data/trends or compared to other models.

References Supplementary Materials

145. Dietrich, J. P., Popp, A. & Lotze-Campen, H. Reducing the loss of information and gaining accuracy with clustering methods in a global land-use model. *Ecological Modelling* **263**, 233–243 (2013).

146. FAOSTAT. Database collection of the Food and Agriculture Organization of the United Nations. www.fao.org/faostat (2016).

147. IEA. World Energy Balances 2022. https://www.iea.org/data-and-statistics/data-product/world-energy-balances (2022).

148. Institute for Bioplastics and Biocomposites. Biopolymers Facts and Statistics 2021. (2021).

149. Lauri, P. *et al.* Global Woody Biomass HarvestVolumes and Forest Area Use UnderDifferent SSP-RCP Scenarios. *JfE* **34**, 285–309 (2019).

150. Skoczinski, P. *et al.* Bio-Based Building Blocks and Polymers – Global Capacities, Production and Trends 2020 – 2025 (Short Version). (2021).

151. Sannigrahi, P., Ragauskas, A. J. & Tuskan, G. A. Poplar as a Feedstock for Biofuels: A Review of Compositional Characteristics. *Biofuels, Bioproducts and Biorefining* **4**, 209–226 (2010).

152. Amarasekara, A. S. *Handbook of Cellulosic Ethanol*. (John Wiley & Sons, 2013).

153. Ribeiro, R. A., Colodette, J. L. & Júnior, S. V. Effect of residual effective alkali on eucalyptus kraft pulp yield and chemistry. *CERNE* **24**, 408–419 (2018).

154. Wirsenius, S. *Human Use of Land and Organic Materials*. (Chalmers University of Technology and Gäteborg University, Göteborg, Sweden, 2000).

155. O'Neill, B. C. *et al.* The roads ahead: Narratives for shared socioeconomic pathways describing world futures in the 21st century. *Global Environmental Change* https://doi.org/10.1016/j.gloenvcha.2015.01.004 (2015) doi:10.1016/j.gloenvcha.2015.01.004.

156. *Global Trade, Assistance, and Production: The GTAP 7 Data Base*. (2008).

157. Leip, D. *et al.* Future Employment in Global Agriculture: Trends and Drivers in the Shared Socioeconomic Pathways. (in prep).

158. Fuglie, K. Accounting for growth in global agriculture. *Bio-based and Applied Economics* **Vol 4 No 3**, 201-234 Pages (2015).

159. USDA. International Agricultural Productivity -- Machine-Readable and Long Format File of TFP Indices and Components for Countries, Regions, Countries Grouped by Income Level, and the World, 1961–2016. https://www.ers.usda.gov/data-products/international-agricultural-productivity/ (2021).

160. International Labour Organization. ILOSTAT. *ILOSTAT* https://ilostat.ilo.org/data/ (2023).

161. Orlov, A. *et al.* Global Economic Responses to Heat Stress Impacts on Worker Productivity in Crop Production. *EconDisCliCha* **5**, 367–390 (2021).

162. Rockström, J. *et al.* A safe operating space for humanity. *Nature* **461**, 472--475 (2009).

163. Klasen, S. *et al.* Economic and ecological trade-offs of agricultural specialization at different spatial scales. *Ecological Economics* **122**, 111–120 (2016).

164. Seifert, C. A., Roberts, M. J. & Lobell, D. B. Continuous Corn and Soybean Yield Penalties across Hundreds of Thousands of Fields. *Agronomy Journal* **109**, 541–548 (2017).

165. Landwirtschaftskammer NRW. Maximale Anbaukonzentration von Feldfruchtarten bzw. -gruppen, nach Zielen der nachhaltigen Pflanzenproduktion in %. (nach Baeumer 1990). (2015).

166. Schönhart, M., Schmid, E. & Schneider, U. A. CropRota – A crop rotation model to support integrated land use assessments. *European Journal of Agronomy* **34**, 263–277 (2011).

167. Escasinas, A. B. & Escalada, R. G. Crop Rotation of Sweet Potato, Cassava, and Gabi with Legumes as a Cultural Management System. *Annals of Tropical Research* **6**, (1984).

168. Scholes, R. J. & Biggs, R. A biodiversity intactness index. *Nature* **434**, 45–49 (2005).

169. IPCC. *2019 Refinement to the 2006 IPCC Guidelines for National Greenhouse Gas Inventories*. https://www.ipcc.ch/report/2019-refinement-to-the-2006-ipcc-guidelines-for-national-greenhouse-gas-inventories/ (2019).

170. McDuffie, E. E. *et al. A Global Anthropogenic Emission Inventory of Atmospheric Pollutants from Sector- and Fuel-Specific Sources (1970–2017): An Application of the Community Emissions Data System (CEDS)*. https://essd.copernicus.org/preprints/essd-2020-103/essd-2020-103.pdf (2020) doi:10.5194/essd-2020-103.

171. Günther, A. *et al.* Prompt rewetting of drained peatlands reduces climate warming despite methane emissions. *Nat Commun* **11**, 1644 (2020).

172. IPCC. *2006 IPCC Guidelines for National Greenhouse Gas Inventories, Prepared by the National Greenhouse Gas Inventories Programme*. (2006).

173. Daalen, K. R. van *et al.* The 2022 Europe report of the Lancet Countdown on health and climate change: towards a climate resilient future. *The Lancet Public Health* **7**, e942–e965 (2022).

174. Lim, S. S. *et al.* A comparative risk assessment of burden of disease and injury attributable to 67 risk factors and risk factor clusters in 21 regions, 1990–2010: a systematic analysis for the Global Burden of Disease Study 2010. *The Lancet* **380**, 2224–2260 (2012).

175. Forouzanfar, M. H. *et al.* Global, regional, and national comparative risk assessment of 79 behavioural, environmental and occupational, and metabolic risks or clusters of risks in 188 countries, 1990–2013: a systematic analysis for the Global Burden of Disease Study 2013. *The Lancet* **386**, 2287–2323 (2015).

176. Murray, C. J. L. *et al.* GBD 2010: design, definitions, and metrics. *Lancet* **380**, 2063–2066 (2012).

177. NCD-RisC. Trends in adult body-mass index in 200 countries from 1975 to 2014: a pooled analysis of 1698 population-based measurement studies with 19·2 million participants. *The Lancet* **387**, 1377–1396 (2016).

178. Singh, G. M. *et al.* The age-specific quantitative effects of metabolic risk factors on cardiovascular diseases and diabetes: a pooled analysis. *PLoS One* **8**, e65174 (2013).

179. Micha, R. *et al.* Etiologic effects and optimal intakes of foods and nutrients for risk of cardiovascular diseases and diabetes: Systematic reviews and meta-analyses from the Nutrition and Chronic Diseases Expert Group (NutriCoDE). *PLoS One* **12**, e0175149 (2017).

180. Schwingshackl, L. *et al.* Perspective: NutriGrade: A Scoring System to Assess and Judge the Meta-Evidence of Randomized Controlled Trials and Cohort Studies in Nutrition Research. *Adv Nutr* **7**, 994–1004 (2016).

181. World Cancer Research Fund & American Institute for Cancer Research. *Diet, Nutrition, Physical Activity and Cancer: A Global Perspective. Continuous Update Project Expert Report.* https://www.wcrf.org/wp-content/uploads/2021/02/Summary-of-Third-Expert-Report-2018.pdf (2018).

182. Aune, D. *et al.* Dairy products and colorectal cancer risk: a systematic review and meta-analysis of cohort studies. *Ann Oncol* **23**, 37–45 (2012).

183. Aune, D., Norat, T., Romundstad, P. & Vatten, L. J. Dairy products and the risk of type 2 diabetes: a systematic review and dose-response meta-analysis of cohort studies. *Am J Clin Nutr* **98**, 1066–1083 (2013).

184. Mohan, D. *et al.* Associations of Fish Consumption With Risk of Cardiovascular Disease and Mortality Among Individuals With or Without Vascular Disease From 58 Countries. *JAMA Intern Med* **181**, 631–649 (2021).

185. Prospective Studies Collaboration *et al.* Body-mass index and cause-specific mortality in 900 000 adults: collaborative analyses of 57 prospective studies. *Lancet* **373**, 1083–1096 (2009).

186. Zheng, J. *et al.* Fish consumption and CHD mortality: an updated meta-analysis of seventeen cohort studies. *Public Health Nutr* **15**, 725–737 (2012).

187. Zhao, L.-G. *et al.* Fish consumption and all-cause mortality: a meta-analysis of cohort studies. *Eur J Clin Nutr* **70**, 155–161 (2016).

188. Xun, P. *et al.* Fish consumption and risk of stroke and its subtypes: accumulative evidence from a meta-analysis of prospective cohort studies. *Eur J Clin Nutr* **66**, 1199–1207 (2012).

189. Jayedi, A., Shab-Bidar, S., Eimeri, S. & Djafarian, K. Fish consumption and risk of all-cause and cardiovascular mortality: a dose-response meta-analysis of prospective observational studies. *Public Health Nutr* **21**, 1297–1306 (2018).

190. Guasch-Ferré, M. *et al.* Meta-Analysis of Randomized Controlled Trials of Red Meat Consumption in Comparison With Various Comparison Diets on Cardiovascular Risk Factors. *Circulation* **139**, 1828–1845 (2019).

191. Schwingshackl, L. *et al.* Intake of 12 food groups and disability-adjusted life years from coronary heart disease, stroke, type 2 diabetes, and colorectal cancer in 16 European countries. *Eur J Epidemiol* **34**, 765–775 (2019).

192. FAO. *Food Balance Sheets - A Handbook*. https://www.fao.org/3/x9892e/x9892e.pdf (2001).

193. Del Gobbo, L. C. *et al.* Assessing global dietary habits: a comparison of national estimates from the FAO and the Global Dietary Database. *Am J Clin Nutr* **101**, 1038–1046 (2015).

194. Micha, R. *et al.* Global, regional and national consumption of major food groups in 1990 and 2010: a systematic analysis including 266 country-specific nutrition surveys worldwide. *BMJ Open* **5**, e008705 (2015).

195. Freedman, L. S. *et al.* Pooled results from 5 validation studies of dietary self-report instruments using recovery biomarkers for energy and protein intake. *Am J Epidemiol* **180**, 172–188 (2014).

196. Rennie, K. L., Coward, A. & Jebb, S. A. Estimating under-reporting of energy intake in dietary surveys using an individualised method. *Br J Nutr* **97**, 1169–1176 (2007).

197. Wang, X. *et al.* Taking account of governance: Implications for land-use dynamics, food prices, and trade patterns. *Ecological Economics* **122**, 12–24 (2016).
198. UNFCC. Nationally Determined Contributions Registry. *UNFCC* https://unfccc.int/NDCREG (2022).
199. Swinburn, B. *et al.* Monitoring and benchmarking government policies and actions to improve the healthiness of food environments: a proposed Government Healthy Food Environment Policy Index. *Obesity Reviews* **14**, 24–37 (2013).
200. Swinburn, B. A. *et al.* The Global Syndemic of Obesity, Undernutrition, and Climate Change: The Lancet Commission report. *The Lancet* **393**, 791–846 (2019).
201. Chakrabarti, A., Ellermeier, N., Tripathi, A., Thirumurthy, H. & Nugent, R. Diet-focused behavioral interventions to reduce the risk of non-communicable diseases in low- and middle-income countries: A scoping review of existing evidence. *Obesity Reviews* **26**, e13918 (2025).
202. Schmitz, C. *et al.* Trading more food: Implications for land use, greenhouse gas emissions, and the food system. *Global Environmental Change* **22**, 189--209 (2012).
203. Aaheim, A., Orlov, A., Wei, T. & Glomsrød, S. *GRACE Model and Applications*. 46 https://pub.cicero.oslo.no/cicero-xmlui/bitstream/handle/11250/2480843/GRACE%20webversjon.pdf?sequence=1&isAllowed=y (2018).
204. Kreidenweis, U. *et al.* Afforestation to mitigate climate change: impacts on food prices under consideration of albedo effects. *Environ. Res. Lett.* **11**, 085001 (2016).
205. von Jeetze, P. J. *et al.* Projected landscape-scale repercussions of global action for climate and biodiversity protection. *Nat Commun* **14**, 2515 (2023).
206. Keys, P. W., Collins, P. M., Chaplin-Kramer, R. & Wang-Erlandsson, L. Atmospheric water recycling an essential feature of critical natural asset stewardship. *Global Sustainability* **7**, e2 (2024).

207. Windisch, M. G. *et al.* Accounting for local temperature effect substantially alters afforestation patterns. *Environ. Res. Lett.* **17**, 024030 (2022).

208. Greifswald Mire Centre. The Global Peatland Database. https://greifswaldmoor.de/global-peatland-database-en.html (2022).

209. IPCC. *2013 Supplement to the 2006 IPCC Guidelines for National Greenhouse Gas Inventories: Wetlands*. https://www.ipcc-nggip.iges.or.jp/public/wetlands/pdf/Wetlands_Supplement_Entire_Report.pdf (2013).

210. Wilson, D., Blain, D. & Couwenberg, J. Greenhouse gas emission factors associated with rewetting of organic soils. *Mires and Peat* 1–28 (2016) doi:10.19189/MaP.2016.OMB.222.

211. Tiemeyer, B. *et al.* A new methodology for organic soils in national greenhouse gas inventories: Data synthesis, derivation and application. *Ecological Indicators* **109**, 105838 (2020).

212. Pastor, A. V., Ludwig, F., Biemans, H., Hoff, H. & Kabat, P. Accounting for environmental flow requirements in global water assessments. *Hydrology and Earth System Sciences* **18**, 5041–5059 (2014).

213. Olson, D. M. *et al.* Terrestrial Ecoregions of the World: A New Map of Life on Earth: A new global map of terrestrial ecoregions provides an innovative tool for conserving biodiversity. *BioScience* **51**, 933–938 (2001).

214. de Vries, W. Impacts of nitrogen emissions on ecosystems and human health: A mini review. *Current Opinion in Environmental Science & Health* **21**, 100249 (2021).

215. Wang, M. *et al.* A triple increase in global river basins with water scarcity due to future pollution. *Nat Commun* **15**, 880 (2024).

216. Oelmann, D. M. *et al.* Quantifizierung der landwirtschaftlich verursachten Kosten zur Sicherung der Trinkwasserbereitstellung. 252 (2017).

217. Garibaldi, L. A. *et al.* Working landscapes need at least 20% native habitat. *Conservation Letters* **14**, e12773 (2021).

218. Dainese, M. *et al.* A global synthesis reveals biodiversity-mediated benefits for crop production. *Science Advances* **5**, eaax0121 (2019).

219. Mohamed, A. *et al.* Securing Nature's Contributions to People requires at least 20%–25% (semi-)natural habitat in human-modified landscapes. *One Earth* **7**, 59–71 (2024).

220. Tscharntke, T., Grass, I., Wanger, T. C., Westphal, C. & Batáry, P. Beyond organic farming – harnessing biodiversity-friendly landscapes. *Trends in Ecology & Evolution* **36**, 919–930 (2021).

221. Rockström, J. *et al.* Safe and just Earth system boundaries. *Nature* 1–10 (2023) doi:10.1038/s41586-023-06083-8.

222. Buchhorn, M. *et al.* Copernicus Global Land Service: Land Cover 100m: collection 2: epoch 2015: Globe. [object Object] https://doi.org/10.5281/ZENODO.3243509 (2019).

223. Steinfeld, H. & Gerber, P. Livestock production and the global environment: Consume less or produce better? *Proceedings of the National Academy of Sciences* **107**, 18237–18238 (2010).

224. Herrero, M. *et al.* Biomass use, production, feed efficiencies, and greenhouse gas emissions from global livestock systems. *PNAS* **110**, 20888–20893 (2013).

225. Guo, L. B. & Gifford, R. M. Soil carbon stocks and land use change: a meta analysis. *Global Change Biology* **8**, 345–360 (2002).

226. Trost, B. *et al.* Irrigation, soil organic carbon and N2O emissions. A review. *Agron. Sustain. Dev.* **33**, 733–749 (2013).

227. Frank, S. *et al.* Reducing greenhouse gas emissions in agriculture without compromising food security? *Environmental Research Letters* **12**, 105004 (2017).

228. Koch, J. & Leimbach, M. SSP economic growth projections: Major changes of key drivers in integrated assessment modelling. *Ecological Economics* **206**, 107751 (2023).

229. Daioglou, V. *et al.* Bioenergy technologies in long-run climate change mitigation: results from the EMF-33 study. *Climatic Change* **163**, 1603–1620 (2020).

230. Raymond, C. *et al.* Understanding and managing connected extreme events. *Nat. Clim. Chang.* **10**, 611–621 (2020).

231. Kotz, M., Levermann, A. & Wenz, L. The economic commitment of climate change. *Nature* **628**, 551–557 (2024).

232. Churkina, G. *et al.* Buildings as a global carbon sink. *Nat Sustain* **3**, 269–276 (2020).

233. Worldbank. Poverty headcount ratio at $3.20 a day (2011 PPP). https://data.worldbank.org/indicator/SI.POV.LMIC.GC (2023).

234. Phillips, H. *et al.* The Biodiversity Intactness Index - country, region and global-level summaries for the year 1970 to 2050 under various scenarios. https://doi.org/10.5519/he1eqmg1 (2021) doi:10.5519/he1eqmg1.

235. Ostberg, S., Müller, C., Heinke, J. & Schaphoff, S. LandInG 1.0: a toolbox to derive input datasets for terrestrial ecosystem modelling at variable resolutions from heterogeneous sources. *Geoscientific Model Development* **16**, 3375–3406 (2023).

236. Tubiello, F. N. *et al.* Pre- and post-production processes along supply chains increasingly dominate GHG emissions from agri-food systems globally and in most countries. *Earth System Science Data Discussions* 1–24 (2021) doi:10.5194/essd-2021-389.

237. WORLDBANK. World Development Indicators. http://data.worldbank.org/data-catalog/world-development-indicators (2013).

238. James, S. L., Gubbins, P., Murray, C. J. & Gakidou, E. Developing a comprehensive time series of GDP per capita for 210 countries from 1950 to 2015. *Population Health Metrics* **10**, 12 (2012).

239. FAOSTAT. Database collection of the Food and Agriculture Organization of the United Nations. www.fao.org/faostat (2023).

240. FAOSTAT. Database collection of the Food and Agriculture Organization of the United Nations. www.fao.org/faostat (2021).

241. Stefan Siebert, Verena Henrich, Karen Frenken, & Jacob Burke. *Global Map of Irrigation Areas Version 5*. (Rheinische Friedrich-Wilhelms-University and , and (2013). . , / Food and Agriculture Organization of the United Nations, Bonn, Germany and Rome, Italy, 2013).

242. Siebert, S. *et al.* Historical Irrigation Dataset (HID). https://doi.org/10.13019/M20599 (2015) doi:10.13019/M20599.

243. Mehta, P. *et al.* Half of twenty-first century global irrigation expansion has been in water-stressed regions. *Nat Water* **2**, 254–261 (2024).

244. Gosling, S. N. *et al.* ISIMIP2b Simulation Data from the Global Water Sector. (2023) doi:10.48364/ISIMIP.626689.

245. Dietrich, J. P. *et al.* Measuring agricultural land-use intensity – A global analysis using a model-assisted approach. *Ecological Modelling* **232**, 109–118 (2012).

246. FAO. *Global Forest Resources Assessment 2015*. http://www.fao.org/docrep/013/i1757e/i1757e.pdf (2015).

247. Lassaletta, L., Billen, G., Grizzetti, B., Angalde, J. & Garnier, J. 50 year trends in nitrogen use efficiency of world cropping systems: the relationship between yield and nitrogen input to cropland. *Environmental Research Letters* (2014).

248. Crippa, M. *et al. Gridded Emissions of Air Pollutants for the Period 1970–2012 within EDGAR v4.3.2*. https://essd.copernicus.org/preprints/essd-2018-31/essd-2018-31.pdf (2018) doi:10.5194/essd-2018-31.

249. Gasser, T. *et al.* Historical CO_2 emissions from land-use and land-cover change and their uncertainty. *Biogeosciences Discussions* 1–43 (2020) doi:https://doi.org/10.5194/bg-2020-33.

250. UN. Global indicator framework for the Sustainable Development Goals and targets of the 2030 Agenda for Sustainable Development. (2017).

251. UNEP. Cunming-Montreal Global Biodiversity Framework. https://www.cbd.int/doc/c/e6d3/cd1d/daf663719a03902a9b116c34/cop-15-l-25-en.pdf (2022).

252. FAO. *Measuring Progress towards Sustainable Agriculture*. (FAO, 2021). doi:10.4060/cb4549en.

253. Stehfest, E. *et al.* Key determinants of global land-use projections. *Nat Commun* **10**, 2166 (2019).

254. Bodirsky, B. L. *et al.* Current state and future scenarios of the global agricultural nitrogen cycle. *Biogeosciences Discuss.* **9**, 2755–2821 (2012).

255. Bodirsky, B. L. *et al.* Assessment of global scale total nitrogen budgets. in *International Nitrogen Assessment* (Cambridge University Press, Cambridge, accepted).

256. Humpenöder, F. *et al.* Projected environmental benefits of replacing beef with microbial protein. *Nature* **605**, 90–96 (2022).

257. Molina Bacca, E. J. *et al.* Uncertainty in land-use adaptation persists despite crop model projections showing lower impacts under high warming. *Commun Earth Environ* **4**, 1–13 (2023).

258. Müller, C. *et al.* Global gridded crop model evaluation: benchmarking, skills, deficiencies and implications. *Geoscientific Model Development* **10**, 1403–1422 (2017).

259. Jägermeyr, J. *et al.* Climate impacts on global agriculture emerge earlier in new generation of climate and crop models. *Nat Food* 1–13 (2021) doi:10.1038/s43016-021-00400-y.

260. Luderer, G. *et al.* Environmental co-benefits and adverse side-effects of alternative power sector decarbonization strategies. *Nat Commun* **10**, 1–13 (2019).

261. Bauer, N., McGlade, C., Hilaire, J. & Ekins, P. Divestment prevails over the green paradox when anticipating strong future climate policies. *Nature Clim Change* **8**, 130–134 (2018).

262. Luderer, G. *et al.* Residual fossil CO2 emissions in 1.5–2 °C pathways. *Nature Clim Change* **8**, 626–633 (2018).
