# Supplementary material for: A food system transformation pathway reconciles 1.5 °C global warming with improved health, environment and social inclusion
Source: Nat Food. 2025 Dec 19;6(12):1133–52. doi: 10.1038/s43016-025-01268-y (PMC12716996; doi:10.1038/s43016-025-01268-y)
Supplement: Supplementary file 2 — Reporting Summary [file 43016_2025_1268_MOESM2_ESM.pdf]

nature portfolio

Corresponding author(s): Benjamin Leon Bodirsky

Last updated by author(s): Oct 30, 2025

Reporting Summary

Nature Portfolio wishes to improve the reproducibility of the work that we publish. This form provides structure for consistency and transparency in reporting. For further information on Nature Portfolio policies, see our Editorial Policies and the Editorial Policy Checklist.

Statistics

For all statistical analyses, confirm that the following items are present in the figure legend, table legend, main text, or Methods section.

n/a	Confirmed	
☐	☒	The exact sample size (*n*) for each experimental group/condition, given as a discrete number and unit of measurement
☒	☐	A statement on whether measurements were taken from distinct samples or whether the same sample was measured repeatedly
☒	☐	The statistical test(s) used AND whether they are one- or two-sided<br>*Only common tests should be described solely by name; describe more complex techniques in the Methods section.*
☒	☐	A description of all covariates tested
☒	☐	A description of any assumptions or corrections, such as tests of normality and adjustment for multiple comparisons
☐	☒	A full description of the statistical parameters including central tendency (e.g. means) or other basic estimates (e.g. regression coefficient) AND variation (e.g. standard deviation) or associated estimates of uncertainty (e.g. confidence intervals)
☒	☐	For null hypothesis testing, the test statistic (e.g. *F*, *t*, *r*) with confidence intervals, effect sizes, degrees of freedom and *P* value noted<br>*Give P values as exact values whenever suitable.*
☒	☐	For Bayesian analysis, information on the choice of priors and Markov chain Monte Carlo settings
☒	☐	For hierarchical and complex designs, identification of the appropriate level for tests and full reporting of outcomes
☒	☐	Estimates of effect sizes (e.g. Cohen's *d*, Pearson's *r*), indicating how they were calculated

Our web collection on statistics for biologists contains articles on many of the points above.

Software and code

Policy information about availability of computer code

Data collection	For reproduction, a package containing a copy of the used versions of MAgPIE, the food demand model, MAGICC, and the m4fsdp R package is available under the Creative Commons Attribution license CC-BY-4.0 and archived at http://doi.org/10.5281/zenodo.17233328.<br>This package also includes all input data for the MAgPIE model and the food demand model including model input from the LPJmL and REMIND models, the raw results of MAgPIE model and food demand model runs, raw results of the MAGICC model, raw results of the health impacts model, derived intermediate analysis data, the final figures, as well as instructions to reproduce any of these results.
Data analysis	For processing the model results, the m4fsdp R package has been used which is available at https://doi.org/10.5281/zenodo.7899913. A copy of the used version of m4fsdp is also included in the data package available under http://doi.org/10.5281/zenodo.17233328.

For manuscripts utilizing custom algorithms or software that are central to the research but not yet described in published literature, software must be made available to editors and reviewers. We strongly encourage code deposition in a community repository (e.g. GitHub). See the Nature Portfolio guidelines for submitting code & software for further information.

Data

Policy information about availability of data

All manuscripts must include a data availability statement. This statement should provide the following information, where applicable:

- Accession codes, unique identifiers, or web links for publicly available datasets
- A description of any restrictions on data availability
- For clinical datasets or third party data, please ensure that the statement adheres to our policy

A data package is available under the Creative Commons Attribution license CC-BY-4.0 and archived at https://doi.org/10.5281/zenodo.7924160 . This package includes all input data for the MAgPIE model, the raw results of MAgPIE model runs, raw results of the MAGICC model, raw results of the health impacts model, derived intermediate analysis data, the final figures, as well as instructions to reproduce any of these results.

Research involving human participants, their data, or biological material

Policy information about studies with human participants or human data. See also policy information about sex, gender (identity/presentation), and sexual orientation and race, ethnicity and racism.

Reporting on sex and gender	No data on sex or gender was reported.
Reporting on race, ethnicity, or other socially relevant groupings	see above
Population characteristics	see above
Recruitment	see above
Ethics oversight	see above

Note that full information on the approval of the study protocol must also be provided in the manuscript.

Field-specific reporting

Please select the one below that is the best fit for your research. If you are not sure, read the appropriate sections before making your selection.

☐ Life sciences ☐ Behavioural & social sciences ☒ Ecological, evolutionary & environmental sciences

For a reference copy of the document with all sections, see nature.com/documents/nr-reporting-summary-flat.pdf

Ecological, evolutionary & environmental sciences study design

All studies must disclose on these points even when the disclosure is negative.

Study description	The presented study uses a set of linked models for integrated assessment to quantitatively evaluate different food system scenarios.
Research sample	As a model-based study, no research sample was selected, and no new additional data was collected.
Sampling strategy	No new data collected
Data collection	No new data collected. Data sources for the models are documented in the underlying model description papers.
Timing and spatial scale	No new data collected
Data exclusions	No new data collected
Reproducibility	For reproduction, a package containing a copy of the used versions of MAgPIE, MAGICC, and the m4fsdp R package is available under the Creative Commons Attribution license CC-BY-4.0 and archived at http://doi.org/10.5281/zenodo.17233328.
Randomization	No new data collected
Blinding	No new data collected

Did the study involve field work? ☐ Yes ☒ No

Reporting for specific materials, systems and methods

We require information from authors about some types of materials, experimental systems and methods used in many studies. Here, indicate whether each material, system or method listed is relevant to your study. If you are not sure if a list item applies to your research, read the appropriate section before selecting a response.

Materials & experimental systems

n/a	Involved in the study
☒	☐ Antibodies
☒	☐ Eukaryotic cell lines
☒	☐ Palaeontology and archaeology
☒	☐ Animals and other organisms
☒	☐ Clinical data
☒	☐ Dual use research of concern
☒	☐ Plants

Methods

n/a	Involved in the study
☒	☐ ChIP-seq
☒	☐ Flow cytometry
☒	☐ MRI-based neuroimaging

Plants

Seed stocks	N/A
Novel plant genotypes	N/A
Authentication	N/A
